# To ingest or rest? Specialized roles of lateral hypothalamic area neurons in coordinating energy balance

**DOI:** 10.3389/fnsys.2015.00009

**Published:** 2015-02-18

**Authors:** Juliette A. Brown, Hillary L. Woodworth, Gina M. Leinninger

**Affiliations:** ^1^Department of Pharmacology and Toxicology, Michigan State UniversityEast Lansing, MI, USA; ^2^Center for Integrative ToxicologyEast Lansing, MI, USA; ^3^Department of Physiology, Michigan State UniversityEast Lansing, MI, USA

**Keywords:** lateral hypothalamic area, orexin, melanin concentrating hormone, neurotensin, dopamine, feeding, obesity

## Abstract

Survival depends on an organism’s ability to sense nutrient status and accordingly regulate intake and energy expenditure behaviors. Uncoupling of energy sensing and behavior, however, underlies energy balance disorders such as anorexia or obesity. The hypothalamus regulates energy balance, and in particular the lateral hypothalamic area (LHA) is poised to coordinate peripheral cues of energy status and behaviors that impact weight, such as drinking, locomotor behavior, arousal/sleep and autonomic output. There are several populations of LHA neurons that are defined by their neuropeptide content and contribute to energy balance. LHA neurons that express the neuropeptides melanin-concentrating hormone (MCH) or orexins/hypocretins (OX) are best characterized and these neurons play important roles in regulating ingestion, arousal, locomotor behavior and autonomic function via distinct neuronal circuits. Recently, another population of LHA neurons containing the neuropeptide Neurotensin (Nts) has been implicated in coordinating anorectic stimuli and behavior to regulate hydration and energy balance. Understanding the specific roles of MCH, OX and Nts neurons in harmonizing energy sensing and behavior thus has the potential to inform pharmacological strategies to modify behaviors and treat energy balance disorders.

## The periphery and the brain act in concert to regulate energy balance

Food and water are essential for survival, and organisms have developed physiological systems to ensure that the body maintains sufficient stores of these resources (Sternson, 2013). Such systems must synthesize two crucial processes: energy sensing (to determine the resource needs of the body) and appropriate output behaviors that are organized by the brain (to resolve bodily need). For example, resource deficits such as fasting or dehydration increase the motivation to find and ingest food and water, respectively. Resource excess is also coordinated with an appropriate behavioral response: stomach fullness or increased energy reserves (e.g., body fat) cue the cessation of feeding while also promoting physical activity and fat burning to resolve energy excess (Nogueiras et al., 2008; Myers et al., 2009). At their essence, such physiologic “drive” systems thus match bodily need and behavior to ensure survival. These systems must also be dynamic, since bodily resource needs fluctuate considerably each day (from periods of repletion to deficit and back again), and must continually survey the energy and hydration status of the body to detect and resolve any imbalance. Furthermore, physiologic systems that modulate drive to drink, eat and move inherently regulate energy balance- the caloric intake and energy expended that together determine the weight of the organism. Extreme deficits in energy intake impair survival, while excesses in energy can promote metabolic disease and co-morbidities. Thus, survival and energy balance are irrevocably linked, and rely on constant, dynamic communication between the periphery and the brain.

How then does the body convey messages that can be “read” by the brain, and how does the brain interpret these into behaviors to correct energy imbalance? A major step forward in understanding this process was the discovery of circulating hormones that communicate energy status from the periphery to the brain, and how their absence promotes disease. Insulin was the first hormonal body-to-brain regulator to be characterized in control of energy balance (Chen et al., 1975). Insulin is secreted by pancreatic β-cells and is transported from plasma into the brain (Schwartz et al., 1991), where it acts to suppress feeding, hepatic glucose production and to promote weight loss (Woods et al., 1979; Obici et al., 2002). Loss of insulin signaling in the brain, however, promotes overeating, insulin resistance and obesity (Bruning et al., 2000). Another important hormone in body-to-brain signaling is leptin, which is produced in adipose tissue and acts via neurons in the brain that express the long form of the leptin receptor (LepRb) to suppress feeding and promote energy expenditure (Halaas et al., 1995; Pelleymounter et al., 1995; Chua et al., 1996; Cohen et al., 2001). Loss of either peripheral leptin production or central LepRb expression promotes overeating, decreases energy output and leads to severe obesity in rodents (Halaas et al., 1995; Pelleymounter et al., 1995; Chua et al., 1996) and humans (Montague et al., 1997; Farooqi et al., 1999), revealing the crucial role of this periphery/brain regulatory system. The hormone ghrelin also mediates powerful control of energy balance via regulation in the brain. Ghrelin is produced by the stomach during periods of energy deficit and acts via brain neurons expressing the growth hormone secretagogue receptor (GHSR) to stimulate feeding (Nakazato et al., 2001; Zigman et al., 2005). Increased ghrelin action via GHSR promotes over-feeding and potentiates weight gain (Tschop et al., 2000). These examples demonstrate that peripheral cues access the brain to either promote or inhibit feeding behaviors, and thus regulate energy balance.

Normal energy balance relies on the appropriate synergism of peripheral cues and behavior, but uncoupling these deranges energy balance. Indeed, individuals with anorexia nervosa self-restrict their feeding despite having intact cues signaling energy need (Kaye et al., 2013). Similarly, tastiness can trump satiation: few among us are invulnerable to the attractive sight and smell of a dessert, despite having just consumed an ample meal and being energy replete. As such, normal weight and obese individuals may over consume palatable, calorie-dense foods despite the presence of energy excess signals that should inhibit intake (Berthoud, 2012). Thus, eating disorders or obesity occur when the *need to eat* *no longer matches the* *desire to eat* (Berridge et al., 2010), incurring serious health tolls including increased mortality. Yet despite the increasing severity of anorexia in youth (Smink et al., 2012) and the obesity pandemic (Swinburn et al., 2011; Flegal et al., 2012), there remain limited pharmacologic strategies to treat energy imbalance (Bailey et al., 2014; Bray and Ryan, 2014). Modifying diet and exercise remains the gold standard treatment for disordered energy balance, but these lifestyle changes are difficult to maintain long term, yield modest improvements in body weight and prove largely ineffective at improving functional outcomes and life expectancy (Hart et al., 2013; Look et al., 2013; Jensen et al., 2014). Surgical interventions such as gastric banding or gastric bypass are effective in promoting weight loss in obese individuals, but these procedures are highly invasive and many individuals regain weight in subsequent years (Meguid et al., 2008; Dayyeh et al., 2010). It is therefore imperative to identify strategies to restore normal energy balance function to treat the millions of individuals suffering from obesity and eating disorders. Identifying the brain mechanisms that coordinate energy cues and appropriate behavioral response will suggest tractable pharmacological pathways to treat feeding and energy balance disorders.

While many areas of the brain contribute importantly to the regulation of feeding and metabolism, this review will focus on the role of the lateral hypothalamic area (LHA) in controlling energy balance for three reasons: (1) The LHA modifies intake of natural and pharmacologic rewards and physical activity, and such function via the LHA is required for survival, (2) The LHA receives circulating energy balance cues and projects to brain regions that regulate motivated behaviors, (3) Distinct neuronal populations within the LHA are “tuned” to specific energy cues (such as ghrelin or leptin) and induce cue-appropriate behavioral responses. Thus, understanding the precise neurochemistry, connectivity and function of the LHA neuronal subpopulations will suggest mechanisms by which to suppress or enhance feeding, drinking and energy expenditure as required to restore energy balance. Modifying action via the LHA therefore has potential to improve a spectrum of health problems.

## The lateral hypothalamic area (LHA) is a crucial regulator of energy balance

The hypothalamus as a whole has long been recognized to modulate body weight, water balance, body temperature and the sympathetic nervous system (Ranson, 1937). Hetherington and Ranson were the first to imply that each sub-region of the hypothalamus controls specific facets of energy balance, demonstrating that selective lesion of the ventromedial nucleus of the hypothalamus (VMH) caused profound overeating and obesity. The VMH was hence deemed an essential “satiety center” of the brain (Hetherington and Ranson, 1939, 1940) and inspired many labs to study “hypothalamic obesity” caused by VMH lesions. It was in this context that Bal K. Anand (while working at Yale with Brobeck) was using stereotaxic techniques to lesion the VMH of rats and, by his account, “…was much disconcerted to find that my rats immediately after such lesions completely stopped eating and would die of starvation”. This phenotype was completely opposite of the hyperphagia and obesity expected due to lesion of the VMH (Anand, 1980). As it turned out, Anand and Brobeck had made a (fortuitous) targeting error, missing the VMH, but instead ablating the LHA in their experimental rats. The resulting LHA-lesioned rats had the *ability* to move, eat and drink, but lost all *motivation* to do so: as a result they all died of self-inflicted starvation and dehydration (Anand and Brobeck, 1951a,b; Morrison et al., 1958). By contrast, electrical stimulation of the LHA promotes feeding and drinking behaviors, as well as increasing physical activity (Delgado and Anand, 1953; Mogenson and Morgan, 1967; Mogenson and Stevenson, 1967). Collectively, these seminal loss and gain of function experiments led to the initial designation of the LHA as a “feeding center” that acts in opposition to the VMH “satiety center” (Hoebel and Teitelbaum, 1962; Hoebel, 1965), and Eliot Stellar summarized these concepts into the “dual center hypothesis” of feeding regulation (Stellar, 1954). Subsequent work, however, has revealed a more complex role for the LHA in control of feeding, as well as of drinking, physical activity, alertness/arousal and coordination of sensory stimuli with appropriate output behaviors (Levitt and Teitelbaum, 1975). Thus, the LHA is not just a “feeding center” and must be considered in terms of how it coordinates a spectrum of ingestive and arousal behaviors relevant to energy balance.

The fact that LHA-lesioned animals imminently died of starvation and dehydration complicated their use to determine *how* the LHA promoted feeding, drinking and other behaviors. Teitelbaum and Stellar found that rats with LHA lesions could only be kept alive via force-feeding them liquid nutrients three times per day (Teitelbaum and Stellar, 1954). This regimen was a serious toll for Teitelbaum (the last daily treatment was at 2:00 AM!) and he grew desperate for a way to induce the animals to feed themselves. He recalled another time he’d had to stay up till the wee hours dealing with rats, while performing husbandry of a rat colony during his assistantship:

“…I used to stop, munch chocolate bars, and offer the rats some. I soon discovered that shortly before my break, many rats were lined up at the front of each cage, all waiting for their treat. Later, I remembered this when trying to tempt aphagics to eat. Nevertheless, it was a thrill to see a rat, being kept alive by tube-feeding, refusing food and water for 2 months postoperatively, suddenly gobble up bits of chocolate.” (Teitelbaum, 1979)

Thus, Teitelbaum found that LHA-lesioned rats eschewed normal foods, but could be coaxed to eat sufficient calories in the form of palatable substances (i.e., evaporated milk, cookies, milk chocolate but not bittersweet chocolate) to permit their survival (Teitelbaum and Epstein, 1962). Eventually, the lesioned rats overcome their aphagia, resume normal feeding and regain weight. Importantly, this discovery confirmed that loss of LHA function didn’t negate the *ability* to feed, but blunted the *motivation* to feed, even when food is desperately needed for survival. Further, it identified the LHA as being important for feeding drive, though it is not the sole mediator of motivated ingestion; other neuronal systems exert some (presumably lesser) drive that can, in time, be sufficient to mediate survival (Teitelbaum and Epstein, 1962). Intriguingly, drinking drive remains particularly impaired in LHA-lesioned animals even after their “recovery”. These seemingly normal rats do not coordinate physiologic perturbations (e.g., high salt-intake/dehydration, hunger, altered food valuation) with appropriately paired drinking or feeding behavior (Teitelbaum et al., 1969; Marshall and Teitelbaum, 1973; Levitt and Teitelbaum, 1975). Close observation revealed that drinking is strictly time-locked with feeding bouts in these rats, “…as if the animal were drinking not to quench its thirst but simply to wet its mouth…perhaps just as a means to wet food and make it swallow-able” (Teitelbaum et al., 1969). These data, in sum, revealed that the LHA is crucial for *pairing* physiologic need, as conveyed by cues such as dehydration, hunger, etc., with ingestive behavior.

Anatomists challenged the notion that the “cell-poor” LHA could itself regulate motivated behaviors, arguing that it was actually due to lesion or stimulation of the diffuse fiber systems passing through the LHA. Indeed, coursing through the LHA are nigro-striatal dopamine (DA) fibers as well as axons of passage within the medial forebrain bundle (mfb), each of which terminate in brain centers associated with reward and motivation (Ungerstedt, 1971). These tracts regulate motivation, and disruption of the mfb or DA-containing neurons blunts feeding, drinking and movement behavior, similar to LHA lesions (Morgane, 1961; Ungerstedt, 1971; Marshall and Teitelbaum, 1973; Szczypka et al., 1999, 2000). Two crucial findings, however, solidified a specific role for LHA neurons in regulating motivation relevant to energy balance. First, stimulation of the LHA still induces motivated feeding even in rats with a severed mfb (Morgane, 1961). Secondly, treatment with neurotoxins that selectively ablate LHA cell bodies, but spare axons passing through the LHA, results in aphagia and adipsia similar to the original lesions that disrupted both cells and fibers (Grossman et al., 1978; Dunnett et al., 1985). Thus, these data confirmed that neurons within the LHA directly modulate motivated ingestion behaviors. It was subsequently determined that LHA neurons are anatomically linked with neural systems that regulate reward and goal-directed behaviors, including direct projections onto midbrain DA neurons that release DA into the forebrain (Hernandez and Hoebel, 1988). Taken as a whole, the classical lesion, stimulation and anatomical studies established the LHA as a powerful coordinator of the drive to eat, drink and move. Such methodologies, however, could not define how LHA neurons coordinate specific status cues from the body (e.g., need for water vs. food) with appropriate output behaviors.

## Connectivity and neuronal diversity in the LHA: implications for energy balance

**Figure 1:**
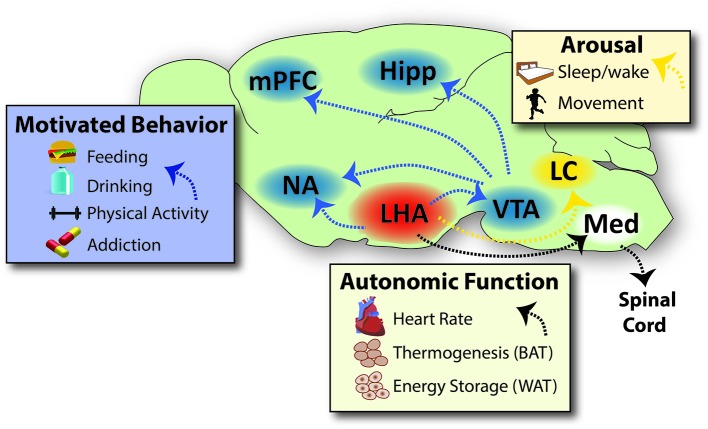
**Projections Via Which the LHA Coordinates Behaviors that Impact Energy Balance**. The LHA projects densely into brain regions that modulate motivated behaviors, such as feeding, drinking, physical activity and addiction to drugs of abuse. The LHA engages the motivational circuit via direct projections into the forebrain (NA) and to the midbrain (VTA), which in turn projects to reward areas of the brain such as the NA, mPFC and Hipp. The LHA also sends dense projections to hindbrain sites that regulate arousal/sleep and movement/vigilance, such as the LC and other sites not shown for sake of clarity. LHA projections onto neurons within the medulla in turn project via the spinal cord to engage the autonomic nervous system, thereby modulating heart rate, respiration, brown adipose tissue/thermogenesis and white adipose tissue to modulate energy storage. Specific neuronal subsets of the LHA engage some or all of these sites to coordinate energy cues and behavioral output that impacts energy balance, as reviewed in the text. Note that not all projections from the LHA are represented on this schematic, such as projections to the prefrontal cortex and hippocampus; though these likely have important roles in regulating physiology, they are beyond the scope of this review and thus have not been represented here. Abbreviations: LHA = lateral hypothalamic area, NA = nucleus accumbens, VTA = ventral tegmental area, mPFC = medial prefrontal cortex, Hipp = hippocampus, LC = locus coeruleus, Med = medulla, BAT = brown adipose tissue, WAT = white adipose tissue. Sagital brain outline adapted from Paxinos and Franklin (2001).

The strikingly different phenotypes produced by lesion of the LHA (aphagia, adipsia, weight loss) or periventricular hypothalamic regions (hyperphagia, obesity) suggests that these regions differ in neurochemistry and/or their anatomical engagement of brain systems that regulate behavior. Hypothalamic nuclei such as the VMH, arcuate nucleus (ARC), dorsomedial hypothalamus (DMH) and paraventricular hypothalamic nucleus (PVN) are compact, cell-dense and have well-defined projection targets throughout the brain. The LHA, by contrast, encompasses a large swath of tissue over the entire rostral-caudal extent of the hypothalamus. The sheer expanse of the LHA, coupled with the fact that it lacks obvious cellular architecture, complicated anatomical and functional studies. The LHA lies lateral to the DMH and PVN, and the area just above and surrounding the fornix is referred to as the perifornical area of the LHA. Pioneering work by the Swanson group utilized these anatomical landmarks in combination with neuronal tract tracing methods to determine the precise connectivity of LHA subregions with the rest of the brain (Goto et al., 2005; Hahn and Swanson, 2010). While these studies characterized some subregion-specific projection targets, as a whole they demonstrate that the LHA projects broadly throughout the forebrain, midbrain and hindbrain regions, each of which is implicated in distinct facets of physiologic control; the LHA projections discussed in this review are shown in Figure 1. The lack of a unified output region, however, suggests that LHA-mediated regulation of behavior and energy balance is complex and not homogenous in nature.

The next leap in understanding the LHA’s role in energy balance was the discovery of its hetero-cellularity, and the resulting concept that *specific populations* of LHA neurons coordinate discrete energy cues and behavioral response. The full extent of LHA neurons are yet to be characterized, but three substantial populations of neurons have been described and can be defined by their expression of a specific neuropeptide: neurons containing melanin concentrating hormone (MCH), the orexins/hypocretins (OX) or neurotensin (Nts). Intriguingly, these neuronal populations are molecularly and spatially distinct, suggesting that each population may also differ in connectivity and functional output (Elias et al., 1998; Swanson et al., 2005; Leinninger et al., 2011).

The development of molecular techniques that enable site-specific manipulation of genetically-distinct neuronal populations has allowed the field to probe the roles of MCH, OX and Nts neurons, and suggests that each of these populations have roles in regulating energy balance. While there are also smaller populations of neurons within the LHA expressing other neuropeptides and neurotransmitters, we will focus on the emerging and distinct roles of MCH, OX and Nts neurons in coordinating peripheral energy cues and behaviors, and their respective contributions to energy balance. In particular each neuronal population will be considered for its role(s) in regulation of feeding, drinking and energy expenditure. The amount of energy expended (and thus weight), is influenced by the amount of physical activity, sleeping/arousal and thermogenic outflow, so each of these facets of energy expenditure will be discussed.

## Melanin concentrating hormone (MCH) neurons

### Discovery and characterization of MCH signaling

Melanin concentrating hormone (MCH) is a 19 amino acid cyclic neuropeptide that was first documented in the pituitary of teleost fish, enabling them to change skin color and blend into their environment (Kawauchi et al., 1983; Oshima et al., 1986). Soon after MCH was identified in the brains of rats (Zamir et al., 1986) and humans (Mouri et al., 1993), where it is primarily found within neuronal cell bodies of the LHA and a few neurons in the zona incerta (Bittencourt et al., 1992). Most often these are solely referred to as MCH neurons, but they also contain the classical (fast) neurotransmitters GABA or glutamate via which they can inhibit or excite postsynaptic cells (Elias et al., 2001; Harthoorn et al., 2005; Jego et al., 2013). Additional sub-populations of MCH neurons can be differentiated by their co-expression of nesfatin (Foo et al., 2008) or the neuropeptide cocaine-amphetamine-regulated transcript (CART; Vrang et al., 1999; Elias et al., 2001; Brischoux et al., 2002; Cvetkovic et al., 2004). CART co-expression signifies a distinct MCH population that projects to forebrain sites involved in behavior modulation, while non-CART expressing MCH neurons preferentially project to caudal hindbrain and spinal cord (Brischoux et al., 2002; Cvetkovic et al., 2004).

MCH acts via neurons expressing the G-protein coupled MCH Receptor-1 (MCHR-1), which can be coupled to either G_*i/o*_ or G_*q*_ proteins (Chambers et al., 1999; Lembo et al., 1999; Saito et al., 1999; Hawes et al., 2000; Pissios et al., 2003). Human, primates, cats and dogs (but not rodents) also express a G_*q*_-coupled MCH Receptor-2 that activates target neurons and may exert opposite actions to MCHR-1 (An et al., 2001; Rodriguez et al., 2001; Chee et al., 2014). MCHR-1 is highly expressed within neurons of the cerebral cortex, olfactory tubercle, limbic structures (hippocampus, septum, nucleus of the diagonal band, bed nucleus of the stria terminalis, amygdala) forebrain (caudate-putamen, nucleus accumbens (NA) core and shell) and the ARC (Chee et al., 2013). MCH neurons also project to areas implicated in regulating feeding, such as the parabrachial nucleus (Touzani et al., 1993) and PVN (Fekete et al., 2004), but project sparsely to regions that regulate arousal, such as the dorsal raphe (DR), ventrolateral periaqueductal gray (VLPAG), locus coeruleus (LC) and preoptic area (Chambers et al., 1999; Saito et al., 1999; Borowsky et al., 2002; Chee et al., 2013; Yoon and Lee, 2013). Though the LHA as a whole densely projects into the DA-enriched ventral tegmental area (VTA) and regulates DA-mediated ingestive and locomotor behaviors (Kenny, 2011), MCH neurons do not regulate the VTA (Chee et al., 2013). Rather, MCH neurons engage the DA system via projections to the NA, where MCHR-1 is expressed on dopamine receptor-1 (D1R) and dopamine receptor-2 (D2R)-expressing medium spiny neurons (Pissios et al., 2008). MCH acts via a G_*i/o*_-dependent signaling mechanism to increase K^+^ current into medium spiny neurons and reduce their excitability (Sears et al., 2010). Given that inhibition of NA neurons promotes motivated behaviors for natural rewards (Taha and Fields, 2006), MCH signaling via G_*i/o*_-coupled MCHR-1 may inhibit NA neurons to promote ingestion. Consistent with this model, rodents lacking either MCH or MCHR-1 exhibit reduced DA-mediated motivated behaviors (Pissios et al., 2008; Tyhon et al., 2008). At least one report, however, suggests that MCH and DA act synergistically to enhance activation of NA shell neurons (Chung et al., 2009). These conflicting reports of MCH either inhibiting or promoting the activation of NA neurons could be due to different G_*i/o*_ or G_*q*_ coupled signaling via the receptor though this has yet to be thoroughly explored. MCH neurons also project to hindbrain regions including the nucleus of the solitary tract, dorsal motor nucleus of the vagus and ventral medulla sympathetic premotor areas (Bittencourt et al., 1992; Zheng et al., 2005b). Hindbrain neurons regulated by MCH project via the spinal cord or nerves to exert sympathetic tone within the gastrointestinal, cardiovascular, respiratory and thermoregulatory systems, including regulation of white and brown adipose tissue (BAT) to modify energy expenditure (Oldfield et al., 2002, 2007; Zheng et al., 2005b; Stanley et al., 2010; Adler et al., 2012).

### MCH and feeding

Central injection of MCH into the brain increases feeding in rodents and promotes obesity thus it is considered an orexigenic neuropeptide (Qu et al., 1996; Rossi et al., 1997; Glick et al., 2009). Strongly orexigenic neuropeptides such as neuropeptide Y stimulate appetitive (meal frequency) and consummatory (meal size) aspects of feeding behavior via the forebrain and hindbrain (Baird et al., 2006a). By contrast, MCH only amplifies consumption (size/amount) of normally accepted substances (water, sucrose, but not bitter quinine), and this aspect of MCH action is selectively controlled via the forebrain (Baird et al., 2006b, 2008). MCH treatment, however, does not *preferentially* stimulate intake of palatable food or sucrose, suggesting that MCH regulates general consumption behavior, but not necessarily hedonic aspects of feeding (Clegg et al., 2002; Sakamaki et al., 2005). Consistent with this, MCH expression is increased in hungry animals, including fasted or hyperphagic leptin-deficient mice (*ob/ob)*, compared to normal controls (Qu et al., 1996). Similarly, mice that genetically overexpress MCH are hyperphagic and gain weight (Ludwig et al., 2001). In contrast, genetically engineered mice that lack MCH eat less, are lean and exhibit improved metabolic profiles throughout aging (Shimada et al., 1998; Jeon et al., 2006; Willie et al., 2008). Mice lacking MCHR-1 are also lean with less body fat than controls, but this is primarily due to their increased locomotor activity and energy expenditure. While one might expect decreased feeding in MCHR-1 deficient mice (due to loss of orexigenic MCH action), they actually display mild overeating. In this case the modest hyperphagia may be required to support their increased energy expenditure, but at any rate, is not sufficient to produce obesity (Chen et al., 2002; Marsh et al., 2002). Blocking acute action of MCHR-1 via selective antagonists, however, does suppress feeding, meal size and weight gain in normal weight and obese rodents (Borowsky et al., 2002; Kowalski et al., 2004, 2006). These findings have accordingly spurred interest in development of brain-permeable MCHR-1 antagonists to reduce food intake and promote weight loss.

MCH action in the NA shell is particularly important for increasing the intake of naturally ingested substances. Selective administration of MCH in the NA increases feeding, but delivery of an MCHR-1 antagonist in this region inhibits food intake (Georgescu et al., 2005). Genetic deletion or pharmacologic antagonism of MCHR-1 also blunts cue-induced responding for food, suggesting a deficit in learning processes that drive motivated feeding (Sherwood et al., 2012). MCH neurons sense nutrient status and accordingly promote the motivation to feed in order to maintain euglycemia (Kong et al., 2010). Indeed, activation of MCH neurons promotes intake of sweetened liquids along with DA output into NA (Domingos et al., 2013). Thus, MCH neuronal signaling via MCHR-1 in the NA is sufficient to coordinate energy need and feeding, and may be a tractable pathway to modulate feeding in energy balance disorders.

### MCH and drinking

Central administration of MCH increases water intake in the presence or absence of food (Clegg et al., 2003; Sakamaki et al., 2005). Woods and colleagues demonstrated, however, that MCH does not specifically promote water intake, and also increases ingestion of ethanol, sucrose solution and food (Duncan et al., 2005). Therefore, MCH is likely a general inducer of intake behavior (eating and drinking). MCH may influence the desire to drink in order to wet the mouth or via a DA-mediated reward mechanism, but does not seem to have a specific role in controlling thirst *per se* (Watts and Sanchez-Watts, 2007).

### MCH and energy expenditure

#### MCH control of physical activity and sympathetic outflow

Based upon the hypophagia of mice lacking MCH, it was hypothesized that MCH deletion could curb feeding to promote weight loss in obesity. The Maratos-Flier group tested this by genetically deleting MCH in mice that are deficient for leptin, and hence are hyperphagic and obese. The resulting double MCH/leptin knock-out mice were leaner than leptin-depleted controls, but did not exhibit any blunting of feeding. Instead, the reduced adiposity of MCH/leptin knock-out mice was due to their increased energy expenditure (Segal-Lieberman et al., 2003). Indeed, MCH neurons act via polysynaptic connections to the hindbrain and spinal cord to regulate BAT, the vital tissue for promoting thermogenesis and basal metabolic rate (Oldfield et al., 2007). MCH neurons presumably inhibit thermogenic energy expenditure via this pathway (Bittencourt and Elias, 1998; Zheng et al., 2005b). By contrast, blockade of MCH signaling increases BAT mass and thermogenesis and reduces body weight (Pereira-Da-Silva et al., 2003). Genetic deletion of MCHR-1 in mice also promotes hyperactivity and increased metabolic rate, (Marsh et al., 2002; Smith et al., 2005) though some part of this is mediated via changes in the NA (Smith et al., 2005). Together these data suggest that the combined increase of thermogenesis and physical activity supports weight loss and leanness despite the hyperphagia of these animals. Interestingly, ablation of MCH neurons in adult obese mice does not decrease their feeding or body weight (Wu et al., 2012), suggesting that developmental disruption of MCH neurons is essential for modifying energy balance.

MCH is also implicated in regulating anxiety and anxiety-related measures of energy expenditure. MCH may promote anxiety in humans since MCH levels are lowest during (presumably positive) social interactions, and this has inspired interest in MCHR-1 blockade for treating anxiety disorders (Blouin et al., 2013). Rodents treated with systemic MCHR-1 antagonists or that have a genetic knock-out of MCHR-1 exhibit decreased anxiety behavior, including improving performance in forced-swim and social interaction tests (Borowsky et al., 2002; Georgescu et al., 2005; Smith et al., 2006). Such assays inherently measure locomotor outcomes, so the improved performance in these anxiety tests may reflect the general increase in locomotor activity resulting from MCHR-1 blockade. Other reports using MCHR-1 antagonists, however, have not confirmed a role for MCH in anxiety suggesting that more work is needed to understand the complex role of MCH in this system (Basso et al., 2006).

#### MCH and arousal/sleep

Recently central MCH action has also been implicated in promoting sleeping, and blockade of sleep could promote the energy expenditure and anxiety effects observed with disrupted MCH signaling. Central MCH promotes paradoxical (REM) sleep, while MCHR-1 antagonists reduce it (Verret et al., 2003; Ahnaou et al., 2008; Lagos et al., 2009; Clement et al., 2012). Optogenetic activation of MCH neurons also promotes paradoxical sleep (Jego et al., 2013; Konadhode et al., 2013; Tsunematsu et al., 2014). In humans MCH levels are maximal during sleeping (Blouin et al., 2013), and MCH neurons selectively fire during sleep (Hassani et al., 2009; Sapin et al., 2010; Clement et al., 2012). Thus, MCH neurons may coordinate energy savings during periods of sleep, including diminishing heart rate and motor action/tone via the sympathetic system. Interestingly, MCH action itself may not be as important as GABA released from these neurons in promoting sleep (Jego et al., 2013). By contrast, histamine-producing neurons of the tuberomammillary nucleus regulate wakefulness, in part via inhibiting MCH neurons, identifying an important on/off sleep circuit (Parks et al., 2014).

## Orexin/hypocretin (OX) neurons

### Discovery and characterization of OX signaling

In 1998 two groups reported the discovery of two neuropeptides produced from the same gene product: one group dubbed them the hypocretins (de Lecea et al., 1998) and the other referred to them as orexins (Sakurai et al., 1998). We will utilize the orexin (OX) designation due to its simple abbreviation. The prepro-OX gene product is proteolytically cleaved into two similar peptide products, OX-A and OX-B, whose expression is restricted to neuronal cell bodies in the LHA and to a lesser extent the DMH (Peyron et al., 1998). OX action is transduced via two G protein coupled receptors, orexin receptor-1 (OXR-1) and orexin receptor-2 (OXR-2; Sakurai et al., 1998; Zhu et al., 2003; Holmqvist et al., 2005). OXR-1 preferentially binds OX-A and couples to the G_*q*_ subclass of G proteins (Sakurai et al., 1998). OXR-2 binds OX-A and -B with equal affinities and can couple to G_*q*_, G_*s*_ or G_*i/o*_ proteins (Martin et al., 2002; Tang et al., 2008; Ramanjaneya et al., 2009). Strikingly, OX neurons project broadly throughout the brain (Peyron et al., 1998) and virtually every brain region contains at least one of the two OX receptors, suggesting that central OX action controls a wide array of functions (Trivedi et al., 1998). For example, OX neurons directly innervate neurons located in reward regions of the brain (VTA, NA [dopaminergic neurons]), as well as a host of regions and signals that regulate arousal (LC [noradrenergic neurons], DR [serotonergic neurons], periaqueductal gray, lateral dorsal tegmental nucleus, peduncolopontine nucleus, diagonal band, septal nucleus [cholinergic and other neurons], tuberomammillary nucleus [histaminergic neurons] and paraventricular thalamus [multiple populations of neuropeptide-expressing neurons]) (Peyron et al., 1998; Horvath et al., 1999a). OX neurons also project into regions that regulate learning (amygdala, hippocampus), within hypothalamic nuclei that contribute to energy balance such as the ARC, VMH, DMH, PVN, and LHA itself, and to caudal hindbrain regions that regulate autonomic control (reticular formation areas, solitary nucleus, spinal cord) as well as many others (Peyron et al., 1998; Chemelli et al., 1999; Date et al., 1999; van den Pol, 1999). Via projections to the raphe pallidus (Lee et al., 2013) and other caudal hindbrain sites OX neurons poly-synaptically regulate BAT (Oldfield et al., 2002), white adipose tissue (WAT), liver (Stanley et al., 2010; Adler et al., 2012), the pancreas (Wu et al., 2004) and the gastrointestinal system (Grabauskas and Moises, 2003). The ability of OX neurons to engage multiple neural systems indicates a powerful role for OX neurons to promote simultaneous, multifaceted physiological response.

Soon after the discovery of OX followed high impact reports of its roles in sleep and energy balance (Peyron et al., 2000; Thannickal et al., 2000; Hara et al., 2001; Yamanaka et al., 2003). Subsequently, researchers have begun to approach the OX system via studying selective OX-mediated circuits, to determine the contributions of OX action via specific regions of the brain. Furthermore, several lines of evidence suggest that there are spatially and electrophysiologically-distinct subsets of OX neurons, but it remains unclear if subsets preferentially project to distinct brain regions or how they specifically contribute to OX-mediated behavior (Swanson et al., 2005; Harris and Aston-Jones, 2006; Williams et al., 2008; Schöne et al., 2011). Neurons containing OX are generally defined as “OX neurons”, but they also co-express insulin-like growth factor binding protein-3 (Honda et al., 2009), dynorphin (Chou et al., 2001), the secreted neuronal pentraxin NARP/Nptx2 (Reti et al., 2002) and glutamate (Rosin et al., 2003; Torrealba et al., 2003). These appear to be common to all OX neurons, and thus have not been useful in discriminating between functional subsets. OX neurons release dynorphin and glutamate from their synaptic terminals throughout the brain, including at synapses onto themselves (Li et al., 2002). The glutamatergic output from OX neurons and OX-mediated suppression of G-protein coupled inward rectifier (GIRK) channel activity promotes neuronal excitation (Hoang et al., 2003). OX neurons also autoregulate via their expression of OXR-2, such that autaptic OX signaling maintains sustained activation of OX neurons (Yamanaka et al., 2010). By contrast, dynorphin released from OX neurons onto themselves, or likely onto other glutamatergic inputs, may dampen the activation of OX neurons (Li and van den Pol, 2006). Considering the broad projections of OX neurons throughout the brain, it is tempting to speculate that their release of multiple transmitters enables OX neurons to differentially control targets that express a specific receptor repertoire (e.g., glutamate receptors but not OXR-1 or OXR-2; Li and van den Pol, 2006).

### OX and feeding

Central OX administration acutely promotes feeding (Sakurai et al., 1998; Ida et al., 1999; Zheng et al., 2005a) though more modestly compared to other orexigenic neuropeptides (Edwards et al., 1999). Pharmacological OX potentiates feeding during the day (when rodents are normally sleeping) but not during dark cycle (when rodents are awake and do most of their feeding) (Haynes et al., 1999; Yamanaka et al., 1999; McGregor et al., 2011). These data collectively support the argument that OX is not a stimulator of food intake *per se*, but that it increases arousal and consequently increases feeding. Simply put, awake animals will eat. Thus, activating OX neurons or administering OX during their sleeping period wakes the animal, and the animals spend some of their awake time eating (España et al., 2002; Inutsuka et al., 2014). It logically follows that suppressing OX action when animals are normally awake and eating should reduce their feeding; indeed central OXR antagonists suppress feeding during the dark phase (Haynes et al., 2000). Loss of OX neurons results in either reduced or unchanged food intake, but also increased sleep, and it’s likely that the reduced waking time inherently limits the time for ingestion (Zhang et al., 2007; Inutsuka et al., 2014; Tabuchi et al., 2014).

A growing body of work implicates OX neurons as sensors of when and if an animal needs to eat. For example, survival in the wild depends on recognizing cues that signal food availability (e.g., light, sounds) and to promote locomotor activities to obtain the food. OX neurons are activated in anticipation of feeding and couple arousal and feeding behaviors (Akiyama et al., 2004; Mieda et al., 2004). Accordingly, OX neurons directly sense energy status: fasting (forced hypoglycemia) increases OX expression (Cai et al., 1999) and activation of OX neurons, while physiologic increases in glucose that occur with re-feeding or satiation selectively inhibit OX neurons (Burdakov et al., 2005; Williams et al., 2008). Non-essential amino acids also activate OX neurons, even in high glucose conditions that normally suppress OX activation (Karnani et al., 2011). Physiologically, amino acid biased sensing may exist to detect inappropriate increases in nonessential vs. essential amino acids, such as during starvation-induced muscle breakdown when amino acids are released into the circulation. In this case, amino acid-mediated activation of OX neurons could promote food seeking and intake behavior to resolve the energy deficit (Karnani et al., 2011). In general calorie-restriction activates OX neurons (Diano et al., 2003) and OX signaling is essential to increase locomotor survival behaviors, including enabling food seeking (Lutter et al., 2008).

OX neurons regulate intake of natural and drug rewards, at least in part, via direct projections onto VTA DA neurons (Fadel and Deutch, 2002; Korotkova et al., 2003). OX neurons are activated during cue-induced feeding (Petrovich et al., 2012; Cason and Aston-Jones, 2013b), and in turn they activate VTA DA neurons and promote DA release into the NA and prefrontal cortex (Vittoz and Berridge, 2006; España et al., 2010, 2011). OX regulation via this mesolimbic circuit promotes ingestion of highly salient substances (e.g., high fat diet, drugs of abuse) but not of comparatively bland chow or aversive substances (Harris et al., 2005; Thorpe and Kotz, 2005; Borgland et al., 2009; España et al., 2011; Richardson and Aston-Jones, 2012; Mahler et al., 2013). Furthermore, OX specifically promotes motivated response (work) for palatable foods that is attenuated by OXR antagonists (Choi et al., 2010; Cason and Aston-Jones, 2013a,b). These reward responses may be due to OX-induced synaptic plasticity, since OX potentiates NMDA receptor-mediated excitatory postsynaptic potentials at VTA DA neurons and promotes behavioral sensitization (Borgland et al., 2006). Both OX and glutamate release, however, are required for long-term potentiation of DA signaling that underlies cue-induced reinstatement (seeking) of rewards. Given that OX and glutamate are released from the same neuron, it is proposed that OX potentiates the glutamate-mediated long-term modifications that are known to underlie addiction to drugs and natural rewards (Mahler et al., 2013). By contrast, a decrease in the ratio of OX and dynorohin signaling suppresses reward response (Muschamp et al., 2014). Similarly, genetic or pharmacologic disruption of OX signaling (but presumably leaving other releasable transmitters in OX neurons intact) inhibits DA release to the NA and operant responding for drugs, food or sucrose (Abizaid et al., 2006; España et al., 2010; Sharf et al., 2010; Smith and Aston-Jones, 2012; Srinivasan et al., 2012). Based on these data it is tempting to speculate that distinct transmitters released from OX neurons have different roles in conveying resource need and regulating ingestive behavior, but more work is needed to address this hypothesis.

Circulating factors modulate the activity of OX neurons to coordinate bodily energy status with the appropriate feeding behavior. GLP1, for example, activates OX neurons (Acuna-Goycolea and van den Pol, 2004), as does ghrelin acting via growth-secretagogue hormone receptors (Lawrence et al., 2002; Olszewski et al., 2003; Toshinai et al., 2003). Ghrelin robustly increases feeding via distinct neuronal populations in the brain including OX neurons (Toshinai et al., 2006). Ghrelin also promotes phasic activation of VTA DA neurons, but OXR antagonists blunt these effects and palatable food intake, indicating a crucial involvement of OX neurons in bridging ghrelin and DA signaling (Perello et al., 2010; Cone et al., 2014). Direct ghrelin sensing via OX neurons can thus coordinate signals of diminished energy reserves to the VTA to promote DA-mediated food seeking and feeding (Sheng et al., 2014). Similarly, decreased extracellular glucose levels (as occurs during fasting), also increase the activation of most OX neurons, presumably to promote OX-mediated arousal and locomotor processes that permit food seeking (Yamanaka et al., 2003; Burdakov et al., 2005). Increases in glucose, as might occur after feeding, initially suppresses the activity of OX neurons (Sheng et al., 2014), but most OX neurons adapt so as to maintain excitability and sustain arousal during energy repletion (Williams et al., 2008).

In contrast, the hormonal signal of energy surfeit, leptin, causes inhibition of OX neurons and attenuates OX-mediated food intake (Zhu et al., 2002). The mechanism by which leptin inhibits OX neurons has been controversial. Reports suggest that OX neurons express LepRb and thus can be directly regulated via leptin; these studies used an antiserum that binds LepRb, but which can also bind the short, non-signaling form of the LepRb (Håkansson et al., 1999; Horvath et al., 1999a). Other groups, using electrophysiogic approaches or knock-in mice that specifically identify LepRb-expressing neurons, have found that OX neurons do not express LepRb and are not directly regulated via leptin (Goforth et al., 2014; Sheng et al., 2014). Instead, separate LepRb-expressing neurons in the LHA on project to OX neurons (Louis et al., 2010; Leinninger et al., 2011). Most of these LepRb neurons contain Neurotensin (Nts) and leptin action via this circuit can inhibit OX neurons (Goforth et al., 2014; Sheng et al., 2014). In normal weight animals the synaptic inputs onto OX neurons are predominantly excitatory, but fasting (e.g., low leptin tone) increases the excitatory inputs onto OX neurons. Leptin treatment attenuates the fasting-induced increase in excitatory tone, presumably restoring (e.g., diminishing) activation of OX neurons to normal levels (Horvath and Gao, 2005). While it is tempting to infer that leptin solely acts to oppose OX neurons, more recent data suggest that leptin and OX act cooperatively to coordinate energy sensing and behavior in the long term. For example, OX neurons receive cannabinoid-receptor type 1 (CB_1_)-expressing synaptic inputs, and in lean mice these CB_1_ terminals are primarily glutamatergic (excitatory). In the obese state the ratio of CB_1_-expressing inputs is predominantly GABAergic (inhibitory), but leptin treatment restores excitatory CB_1_-terminal bias similar to that of lean mice (Cristino et al., 2013). At some level leptin and OX signaling may be synergistic, since disruption of one system also deranges the other. Indeed, loss of leptin or LepRb promotes obesity and decreases *OX* expression compared to normal weight animals (Cai et al., 2000; Yamanaka et al., 2003). Similarly, chronic OX overexpression or treatment with OXR-2 agonists improves leptin sensitivity, and suppresses palatable food intake and weight gain (Funato et al., 2009). Clearly the control of OX neurons by nutrient and hormonal cues is complex and intricately tuned to coordinate energy reserves with appropriate modulation of energy balance.

### OX and drinking

Central OX treatment in rats increases drinking, while water deprivation increases *OX* expression (Kunii et al., 1999). Similarly, pharmacogenetic activation of OX neurons increases water intake (Inutsuka et al., 2014) but genetic ablation of OX neurons decreases drinking (as well as feeding, locomotor activity, and wakefulness) suggesting drive reduction (Zhang et al., 2007; McGregor et al., 2011; Tabuchi et al., 2014). Mice lacking OX also drink less sucrose, although their preference for it is unaffected (Matsuo et al., 2010). Consistent with this, pharmacological antagonism of OXRs reduces all liquid intake (water, ethanol and sucrose), suggesting that the OX system promotes general drinking behavior, regardless of the liquid’s caloric or rewarding value (Anderson et al., 2014). Activation of OX neurons is linked with bodily fluid status, such that OX neurons are inactive during dehydration, but are activated just after drinking/re-hydration occurs (Watts and Sanchez-Watts, 2007). OX neurons may, therefore, enhance the motivation to drink when fluid is available to resolve the water imbalance.

Mechanistically, OX neurons may modulate drinking behavior via projections to, and excitatory regulation of the subfornical organ (Kunii et al., 1999; Ono et al., 2008) as well as projections to the medulla (Zheng et al., 2005a). OX neurons may also modify drinking behavior via projections into the mesolimbic and striatal systems, which are implicated in motivational drinking and drinking secondary to psychiatric dysfunction (psychogenic polydipsia). Polydipsia is a common complication in psychiatric patients, particularly in schizophrenics, that promotes excessive water intake (3–10 L per day) and can lead to water intoxication, seizures, coma and death (Dundas et al., 2007; Hawken et al., 2009; Iftene et al., 2013). Two independent reports identified polymorphisms in OXR-1 that link with psychogenic polydipsia (Meerabux et al., 2005; Fukunaka et al., 2007). It is unclear whether these OXR-1 polymorphisms confer gain or loss of function, though animal studies suggest that increased OX signaling promotes drinking. However, experimental polydipsia induced with dopamine receptor-2 antagonists (D2R) is enhanced via simultaneous antagonism of OXR-1, suggesting that loss of OX signaling could also promote over-drinking (Milella et al., 2010).

### OX and arousal/energy expenditure

#### OX and physical activity

Central OX increases locomotor activity, mainly in the form of grooming and food seeking behaviors (Ida et al., 1999; Rodgers et al., 2001; Kiwaki et al., 2004). OX neurons are maximally activated during exploration, grooming and feeding, (Mileykovskiy et al., 2005), and pharmacogenetic-mediated activation of OX neurons increases locomotor activity (Inutsuka et al., 2014), linking OX action with locomotor drive. Inflammatory challenge, however, reduces OX activation (Becskei et al., 2008; Park et al., 2008) and produces the lethargy characteristic of acute and chronic illness (Gaykema and Goehler, 2009; Grossberg et al., 2011). OX can regulate somatic movement (Zhang et al., 2011) but primarily controls motivated locomotor activity via activation of VTA DA neurons and DA release into the NA (Narita et al., 2006). Inhibitors of DA signaling thus blunt OX-mediated locomotor activity (Kotz et al., 2006). Rodents lacking OX are hypoactive, exhibit less motivated wheel running and their decreased volitional energy expenditure promotes weight gain (Anaclet et al., 2009; Tabuchi et al., 2014). Furthermore, accumulating data suggest that OX expression and/or function is disrupted and may underlie development of movement disorders such as Huntington’s Disease and Parkinson’s Disease (Thannickal et al., 2007; Aziz et al., 2008; Williams et al., 2011; Wienecke et al., 2012).

While lack of OX action can suppress locomotor activity, increased OX signaling can promote stress and panic-induced locomotor activity. OX neurons are activated by the stress related corticotropin-releasing hormone (CRH), which coordinates locomotor response during stressful experiences to facilitate survival, such as the ability to escape predators (Winsky-Sommerer et al., 2004; Xie et al., 2008; Heydendael et al., 2011). Similarly, OX also has a role in panic disorder, which is defined as recurrent anxiety episodes that occur with increased cardio-respiratory action as part of the sympathetic “fight or flight” response. Rodent models of panic disorder exhibit increased activation of OX neurons, and individuals displaying suicidal behavior also have increased central levels of OX. Limiting OX tone mitigates panic-induced elevations in locomotor activity and heart rate similar to antidepressant treatment (Johnson et al., 2010). Chronic stress and sustained activation of the OX system could also promote overconsumption of palatable foods and weight gain (Pankevich et al., 2010). Repeated induction of panic, however, blunts OX mRNA expression likely via negative feedback, and may suggest why individuals subjected to chronic, extreme stress episodes (such as combat veterans exhibiting post traumatic stress disorder) exhibit low levels of central OX: the OX system is tapped out (Strawn et al., 2010). OX action via OXR-1 and OXR-2 exert distinct regulation of neuronal subtypes that function in stress and arousal, thus there is hope that targeted OXR-pharmacotherapy may be useful to treat panic disorder, PTSD and possibly energy balance disorders (Mieda et al., 2011; Scott et al., 2011; Wu et al., 2011).

#### OX and arousal/sleep

The normal function of OX neurons is coordination of environmental cues and arousal. As such, OX neurons are preferentially activated just prior to and during active waking, but are inhibited during sleep (Estabrooke et al., 2001; Eggermann et al., 2003; Lee et al., 2005). Depletion of OX underlies human narcolepsy, which is characterized by increased daytime sleepiness, cataplexy and abrupt transitions from wakefulness to paradoxical sleep (Nishino et al., 2000; Peyron et al., 2000; Thannickal et al., 2000). Narcoleptic individuals also have a higher BMI and increased incidence of metabolic syndrome or type-2 diabetes, likely due to reduced OX-mediated volitional movement and energy expenditure (Honda et al., 1986; Schuld et al., 2000; Nishino et al., 2001; Poli et al., 2009). Clinically, narcoleptic patients exhibit low OX in the CSF, but normal levels in plasma, confirming that the disease is due to central deficit (Dalal et al., 2001). Narcolepsy symptomology is not apparent until there is physical loss of 80–90% of OX neurons, at which point OX and other co-expressed proteins are virtually absent (Crocker et al., 2005). OX neurons are also reduced after traumatic brain injury, which similarly results in chronic daytime sleepiness and reduced locomotor activity (Baumann et al., 2005, 2009; Willie et al., 2012). Additionally, the number of OX neurons diminishes with age, which may underlie aging-related lethargy and sleeping disorders (Kessler et al., 2011). It remains unclear, however, why OX neurons are particularly prone to injury in disease and aging. Yet, the essential requirement for the OX system in mediating arousal has been recapitulated experimentally: loss of OX signaling due to genetic deletion or ablation of OX neurons causes reduced locomotor activity and narcoleptic-like behavior in rodents (Chemelli et al., 1999; Gerashchenko et al., 2001; Hara et al., 2001; Willie et al., 2003; Beuckmann et al., 2004). Restoration of OX expression to mice genetically lacking OX ameliorates their narcolepsy (Liu et al., 2008; Kantor et al., 2013) and the release of OX itself is sufficient to mediate sleep/wake transition (Carter et al., 2009). Importantly, the OX system does not impede sleep *per se*, but is important in mediating the *transitions* between sleeping and waking; mice genetically lacking OX have a low transition threshold between sleeping and waking, which presents as an inability to sustain wakefulness and increased amount of sleeping (Mochizuki et al., 2004; Diniz Behn et al., 2010). Similarly, optogenetic suppression of OX neurons increases sleep (Tsunematsu et al., 2011) but optogenetic activation of OX neurons promotes arousal from sleep (Adamantidis et al., 2007; Rolls et al., 2011). Indeed, narcolepsy, traumatic brain injury and aging-related sleeping disorders are similarly characterized as having inappropriate transitions between waking and sleeping that manifest as exaggerated sleepiness. Thus, restoration and/or enhancement of OX action may be clinically useful in treating narcolepsy, head-trauma and aging-induced sleeping disorders.

Synaptic inputs onto OX neurons modulate their activation state, and the neurochemistry of these inputs suggests potential mechanisms to modify arousal. For example, MCH neurons within the LHA modulate OX neuronal tone, and may be a local system for promoting sleep/wake states, respectively (Rao et al., 2008). Energy status (e.g., fasted/fed) and circadian rhythms remodel the excitatory input to OX neurons and thus coordinate energy and time-appropriate arousal (Horvath and Gao, 2005; Appelbaum et al., 2010). Likewise, the arousal promoting drug modafinil works by increasing excitatory tone onto OX neurons similar to long-term potentiation (Rao et al., 2007). Inhibition of GABAergic or adenosine tone onto OX neurons also promotes their activity and increases wake time (Alam et al., 2005; Rai et al., 2010). OX neurons have GABA_A_ receptors and GABA_B_ receptors (Sergeeva et al., 2005; Xie et al., 2006), via which GABA may inhibit OX neurons and promote the transition to the sleep state (Alam et al., 2005; Matsuki et al., 2009). Indeed, agonists of GABA_A_ receptors such as benzodiazepines and anesthetics inhibit OX neurons and their arousal/vigilance-mediated effects (Sergeeva et al., 2005). GABAergic regulation of OX neurons and OXR antagonists are accordingly of great interest, since these may be useful pharmacologic strategies to treat insomnia (Brisbare-Roch et al., 2007).

OX neurons exert regulation of arousal via projections to specific target regions of the brain, including the LC, the dorsal raphe (DR) the tuberonmamillary nucleus (TMN) and via regulation of LHA MCH neurons. The LC receives dense innervation from OX neurons, and OX increases activation of LC neurons, arousal and locomotor behaviors (Hagan et al., 1999; Horvath et al., 1999b; Bourgin et al., 2000; Walling et al., 2004). OX neurons mediate sleep to wake transition via activation of LC noradrenergic neurons (Chen et al., 2008; Carter et al., 2012), and restoration of OXR-1 signaling in the LC of narcoleptic rodents rescues their ability to sustain wakefulness (Hasegawa et al., 2014). Restoration of OX action in the serotonergic DR, by contrast, suppresses the cataplexy (freezing) of narcoleptic models (Hasegawa et al., 2014). OX also activates histaminergic neurons of the TMN (Bayer et al., 2001; Eriksson et al., 2001; Schone et al., 2012). Histamine mediates at least some portion of OX effects on wakefulness, as OX action is blunted during histamine receptor blockade (Huang et al., 2001). Restoration of OXR-2 signaling selectively in TMN, however, rescues arousal function, suggesting an important OX and histamine-integrated circuit (Mochizuki et al., 2011). Collectively these data suggest that OX action via specialized centers of the brain, and their distinct expression of OXR-1 and/or OXR-2 mediate different aspects of alertness and motor control.

#### OX and sympathetic control

OX neurons are referred to as “command neurons” because they project to brain sites that simultaneously control feeding, locomotor activity (hypothalamus, VTA) and arousal (LC, DR, TMN), as well as to sympathetic response centers that are necessary to support such behaviors (Oldfield et al., 2007). OX increases sympathetic outflow, therefore increasing blood pressure, heart rate, respiration and body temperature in rats, via OX action in caudal medullary sites such as dorsal vagal complex and raphe pallidus (Shirasaka et al., 1999; Wang et al., 2001; Zheng et al., 2005a; Johnson et al., 2012). OX neurons activate sympathetic premotor neurons in the raphe pallidus that accordingly modify synaptic tone onto BAT; gain or loss of action via this OX-controlled polysynaptic circuit can thus increase or diminish thermogenesis as required to match energy needs (Tupone et al., 2011). Loss of OX command neurons, however, decreases body temperature and stress-induced thermogenic response (Zhang et al., 2010; Sellayah et al., 2011). OX neurons also exert sympathetic control of muscle to couple energy levels with required physical response (Shiuchi et al., 2009).

## Neurotensin (Nts) neurons

### Discovery and characterization of Nts signaling

In 1973 Carraway and Leeman isolated a 13 amino acid peptide from bovine hypothalamus. Upon finding that intravenous Nts injection dilated blood vessels, lowered blood pressure and caused cyanosis in rats they coined this peptide Nts to reflect its pressor function (Carraway and Leeman, 1973). Most Nts (approximately 85%) is expressed within the intestine, but 10% of bodily Nts expression is within the brain. Nts is also found within the plasma (likely released from the intestine), but degrades rapidly so circulating Nts is unlikely to reach distant tissues, such as the brain, in an intact state (Lee et al., 1986). Nts is enriched in synaptosomes within the brain (Uhl and Snyder, 1977; Uhl et al., 1977) and is released after neuronal depolarization via a calcium dependent mechanism, signifying that Nts is a peptide neurotransmitter in the brain (Iversen et al., 1978). Nts in the brain is also rapidly degraded by membrane-bound angiotensin converting enzyme, proline endopeptidase and prohormone convertases-1 and -2, suggesting that it mediates short-acting signal transduction that is quickly inactivated (Checler et al., 1983; McDermott et al., 1986; Rovere et al., 1996a,b). Using *in situ* hybridization or radioimmunolabeling Nts was identified throughout the nervous system, including within the spinal cord, hindbrain (nucleus of solitary tract, LC, parabrachial nucleus), midbrain (periaqueductal gray, VTA, substantia nigra) limbic system (amygdala, hippocampus), forebrain (caudate putamen, NA), thalamus, and within the hypothalamus, particularly the preoptic area, PVN and the LHA (Kahn et al., 1982; Uhl, 1982; Roberts et al., 1984; Zahm, 1987; Allen and Cechetto, 1995). Immunohistochemical detection of Nts, however, requires treating rodents with colchicine, an anterograde transport inhibitor that leads to accumulation of proteins within the cell body, but which is inherently toxic and prevents study of these neurons in normal physiologic context (Kahn et al., 1982; Kalivas et al., 1982; Uhl, 1982; Bean et al., 1989). This technical limitation impeded study of Nts neurons, which is why there is comparatively less known about the Nts neurons in the LHA compared to MCH and OX neurons. It is important to note that Nts-expressing neurons are not restricted to the LHA. Yet, given the large population of LHA Nts neurons (Watts et al., 1999; Leinninger et al., 2011) and emerging reports of their specific roles in energy balance (Watts and Sanchez-Watts, 2007; Leinninger et al., 2011; Cui et al., 2012; Kempadoo et al., 2013; Opland et al., 2013), it is worth considering how Nts mediates behavior and body weight.

Nts binds to the G-protein coupled receptors, neurotensin receptor-1 (NtsR-1) and NtsR-2, (Mazella et al., 1985; Wang and Wu, 1996). A third receptor, neurotensin receptor-3 (NtsR-3, also called sortillin) is a single transmembrane receptor that binds Nts, but does not specifically transduce Nts signals (Mazella et al., 1998). NtsR-1 has high affinity for Nts, is predominantly expressed on neurons and is generally coupled to G_*q*_ proteins (Tanaka et al., 1990; Hermans et al., 1992). NtsR-2 exhibits low affinity Nts binding, is antagonized by the antihistamine levocabastine, may be expressed in neurons and astrocytes and can couple to G_*q*_ proteins. (Kitabgi et al., 1987; Mazella et al., 1996; Nouel et al., 1997, 1999; Yamauchi et al., 2007). There is also evidence that Nts binding to NtsR-2 is non-excitatory and can suppress NtsR-1 mediated activation, possibly via a G_*i/o*_-coupled mechanism (Yamada et al., 1998; Hwang et al., 2010). Nts binding assays in the rodent and human brain suggest that NtsRs are expressed within the cingulate cortex, midbrain (periaqueductal gray, DR, VTA, SN), subiculum and in the hindbrain (dorsal motor nucleus of the vagus, nucleus of the solitary tract, raphe pallidus, laterodorsal and pedunculopontine tegmental nuclei) (Kessler et al., 1987; Moyse et al., 1987; Najimi et al., 2014). NtsR-1 and NtsR-2 also bind xenin, a neuropeptide that is structurally similar to Nts but which is derived from a different gene product (Pozza et al., 1988; Feurle, 1998).

Nts specifically promotes activation of NtsR-expressing neurons in the prefrontal cortex, VLPAG, substantia nigra and in VTA DA neurons (Behbehani et al., 1987; Audinat et al., 1989; Seutin et al., 1989). In general, Nts action via NtsRs induces a non-selective cation current to promote neuronal depolarization (Lu et al., 2009). Additionally, on DA neurons Nts opposes the inhibitory effects of D2R signaling, thus Nts acts via dual mechanisms to promote the activation of DA neurons (Shi and Bunney, 1991; Farkas et al., 1997; Werkman et al., 2000; Legault et al., 2002). Indeed, within the VTA Nts-containing axon terminals are primarily apposed with DA neurons (Woulfe and Beaudet, 1992) and Nts acts via the VTA to promote DA release into the NA and modify reward behavior (Blaha et al., 1990; Singh et al., 1997). Both NtsR-1 and NtsR-2 are implicated in regulating DA neurons (Szigethy and Beaudet, 1989; Nalivaiko et al., 1998; Sotty et al., 1998), but treatment with NtsR-1-sepecific antagonists blocks Nts-mediated DA release from midbrain neurons (Gully et al., 1993; Brouard et al., 1994). Collectively these data suggest that NtsR-1 is the predominant receptor isoform in neurotensin-mediated regulation of VTA DA neurons.

The development of mice that express cre recombinase in Nts neurons (*Nts^Cre^* mice) permitted the facile identification of Nts neurons throughout the brain, including a large population of Nts neurons within the LHA (Leinninger et al., 2011). These *Nts^Cre^* mice, when bred onto a cre-mediated green fluorescent reporter line, identify LHA Nts neurons that are distinct from adjacent neurons expressing MCH or OX, similar to previous reports that identified Nts, OX and MCH via *in situ* hybridization or colchicine-mediated immunostaining (Watts and Sanchez-Watts, 2007; Leinninger et al., 2011; Laque et al., 2013). LHA Nts neurons, however, are not a homogenous population; there are subpopulations of Nts-containing neurons within the LHA with distinct molecular signatures, though these have yet to be fully characterized. Some LHA Nts neurons co-express the long form of the LepRb and are activated by leptin (Leinninger et al., 2011) and some of these neurons also co-express the inhibitory neuropeptide galanin and/or melanocortin-4 receptor (Cui et al., 2012; Laque et al., 2013). Other subpopulations of Nts neurons contain CRH (Watts and Sanchez-Watts, 2007) or MCHR-1 (Chee et al., 2013). Further, some LHA Nts neurons co-express the classical neurotransmitters GABA or glutamate (Leinninger et al., 2011; Kempadoo et al., 2013). As a whole, LHA Nts neurons project densely within the LHA to OX neurons and also to the VTA, via which they likely regulate DA neurons. Indeed, activation of LHA Nts neurons potentiates the activation of VTA DA neurons via an NtsR1-dependent mechanism and mice self-stimulate LHA Nts neurons, presumably because it is rewarding (Kempadoo et al., 2013). LHA Nts neurons also regulate the activity of OX neurons via mechanisms that remain to be determined (Leinninger et al., 2011; Furutani et al., 2013; Goforth et al., 2014). Thus, LHA Nts neurons exert control of OX neurons and VTA DA neurons that could (as established above) modulate feeding, drinking and locomotor activity. While the precise roles of LHA Nts neurons in these physiologic behaviors have yet to be fully elucidated, there is a large literature to suggest that central Nts can indeed modify behaviors relevant to energy balance, as reviewed below.

### Nts and feeding

Central Nts modestly decreases food intake in satiated and food-deprived rodents, in part via actions in the substantia nigra and VTA (Stanley et al., 1983; Hawkins, 1986a,b; Vaughn et al., 1990; Boules et al., 2000). Administration of Nts into the LHA or ventral striatum, however, does not alter feeding, suggesting either a lack of NtsRs in these regions or that they regulate other aspects of behavior (Hawkins, 1986b; Hawkins et al., 1986). Loss of Nts expression might therefore be expected to promote feeding and exacerbate weight gain. Indeed hyperphagic, obese rodents have reduced Nts expression in the brain, including within the hypothalamus, that may contribute to the disease state (Sheppard et al., 1985; Beck et al., 1989, 1990, 1992; Williams et al., 1991; Wilding et al., 1993).

Nts neurons are regulated by the anorectic hormone leptin, suggesting coordinated roles of Nts and leptin to modify feeding and body weight. Chronic leptin treatment decreases food intake and body weight as expected, and also decreases *Nts* expression within the LHA (Sahu, 1998; Richy et al., 2000). By contrast, acute leptin treatment of hypothalamic-derived cell lines increases *Nts* expression (Cui et al., 2006). NtsR-1 is the essential receptor isoform for Nts-mediated suppression of feeding (Remaury et al., 2002; Feifel et al., 2010). Nts potentiates leptin-mediated inhibition of feeding via NtsR-1 (Beck et al., 1998; Sahu et al., 2001) but mice deficient in NtsR-1 have an impaired anorectic response to leptin, confirming that leptin and Nts/NtsR-1 synergistically modify feeding (Kim et al., 2008). Intriguingly, the leptin/NtsR-1 system may have more impact in regulating non-homeostatic feeding: while mice lacking NtsR1 exhibit normal chow intake, they over consume palatable, high-fat diet or a sucrose solution that promotes obesity (Opland et al., 2013). The LHA is the site of leptin and Nts synergy: approximately 30% of Nts neurons co-express LepRb, the co-expressing neurons are exclusively found within the LHA, and represent the only Nts neurons in the brain that are directly activated by leptin. Deletion of LepRb specifically from LHA Nts-LepRb neurons promotes mild hyperphagia and obesity in mice (Leinninger et al., 2011). Furthermore, intact NtsR-1 expression is required for LHA Nts-LepRb neurons to restrain feeding, indicating the functional integration of leptin and Nts/NtsR-1 action (Opland et al., 2013). In this regard, stimulating NtsR-1 neurons (similar to leptin-mediated Nts release from LHA Nts-LepRb neurons) may be useful to suppress feeding and body weight. Indeed, brain permeable NtsR-1-specific agonists decrease feeding and body weight in normal mice, as well as in leptin-deficient obese mice, suggesting that Nts action via NtsR-1 may be useful in treating obesity (Feifel et al., 2010).

LHA Nts neurons, including Nts-LepRb neurons, likely exert some regulation of feeding via their projections to the VTA (Leinninger et al., 2011; Opland et al., 2013). Nts activates VTA neurons, promotes reinforcement (Glimcher et al., 1984) and rats will self administer Nts as if it is rewarding (Glimcher et al., 1987). Similarly, activation of LHA Nts neurons promotes reward responding (Kempadoo et al., 2013) though it is unclear how this modifies feeding. Give that Nts-mediated anorexia is enhanced by co-administration with DA agonists (Hawkins et al., 1986), activation of LHA Nts neurons may stimulate VTA DA neurons to suppress feeding. LHA Nts neurons may also project to the parabrachial nucleus (Moga et al., 1990), and it is possible that Nts contributes to anorectic drive via this brain region (Carter et al., 2013). Additionally, some LHA Nts neurons co-express MC4R and LepRb (but not OX or MCH) and may be regulated via melanocortins to modulate feeding (Cui et al., 2012).

### Nts and drinking

Water deprivation or osmotic stimulation specifically increases *Nts* expression in the LHA (Watts, 1992; Watts et al., 1999). Sensing of water deficit occurs, at least in part, via the subpopulation of LHA Nts neurons that co-express CRH (Watts et al., 1995). There are a modest number of CRH-containing neurons in the LHA of rats, most of which co-express Nts, but overall these CRH-Nts neurons represent a minority of the total LHA Nts neurons (Watts and Sanchez-Watts, 2007). Interestingly, water deprivation likely increases the activity of LHA Nts neurons while OX neuronal activity is only increased after drinking (Watts and Sanchez-Watts, 2007). It is thus tempting to speculate that Nts neurons signal the degree of “thirst” while activation of OX neurons drives water intake, if water is available. This could explain why rodents with *ad libitum* water will drink after central Nts treatment [here the Nts-signaled “thirst” can be instantly resolved by drinking] (Stanley et al., 1983; Quirk et al., 1988; Baker et al., 1989; Sandoval and Kulkosky, 1992) or after direct activation of OX neurons that promotes intake behavior (Inutsuka et al., 2014). Moreover, Nts may promote water sensing via stimulating stretch-activated cation channels that mediate osmoreception and sodium detection, thereby potentiating hypertonic stimulation of cells with these channels (Chakfe and Bourque, 2000). Nts may also have a general role in inhibiting intake of “rewarding” liquids such as ethanol (Lee et al., 2010, 2011), sucrose (Opland et al., 2013) or thirst-induced water intake, which may be itself be pleasurable after dehydration.

### Nts and energy expenditure

#### Nts and physical activity

Nts exerts brain site-specific control of locomotor activity. Nts in the NA (which is not directly regulated by LHA Nts neurons) suppresses locomotor activity (Ervin et al., 1981; Kalivas et al., 1982; Nemeroff et al., 1982; Jolicoeur et al., 1985; Skoog et al., 1986). By contrast, Nts treatment in the VTA promotes locomotor activity along with DA output into NA and olfactory tubercle (Kalivas et al., 1981, 1983, 1985a; Kalivas and Duffy, 1990). Given that LHA Nts neurons project to the VTA, Nts released from these neurons may similarly promote DA-mediated physical activity (Leinninger et al., 2011; Kempadoo et al., 2013; Opland et al., 2013). NtsR-1 is an essential mediator of Nts-mediated locomotor activity in the VTA (Steinberg et al., 1994). Chronic Nts administration into the VTA causes long-lasting sensitization and progressively increased locomotor activity even after treatment is suspended, suggesting that sustained, endogenous release of Nts remodels VTA circuits to modulate locomotor output (Kalivas and Taylor, 1985; Elliott and Nemeroff, 1986). Since LHA Nts neurons project to and can activate NtsR1-expressing DA neurons (Leinninger et al., 2011; Opland et al., 2013), they may promote locomotor activity via NtsR1-dependent actions in the VTA. Indeed, silencing the 30% of LHA Nts neurons that co-express LepRb, and presumably blunting Nts release to the VTA, reduces locomotor activity and disrupts DA signaling (Leinninger et al., 2011). Functionally, Nts action in the VTA may also exert antidepressant effects, as it increases forced swim efforts even at sub-threshold doses that do not promote general locomotor increase (Cervo et al., 1992). Stress increases Nts in the VTA, perhaps to potentiate adaptive locomotor behaviors needed for survival (Deutch et al., 1987; Wachi et al., 1987). Central or systemic Nts, however, diminishes locomotor effects, suggesting that Nts actions in the NA outweigh those via the VTA (Kalivas et al., 1983; Elliott et al., 1986; Vadnie et al., 2014). The cellular localization of NtsRs likely accounts for the differential control of locomotor activity via NA and VTA neurons. VTA DA neurons express NtsRs at the dendrites and soma, so Nts action via stimulatory NtsRs promotes activation of DA neurons and release of DA into the NA that induces locomotor activity. By contrast, Nts injected directly into NA acts via NtsRs on the dendrites and soma of GABAergic spiny neurons (Herve et al., 1986). Increasing Nts action via the NA may be useful to suppress the excessive locomotor effects in schizophrenia, similar to the effects of antipsychotics (Binder et al., 2001). LHA Nts neurons, however, do not project into the ventral or dorsal striatum, and thus likely promote locomotor activity via projections to, and regulation of VTA DA neurons (Leinninger et al., 2011; Opland et al., 2013).

LHA Nts neurons additionally project within the LHA and modulate OX neurons (Leinninger et al., 2011; Goforth et al., 2014), which also regulate ambulatory activity. Some Nts neurons in the LHA are activated by inflammatory signals, but adjacent OX neurons are inhibited in these conditions, suggesting differential control of these neuronal populations (Grossberg et al., 2011). LHA Nts neurons project onto and inhibit OX neurons (Goforth et al., 2014), thus inflammation or illness-mediated activation of Nts neurons can presumably suppress the activity of OX neurons and decrease locomotor activity during these states (Grossberg et al., 2011; Leinninger et al., 2011). Similarly, loss of action via LHA Nts-LepRb neurons decreases locomotor activity and energy expenditure in mice that promotes obesity (Leinninger et al., 2011), and some portion of these effects are likely mediated via regulation of OX neurons (Leinninger et al., 2011; Opland et al., 2013).

#### Nts and arousal/sleep

To date there are no direct reports concerning the role of LHA Nts neurons in arousal. Burdakov described a large population of non-MCH, non-OX GABAergic neurons in the LHA that are spontaneously active during waking and sleeping periods, and it is possible that these are Nts neurons (Karnani et al., 2013). There were four subtypes of these uncharacterized GABAergic neurons, each of which exhibited distinct electrophysiogic properties (firing rate/response). Similarly, LHA Nts neurons are genetically heterogeneous (e.g., vary in expression of LepRb, galanin, melanocortin-4 receptor, CRH, as discussed above), and many of them are GABAergic (Leinninger et al., 2009), so it is possible that these electrophysiologically distinct populations are in fact subpopulations of LHA Nts neurons (Hassani et al., 2010; Sapin et al., 2010; Karnani et al., 2013). Central administration of Nts promotes alertness and prolongs latency to sleep stages, suggesting that LHA Nts neurons could play a role in sustained arousal (Castel et al., 1989).

#### Nts and sympathetic control

Central Nts increases analgesia (Clineschmidt et al., 1979; Osbahr et al., 1981), possibly via LHA Nts neurons that project to and activate neurons in the VLPAG (Shipley et al., 1987). Inhibitors of Nts degradation also enhance Nts-mediated analgesia, (Vincent et al., 1997) and both NtsR-1 and NtsR-2 contribute to Nts-mediated analgesia (Dubuc et al., 1999; Pettibone et al., 2002; Remaury et al., 2002). Site-specific Nts agonists may have potential use as analgesics, and would lack the addictive properties of opioid-based drugs that are currently used for pain treatment (Kleczkowska and Lipkowski, 2013). Nts also lowers body temperature (Bissette et al., 1976, 1982; Nemeroff et al., 1977; Martin et al., 1980). Many non-OX LHA neurons project to the raphe pallidus and are poised to regulate somatic and sympathetic systems (e.g., BAT); these could be Nts neurons (Tupone et al., 2011). Nts fibers have also been detected in the solitary nucleus and caudal medulla, and are accordingly poised to regulate cardiac and vagal preganglionic motor neurons (Behbehani and Pert, 1984; Higgins et al., 1984; Griffiths et al., 1986; Behbehani et al., 1987, 1988; Fang et al., 1987; Kawai et al., 1988). Nts may also act via the VTA to promote hypothermia (Kalivas et al., 1985b; Popp et al., 2007). Nts–meditated analgesia and suppression of body temperature may support physiologic alertness and contribute to the anxiolytic effects of Nts (Hou et al., 2011). LHA Nts neurons are specifically responsive to stress (Seta et al., 2001) so it is possible that Nts released to the VLPAG, caudal medulla, and VTA may collectively promote analgesia, autonomic outflow and locomotor activity to support survival, such as fight or flight behaviors, but this has yet remains to be experimentally verified.

## Summary and implications: the LHA is “tuned” to coordinate energy cues and behavior

The LHA has now long-been regarded as an essential brain region for promoting feeding, drinking and energy expenditure behaviors that inherently modify energy balance and weight. The LHA, however, appears to coordinate a diverse array of peripheral cues that range in nature from hormones, nutrient levels, osmolality, light and sound as well as neurotransmitter inputs from other brain centers. From an anatomical perspective, the large LHA contains multiple populations of neurons that project to virtually every region of the brain. Thus, the LHA seemingly manages the daunting task of receiving and interpreting cues from the body, and then matching appropriate behaviors and physiologic regulation to each cue via a broad network of neuronal regulation. The discovery of neuropeptide-specific populations within the LHA has catapulted the field’s understanding of how the LHA can manage such a formidable task: via specialized, cue-sensitive neurons that mediate actions via distinct neuronal projections. While the characterizations of MCH, OX and Nts neurons have begun to illuminate specialized functions of each population, these studies are only the tip of the iceberg, so to speak. As we drill down to understanding the precise molecular signature of OX, MCH and Nts neurons we are likely to identify even further specialized subpopulations of these neurons. It is tempting to speculate that, perhaps, specified subpopulations mediate the response to particular physiologic cues and match behavior to restore energy balance. Such parceling would enable targeting of therapies to either amplify or inhibit feeding, drinking, physical activity, antinociception and arousal, and accordingly ameliorate a number of health conditions. In reality, it is unlikely that the neuronal signatures will be easily identifiable or so conveniently specialized, as neuronal systems tend to overlap in function and projections. Indeed, the fact that MCH, OX and Nts neurons are neuropeptide-distinct but project to and regulate similar neuronal targets and sites within the brains speaks to this fact. Even so, MCH, OX and Nts neurons do contribute differential physiologic regulation. MCH neurons largely regulate physical activity and energy expenditure but do not contribute as abundantly to regulation of arousal. OX neurons are the predominant LHA neuronal mediators of arousal, and must integrally regulate feeding, activity and sympathetic outflow to support alertness. Inherently, both MCH and OX neurons may increase feeding to support energy-consuming physical activity and alertness. In contrast, the few studies to date suggest that LHA Nts neurons are required to restrain feeding and promote physical activity. Thus overall, there are likely to be distinct physiologic contributions of these neuropeptide-specific populations that permit the LHA’s impressive regulation of motivation, energy balance and survival. Just as the discovery of neuronal heterogeneity in the LHA prompted the idea of functional divergence, utilization of reagents to selectively identify and modulate MCH, OX and Nts neurons will reveal their selective circuitry and functional contributions to energy balance.

## Conflict of interest statement

The authors declare that the research was conducted in the absence of any commercial or financial relationships that could be construed as a potential conflict of interest.

## References

[B1] Abizaid A. Liu Z. W. Andrews Z. B. Shanabrough M. Borok E. Elsworth J. D.. (2006). Ghrelin modulates the activity and synaptic input organization of midbrain dopamine neurons while promoting appetite. J. Clin. Invest. 116, 3229–3239. 10.1172/jci29867 17060947 PMC1618869

[B2] Acuna-Goycolea C. van den Pol A. (2004). Glucagon-like peptide 1 excites hypocretin/orexin neurons by direct and indirect mechanisms: implications for viscera-mediated arousal. J. Neurosci. 24, 8141–8152. 10.1523/jneurosci.1607-04.2004 15371515 PMC6729787

[B3] Adamantidis A. R. Zhang F. Aravanis A. M. Deisseroth K. De Lecea L. (2007). Neural substrates of awakening probed with optogenetic control of hypocretin neurons. Nature 450, 420–424. 10.1038/nature06310 17943086 PMC6744371

[B4] Adler E. S. Hollis J. H. Clarke I. J. Grattan D. R. Oldfield B. J. (2012). Neurochemical characterization and sexual dimorphism of projections from the brain to abdominal and subcutaneous white adipose tissue in the rat. J. Neurosci. 32, 15913–15921. 10.1523/jneurosci.2591-12.2012 23136429 PMC6621617

[B5] Ahnaou A. Drinkenburg W. H. Bouwknecht J. A. Alcazar J. Steckler T. Dautzenberg F. M. (2008). Blocking melanin-concentrating hormone MCH1 receptor affects rat sleep-wake architecture. Eur. J. Pharmacol. 579, 177–188. 10.1016/j.ejphar.2007.10.017 18062961

[B6] Akiyama M. Yuasa T. Hayasaka N. Horikawa K. Sakurai T. Shibata S. (2004). Reduced food anticipatory activity in genetically orexin (hypocretin) neuron-ablated mice. Eur J. Neurosci. 20, 3054–3062. 10.1111/j.1460-9568.2004.03749.x 15579160

[B7] Alam M. N. Kumar S. Bashir T. Suntsova N. Methippara M. M. Szymusiak R.. (2005). GABA-mediated control of hypocretin- but not melanin-concentrating hormone-immunoreactive neurones during sleep in rats. J. Physiol. 563, 569–582. 10.1113/jphysiol.2004.076927 15613374 PMC1665577

[B8] Allen G. V. Cechetto D. F. (1995). Neurotensin in the lateral hypothalamic area: origin and function. Neuroscience 69, 533–544. 10.1016/0306-4522(95)00261-g 8552247

[B9] An S. Cutler G. Zhao J. J. Huang S. G. Tian H. Li W.. (2001). Identification and characterization of a melanin-concentrating hormone receptor. Proc. Natl. Acad. Sci. U S A 98, 7576–7581. 10.1074/jbc.m102601200 11416225 PMC34710

[B10] Anaclet C. Parmentier R. Ouk K. Guidon G. Buda C. Sastre J. P.. (2009). Orexin/hypocretin and histamine: distinct roles in the control of wakefulness demonstrated using knock-out mouse models. J. Neurosci. 29, 14423–14438. 10.1523/jneurosci.2604-09.2009 19923277 PMC2802289

[B11] Anand B. K. (1980). This week’s citation classic: Anand BK and Brobeck JR. Hypothalamic control of food intake in rats and cats. Yale J. Biol. Med. 24, 123–140. 14901884 PMC2599116

[B12] Anand B. K. Brobeck J. R. (1951a). Hypothalamic control of food intake in rats and cats. Yale J. Biol. Med. 24, 123–140. 14901884 PMC2599116

[B13] Anand B. K. Brobeck J. R. (1951b). Localization of a “feeding center” in the hypothalamus of the rat. Proc. Soc. Exp. Biol. Med. 77, 323–324. 10.3181/00379727-77-18766 14854036

[B14] Anderson R. I. Becker H. C. Adams B. L. Jesudason C. D. Rorick-Kehn L. M. (2014). Orexin-1 and orexin-2 receptor antagonists reduce ethanol self-administration in high-drinking rodent models. Front. Neurosci. 8:33. 10.3389/fnins.2014.00033 24616657 PMC3933945

[B15] Appelbaum L. Wang G. Yokogawa T. Skariah G. M. Smith S. J. Mourrain P.. (2010). Circadian and homeostatic regulation of structural synaptic plasticity in hypocretin neurons. Neuron 68, 87–98. 10.1016/j.neuron.2010.09.006 20920793 PMC2969179

[B16] Audinat E. Hermel J. M. Crépel F. (1989). Neurotensin-induced excitation of neurons of the rat’s frontal cortex studied intracellularly in vitro. Exp. Brain Res. 78, 358–368. 10.1007/bf00228907 2599044

[B17] Aziz A. Fronczek R. Maat-Schieman M. Unmehopa U. Roelandse F. Overeem S.. (2008). Hypocretin and melanin-concentrating hormone in patients with Huntington disease. Brain Pathol. 18, 474–483. 10.1111/j.1750-3639.2008.00135.x 18498421 PMC8095609

[B18] Bailey A. P. Parker A. G. Colautti L. A. Hart L. M. Liu P. Hetrick S. E. (2014). Mapping the evidence for the prevention and treatment of eating disorders in young people. J. Eat. Disord. 2:5. 10.1186/2050-2974-2-5 24999427 PMC4081733

[B19] Baird J. P. Gray N. E. Fischer S. G. (2006a). Effects of neuropeptide Y on feeding microstructure: dissociation of appetitive and consummatory actions. Behav. Neurosci. 120, 937–951. 10.1037/0735-7044.120.4.937 16893299

[B20] Baird J. P. Rios C. Gray N. E. Walsh C. E. Fischer S. G. Pecora A. L. (2006b). Effects of melanin-concentrating hormone on licking microstructure and brief-access taste responses. Am. J. Physiol. Regul. Integr. Comp. Physiol. 291, R1265–R1274. 10.1152/ajpregu.00143.2006 16763081

[B21] Baird J. P. Rios C. Loveland J. L. Beck J. Tran A. Mahoney C. E. (2008). Effects of hindbrain melanin-concentrating hormone and neuropeptide Y administration on licking for water, saccharin and sucrose solutions. Am. J. Physiol. Regul. Integr. Comp. Physiol. 294, R329–R343. 10.1152/ajpregu.00611.2006 17989139 PMC3522464

[B22] Baker J. D. Hawkins M. F. Baumeister A. A. Nagy M. (1989). Microinjection of neurotensin into the CNS induces hyperdipsia in the rat. Pharmacol. Biochem. Behav. 33, 7–10. 10.1016/0091-3057(89)90420-6 2780791

[B23] Basso A. M. Bratcher N. A. Gallagher K. B. Cowart M. D. Zhao C. Sun M.. (2006). Lack of efficacy of melanin-concentrating hormone-1 receptor antagonists in models of depression and anxiety. Eur. J. Pharmacol. 540, 115–120. 10.1016/j.ejphar.2006.04.043 16765941

[B24] Baumann C. R. Bassetti C. L. Valko P. O. Haybaeck J. Keller M. Clark E.. (2009). Loss of hypocretin (orexin) neurons with traumatic brain injury. Ann. Neurol. 66, 555–559. 10.1002/ana.21836 19847903 PMC2770195

[B25] Baumann C. R. Stocker R. Imhof H. G. Trentz O. Hersberger M. Mignot E.. (2005). Hypocretin-1 (orexin A) deficiency in acute traumatic brain injury. Neurology 65, 147–149. 10.1212/01.wnl.0000167605.02541.f2 16009905

[B26] Bayer L. Eggermann E. Serafin M. Saint-Mleux B. Machard D. Jones B.. (2001). Orexins (hypocretins) directly excite tuberomammillary neurons. Eur J. Neurosci. 14, 1571–1575. 10.1046/j.0953-816x.2001.01777.x 11722619

[B27] Bean A. J. During M. J. Deutch A. Y. Roth R. H. (1989). Effects of dopamine depletion on striatal neurotensin: biochemical and immunohistochemical studies. J. Neurosci. 9, 4430–4438. 2593006 10.1523/JNEUROSCI.09-12-04430.1989 PMC6569647

[B28] Beck B. Burlet A. Nicolas J. P. Burlet C. (1989). Neurotensin in microdissected brain nuclei and in the pituitary of the lean and obese Zucker rats. Neuropeptides 13, 1–7. 10.1016/0143-4179(89)90014-0 2922104

[B29] Beck B. Burlet A. Nicolas J. P. Burlet C. (1990). Hyperphagia in obesity is associated with a central peptidergic dysregulation in rats. J. Nutr. 120, 806–811. 2366113 10.1093/jn/120.7.806

[B30] Beck B. Stricker-Krongrad A. Burlet A. Nicolas J. P. Burlet C. (1992). Changes in hypothalamic neurotensin concentrations and food intake in rats fed a high fat diet. Int. J. Obes. Relat. Metab. Disord. 16, 361–366. 1319971

[B31] Beck B. Stricker-Krongrad A. Richy S. Burlet C. (1998). Evidence that hypothalamic neurotensin signals leptin effects on feeding behavior in normal and fat-preferring rats. Biochem. Biophys. Res. Commun. 252, 634–638. 10.1006/bbrc.1998.9712 9837758

[B32] Becskei C. Riediger T. Hernádfalvy N. Arsenijevic D. Lutz T. A. Langhans W. (2008). Inhibitory effects of lipopolysaccharide on hypothalamic nuclei implicated in the control of food intake. Brain Behav. Immun. 22, 56–64. 10.1016/j.bbi.2007.06.002 17624718

[B34] Behbehani M. M. Park M. R. Clement M. E. (1988). Interactions between the lateral hypothalamus and the periaqueductal gray. J. Neurosci. 8, 2780–2787. 2900881 10.1523/JNEUROSCI.08-08-02780.1988 PMC6569401

[B33] Behbehani M. M. Pert A. (1984). A mechanism for the analgesic effect of neurotensin as revealed by behavioral and electrophysiological techniques. Brain Res. 324, 35–42. 10.1016/0006-8993(84)90619-x 6518391

[B35] Behbehani M. M. Shipley M. T. Mclean J. H. (1987). Effect of neurotensin on neurons in the periaqueductal gray: an in vitro study. J. Neurosci. 7, 2035–2040. 3612228 10.1523/JNEUROSCI.07-07-02035.1987 PMC6568933

[B36] Berridge K. C. Ho C. Y. Richard J. M. Difeliceantonio A. G. (2010). The tempted brain eats: pleasure and desire circuits in obesity and eating disorders. Brain Res. 1350, 43–64. 10.1016/j.brainres.2010.04.003 20388498 PMC2913163

[B37] Berthoud H. R. (2012). The neurobiology of food intake in an obesogenic environment. Proc. Nutr. Soc. 71, 478–487. 10.1017/s0029665112000602 22800810 PMC3617987

[B38] Beuckmann C. T. Sinton C. M. Williams S. C. Richardson J. A. Hammer R. E. Sakurai T.. (2004). Expression of a poly-glutamine-ataxin-3 transgene in orexin neurons induces narcolepsy-cataplexy in the rat. J. Neurosci. 24, 4469–4477. 10.1523/jneurosci.5560-03.2004 15128861 PMC6729432

[B39] Binder E. B. Kinkead B. Owens M. J. Kilts C. D. Nemeroff C. B. (2001). Enhanced neurotensin neurotransmission is involved in the clinically relevant behavioral effects of antipsychotic drugs: evidence from animal models of sensorimotor gating. J. Neurosci. 21, 601–608. 11160439 10.1523/JNEUROSCI.21-02-00601.2001 PMC6763810

[B40] Bissette G. Luttinger D. Mason G. A. Hernandez D. E. Loosen P. T. (1982). Neurotensin and thermoregulation. Ann. N Y Acad. Sci. 400, 268–282. 10.1111/j.1749-6632.1982.tb31575.x 6820243

[B41] Bissette G. Nemeroff C. B. Loosen P. T. Prange A. J. Jr. Lipton M. A. (1976). Hypothermia and intolerance to cold induced by intracisternal administration of the hypothalamic peptide neurotensin. Nature 262, 607–609. 10.1038/262607a0 8728

[B42] Bittencourt J. C. Elias C. F. (1998). Melanin-concentrating hormone and neuropeptide EI projections from the lateral hypothalamic area and zona incerta to the medial septal nucleus and spinal cord: a study using multiple neuronal tracers. Brain Res. 805, 1–19. 10.1016/s0006-8993(98)00598-8 9733903

[B43] Bittencourt J. C. Presse F. Arias C. Peto C. Vaughan J. Nahon J. L.. (1992). The melanin-concentrating hormone system of the rat brain: an immuno- and hybridization histochemical characterization. J. Comp. Neurol. 319, 218–245. 10.1002/cne.903190204 1522246

[B44] Blaha C. D. Coury A. Fibiger H. C. Phillips A. G. (1990). Effects of neurotensin on dopamine release and metabolism in the rat striatum and nucleus accumbens: cross-validation using in vivo voltammetry and microdialysis. Neuroscience 34, 699–705. 10.1016/0306-4522(90)90176-5 2352647

[B45] Blouin A. M. Fried I. Wilson C. L. Staba R. J. Behnke E. J. Lam H. A.. (2013). Human hypocretin and melanin-concentrating hormone levels are linked to emotion and social interaction. Nat. Commun. 4:1547. 10.1038/ncomms2461 23462990 PMC3595130

[B46] Borgland S. L. Chang S. J. Bowers M. S. Thompson J. L. Vittoz N. Floresco S. B.. (2009). Orexin A/hypocretin-1 selectively promotes motivation for positive reinforcers. J. Neurosci. 29, 11215–11225. 10.1523/jneurosci.6096-08.2009 19741128 PMC2771749

[B47] Borgland S. L. Taha S. A. Sarti F. Fields H. L. Bonci A. (2006). Orexin A in the VTA is critical for the induction of synaptic plasticity and behavioral sensitization to cocaine. Neuron 49, 589–601. 10.1016/j.neuron.2006.01.016 16476667

[B48] Borowsky B. Durkin M. M. Ogozalek K. Marzabadi M. R. Deleon J. Lagu B.. (2002). Antidepressant, anxiolytic and anorectic effects of a melanin-concentrating hormone-1 receptor antagonist. Nat. Med. 8, 825–830. 10.1038/nm0902-1039b 12118247

[B49] Boules M. Cusack B. Zhao L. Fauq A. Mccormick D. J. Richelson E. (2000). A novel neurotensin peptide analog given extracranially decreases food intake and weight in rodents. Brain Res. 865, 35–44. 10.1016/s0006-8993(00)02187-9 10814731

[B50] Bourgin P. Huitrón-Résendiz S. Spier A. D. Fabre V. Morte B. Criado J. R.. (2000). Hypocretin-1 modulates rapid eye movement sleep through activation of locus coeruleus neurons. J. Neurosci. 20, 7760–7765. 11027239 10.1523/JNEUROSCI.20-20-07760.2000 PMC6772862

[B51] Bray G. A. Ryan D. H. (2014). Update on obesity pharmacotherapy. Ann. N Y Acad. Sci. 1311, 1–13. 10.1111/nyas.12328 24641701

[B52] Brisbare-Roch C. Dingemanse J. Koberstein R. Hoever P. Aissaoui H. Flores S.. (2007). Promotion of sleep by targeting the orexin system in rats, dogs and humans. Nat. Med. 13, 150–155. 10.1038/nm0507-525 17259994

[B53] Brischoux F. Cvetkovic V. Griffond B. Fellmann D. Risold P. Y. (2002). Time of genesis determines projection and neurokinin-3 expression patterns of diencephalic neurons containing melanin-concentrating hormone. Eur J. Neurosci. 16, 1672–1680. 10.1046/j.1460-9568.2002.02229.x 12431219

[B54] Brouard A. Heaulme M. Leyris R. Pelaprat D. Gully D. Kitabgi P.. (1994). SR 48692 inhibits neurotensin-induced [3H]dopamine release in rat striatal slices and mesencephalic cultures. Eur. J. Pharmacol. 253, 289–291. 10.1016/0014-2999(94)90204-6 8200423

[B55] Bruning J. C. Gautam D. Burks D. J. Gillette J. Schubert M. Orban P. C.. (2000). Role of brain insulin receptor in control of body weight and reproduction. Science 289, 2122–2125. 10.1126/science.289.5487.2122 11000114

[B56] Burdakov D. Gerasimenko O. Verkhratsky A. (2005). Physiological changes in glucose differentially modulate the excitability of hypothalamic melanin-concentrating hormone and orexin neurons in situ. J. Neurosci. 25, 2429–2433. 10.1523/jneurosci.4925-04.2005 15745970 PMC6726089

[B57] Cai X. J. Lister C. A. Buckingham R. E. Pickavance L. Wilding J. Arch J. R.. (2000). Down-regulation of orexin gene expression by severe obesity in the rats: studies in Zucker fatty and zucker diabetic fatty rats and effects of rosiglitazone. Brain Res. Mol. Brain Res. 77, 131–137. 10.1016/s0169-328x(00)00041-3 10814839

[B58] Cai X. J. Widdowson P. S. Harrold J. Wilson S. Buckingham R. E. Arch J. R.. (1999). Hypothalamic orexin expression: modulation by blood glucose and feeding. Diabetes 48, 2132–2137. 10.2337/diabetes.48.11.2132 10535445

[B59] Carraway R. Leeman S. E. (1973). The isolation of a new hypotensive peptide, neurotensin, from bovine hypothalami. J. Biol. Chem. 248, 6854–6861. 4745447

[B60] Carter M. E. Adamantidis A. Ohtsu H. Deisseroth K. De Lecea L. (2009). Sleep homeostasis modulates hypocretin-mediated sleep-to-wake transitions. J. Neurosci. 29, 10939–10949. 10.1523/JNEUROSCI.1205-09.2009 19726652 PMC3849591

[B61] Carter M. E. Brill J. Bonnavion P. Huguenard J. R. Huerta R. De Lecea L. (2012). Mechanism for Hypocretin-mediated sleep-to-wake transitions. Proc. Natl. Acad. Sci. U S A 109, E2635–E2644. 10.1073/pnas.1202526109 22955882 PMC3465396

[B62] Carter M. E. Soden M. E. Zweifel L. S. Palmiter R. D. (2013). Genetic identification of a neural circuit that suppresses appetite. Nature 503, 111–114. 10.1038/nature12596 24121436 PMC3878302

[B63] Cason A. M. Aston-Jones G. (2013a). Attenuation of saccharin-seeking in rats by orexin/hypocretin receptor 1 antagonist. Psychopharmacology (Berl) 228, 499–507. 10.1007/s00213-013-3051-7 23494235 PMC3707982

[B64] Cason A. M. Aston-Jones G. (2013b). Role of orexin/hypocretin in conditioned sucrose-seeking in rats. Psychopharmacology (Berl) 226, 155–165. 10.1007/s00213-012-2902-y 23096770 PMC3572270

[B65] Castel M. N. Stutzmann J. M. Lucas M. Lafforgue J. Blanchard J. C. (1989). Effects of ICV administration of neurotensin and analogs on EEG in rats. Peptides 10, 95–101. 10.1016/0196-9781(89)90083-1 2748429

[B66] Cervo L. Rossi C. Tatarczynska E. Samanin R. (1992). Antidepressant-like effect of neurotensin administered in the ventral tegmental area in the forced swimming test. Psychopharmacology (Berl) 109, 369–372. 10.1007/bf02245885 1365637

[B67] Chakfe Y. Bourque C. W. (2000). Excitatory peptides and osmotic pressure modulate mechanosensitive cation channels in concert. Nat. Neurosci. 3, 572–579. 10.1038/78732 10816313

[B68] Chambers J. Ames R. S. Bergsma D. Muir A. Fitzgerald L. R. Hervieu G.. (1999). Melanin-concentrating hormone is the cognate ligand for the orphan G-protein-coupled receptor SLC-1. Nature 400, 261–265. 10.1016/s0014-5793(99)01092-3 10421367

[B69] Checler F. Vincent J. P. Kitabgi P. (1983). Degradation of neurotensin by rat brain synaptic membranes: involvement of a thermolysin-like metalloendopeptidase (enkephalinase), angiotensin-converting enzyme and other unidentified peptidases. J. Neurochem. 41, 375–384. 10.1111/j.1471-4159.1983.tb04753.x 6308159

[B70] Chee M. J. Pissios P. Maratos-Flier E. (2013). Neurochemical characterization of neurons expressing melanin-concentrating hormone receptor 1 in the mouse hypothalamus. J. Comp. Neurol. 521, 2208–2234. 10.1002/cne.23273 23605441 PMC3633152

[B71] Chee M. J. Pissios P. Prasad D. Maratos-Flier E. (2014). Expression of melanin-concentrating hormone receptor 2 protects against diet-induced obesity in male mice. Endocrinology 155, 81–88. 10.1210/en.2013-1738 24169555 PMC3868808

[B72] Chemelli R. M. Willie J. T. Sinton C. M. Elmquist J. K. Scammell T. Lee C.. (1999). Narcolepsy in orexin knockout mice: molecular genetics of sleep regulation. Cell 98, 437–451. 10.1016/s0092-8674(00)81973-x 10481909

[B73] Chen Y. Hu C. Hsu C. K. Zhang Q. Bi C. Asnicar M.. (2002). Targeted disruption of the melanin-concentrating hormone receptor-1 results in hyperphagia and resistance to diet-induced obesity. Endocrinology 143, 2469–2477. 10.1210/en.143.7.2469 12072376

[B74] Chen X. W. Mu Y. Huang H. P. Guo N. Zhang B. Fan S. Y.. (2008). Hypocretin-1 potentiates NMDA receptor-mediated somatodendritic secretion from locus ceruleus neurons. J. Neurosci. 28, 3202–3208. 10.1523/jneurosci.4426-07.2008 18354023 PMC6670716

[B75] Chen M. Woods S. C. Porte D. Jr. (1975). Effect of cerebral intraventricular insulin on pancreatic insulin secretion in the dog. Diabetes 24, 910–914. 10.2337/diabetes.24.10.910 1100460

[B76] Choi D. L. Davis J. F. Fitzgerald M. E. Benoit S. C. (2010). The role of orexin-A in food motivation, reward-based feeding behavior and food-induced neuronal activation in rats. Neuroscience 167, 11–20. 10.1016/j.neuroscience.2010.02.002 20149847

[B77] Chou T. C. Lee C. E. Lu J. Elmquist J. K. Hara J. Willie J. T.. (2001). Orexin (hypocretin) neurons contain dynorphin. J. Neurosci. 21:RC168. 11567079 10.1523/JNEUROSCI.21-19-j0003.2001 PMC6762880

[B78] Chua S. C. Jr. Chung W. K. Wu-Peng X. S. Zhang Y. Liu S. M. Tartaglia L.. (1996). Phenotypes of mouse diabetes and rat fatty due to mutations in the OB (leptin) receptor. Science 271, 994–996. 10.1126/science.271.5251.994 8584938

[B79] Chung S. Hopf F. W. Nagasaki H. Li C. Y. Belluzzi J. D. Bonci A.. (2009). The melanin-concentrating hormone system modulates cocaine reward. Proc. Natl. Acad. Sci. U S A 106, 6772–6777. 10.1073/pnas.0811331106 19342492 PMC2672513

[B80] Clegg D. J. Air E. L. Benoit S. C. Sakai R. S. Seeley R. J. Woods S. C. (2003). Intraventricular melanin-concentrating hormone stimulates water intake independent of food intake. Am. J. Physiol. Regul. Integr. Comp. Physiol. 284, R494–R499. 10.1152/ajpregu.00399.2002 12557891

[B81] Clegg D. J. Air E. L. Woods S. C. Seeley R. J. (2002). Eating elicited by orexin-a, but not melanin-concentrating hormone, is opioid mediated. Endocrinology 143, 2995–3000. 10.1210/en.143.8.2995 12130565

[B82] Clement O. Sapin E. Libourel P. A. Arthaud S. Brischoux F. Fort P.. (2012). The lateral hypothalamic area controls paradoxical (REM) sleep by means of descending projections to brainstem GABAergic neurons. J. Neurosci. 32, 16763–16774. 10.1523/jneurosci.1885-12.2012 23175830 PMC6621764

[B83] Clineschmidt B. V. Mcguffin J. C. Bunting P. B. (1979). Neurotensin: antinocisponsive action in rodents. Eur. J. Pharmacol. 54, 129–139. 10.1016/0014-2999(79)90415-1 421735

[B84] Cohen P. Zhao C. Cai X. Montez J. M. Rohani S. C. Feinstein P.. (2001). Selective deletion of leptin receptor in neurons leads to obesity. J. Clin. Invest. 108, 1113–1121. 10.1172/jci200113914 11602618 PMC209535

[B85] Cone J. J. Mccutcheon J. E. Roitman M. F. (2014). Ghrelin acts as an interface between physiological state and phasic dopamine signaling. J. Neurosci. 34, 4905–4913. 10.1523/jneurosci.4404-13.2014 24695709 PMC3972717

[B86] Cristino L. Busetto G. Imperatore R. Ferrandino I. Palomba L. Silvestri C.. (2013). Obesity-driven synaptic remodeling affects endocannabinoid control of orexinergic neurons. Proc. Natl. Acad. Sci. U S A 110, E2229–E2238. 10.1073/pnas.1219485110 23630288 PMC3683753

[B87] Crocker A. España R. A. Papadopoulou M. Saper C. B. Faraco J. Sakurai T.. (2005). Concomitant loss of dynorphin, NARP and orexin in narcolepsy. Neurology 65, 1184–1188. 10.1212/01.wnl.0000168173.71940.ab 16247044 PMC2254145

[B88] Cui H. Cai F. Belsham D. D. (2006). Leptin signaling in neurotensin neurons involves STAT, MAP kinases ERK1/2 and p38 through c-Fos and ATF1. Faseb J. 20, 2654–2656. 10.1096/fj.06-5989fje 17077290

[B89] Cui H. Sohn J. W. Gautron L. Funahashi H. Williams K. W. Elmquist J. K.. (2012). Neuroanatomy of melanocortin-4 receptor pathway in the lateral hypothalamic area. J. Comp. Neurol. 520, 4168–4183. 10.1002/cne.23145 22605619 PMC3652326

[B90] Cvetkovic V. Brischoux F. Jacquemard C. Fellmann D. Griffond B. Risold P. Y. (2004). Characterization of subpopulations of neurons producing melanin-concentrating hormone in the rat ventral diencephalon. J. Neurochem. 91, 911–919. 10.1111/j.1471-4159.2004.02776.x 15525345

[B91] Dalal M. A. Schuld A. Haack M. Uhr M. Geisler P. Eisensehr I.. (2001). Normal plasma levels of orexin A (hypocretin-1) in narcoleptic patients. Neurology 56, 1749–1751. 10.1212/wnl.56.12.1749 11425946

[B92] Date Y. Ueta Y. Yamashita H. Yamaguchi H. Matsukura S. Kangawa K.. (1999). Orexins, orexigenic hypothalamic peptides, interact with autonomic, neuroendocrine and neuroregulatory systems. Proc. Natl. Acad. Sci. U S A 96, 748–753. 10.1073/pnas.96.2.748 9892705 PMC15208

[B93] Dayyeh B. K. Lautz D. B. Thompson C. C. (2010). Gastrojejunal stoma diameter predicts weight regain after Roux-en-Y gastric bypass. Clin. Gastroenterol. Hepatol. 9, 228–233. 10.1016/j.cgh.2010.11.004 21092760 PMC3043151

[B94] de Lecea L. Kilduff T. S. Peyron C. Gao X. Foye P. E. Danielson P. E.. (1998). The hypocretins: hypothalamus-specific peptides with neuroexcitatory activity. Proc. Natl. Acad. Sci. U S A 95, 322–327. 10.1073/pnas.95.1.322 9419374 PMC18213

[B95] Delgado J. M. Anand B. K. (1953). Increase of food intake induced by electrical stimulation of the lateral hypothalamus. Am. J. Physiol. 172, 162–168. 13030733 10.1152/ajplegacy.1952.172.1.162

[B96] Deutch A. Y. Bean A. J. Bissette G. Nemeroff C. B. Robbins R. J. Roth R. H. (1987). Stress-induced alterations in neurotensin, somatostatin and corticotropin-releasing factor in mesotelencephalic dopamine system regions. Brain Res. 417, 350–354. 10.1016/0006-8993(87)90462-8 2888514

[B97] Diano S. Horvath B. Urbanski H. F. Sotonyi P. Horvath T. L. (2003). Fasting activates the nonhuman primate hypocretin (orexin) system and its postsynaptic targets. Endocrinology 144, 3774–3778. 10.1210/en.2003-0274 12933647

[B98] Diniz Behn C. G. Klerman E. B. Mochizuki T. Lin S. C. Scammell T. E. (2010). Abnormal sleep/wake dynamics in orexin knockout mice. Sleep 33, 297–306. 20337187 10.1093/sleep/33.3.297 PMC2831423

[B99] Domingos A. I. Sordillo A. Dietrich M. O. Liu Z. W. Tellez L. A. Vaynshteyn J.. (2013). Hypothalamic melanin concentrating hormone neurons communicate the nutrient value of sugar. Elife 2:e01462. 10.7554/elife.01462 24381247 PMC3875383

[B100] Dubuc I. Sarret P. Labbé-Jullié C. Botto J. M. Honoré E. Bourdel E.. (1999). Identification of the receptor subtype involved in the analgesic effect of neurotensin. J. Neurosci. 19, 503–510. 9870978 10.1523/JNEUROSCI.19-01-00503.1999 PMC6782393

[B101] Duncan E. A. Proulx K. Woods S. C. (2005). Central administration of melanin-concentrating hormone increases alcohol and sucrose/quinine intake in rats. Alcohol. Clin. Exp. Res. 29, 958–964. 10.1097/01.alc.0000167741.42353.10 15976521

[B102] Dundas B. Harris M. Narasimhan M. (2007). Psychogenic polydipsia review: etiology, differential and treatment. Curr. Psychiatry Rep. 9, 236–241. 10.1007/s11920-007-0025-7 17521521

[B103] Dunnett S. B. Lane D. M. Winn P. (1985). Ibotenic acid lesions of the lateral hypothalamus: comparison with 6-hydroxydopamine-induced sensorimotor deficits. Neuroscience 14, 509–518. 10.1016/0306-4522(85)90306-9 3921869

[B104] Edwards C. M. Abusnana S. Sunter D. Murphy K. G. Ghatei M. A. Bloom S. R. (1999). The effect of the orexins on food intake: comparison with neuropeptide Y, melanin-concentrating hormone and galanin. J. Endocrinol. 160, R7–R12. 10.1677/joe.0.160r007 10077743

[B105] Eggermann E. Bayer L. Serafin M. Saint-Mleux B. Bernheim L. Machard D.. (2003). The wake-promoting hypocretin-orexin neurons are in an intrinsic state of membrane depolarization. J. Neurosci. 23, 1557–1562. 12629156 10.1523/JNEUROSCI.23-05-01557.2003 PMC6741978

[B106] Elias C. F. Lee C. E. Kelly J. F. Ahima R. S. Kuhar M. Saper C. B.. (2001). Characterization of CART neurons in the rat and human hypothalamus. J. Comp. Neurol. 432, 1–19. 10.1002/cne.1085 11241374

[B107] Elias C. F. Saper C. B. Maratos-Flier E. Tritos N. A. Lee C. Kelly J.. (1998). Chemically defined projections linking the mediobasal hypothalamus and the lateral hypothalamic area. J. Comp. Neurol. 402, 442–459. 10.1002/(sici)1096-9861(19981228)402:4<442::aid-cne2>3.3.co;2-i 9862320

[B108] Elliott P. J. Chan J. Parker Y. M. Nemeroff C. B. (1986). Behavioral effects of neurotensin in the open field: structure-activity studies. Brain Res. 381, 259–265. 10.1016/0006-8993(86)90075-2 3756503

[B109] Elliott P. J. Nemeroff C. B. (1986). Repeated neurotensin administration in the ventral tegmental area: effects on baseline and D-amphetamine-induced locomotor activity. Neurosci. Lett. 68, 239–244. 10.1016/0304-3940(86)90149-7 3748452

[B110] Eriksson K. S. Sergeeva O. Brown R. E. Haas H. L. (2001). Orexin/hypocretin excites the histaminergic neurons of the tuberomammillary nucleus. J. Neurosci. 21, 9273–9279. 11717361 10.1523/JNEUROSCI.21-23-09273.2001 PMC6763926

[B111] Ervin G. N. Birkemo L. S. Nemeroff C. B. Prange A. J. Jr. (1981). Neurotensin blocks certain amphetamine-induced behaviours. Nature 291, 73–76. 10.1038/291073a0 7231526

[B112] España R. A. Melchior J. R. Roberts D. C. Jones S. R. (2011). Hypocretin 1/orexin A in the ventral tegmental area enhances dopamine responses to cocaine and promotes cocaine self-administration. Psychopharmacology (Berl) 214, 415–426. 10.1007/s00213-010-2048-8 20959967 PMC3085140

[B113] España R. A. Oleson E. B. Locke J. L. Brookshire B. R. Roberts D. C. Jones S. R. (2010). The hypocretin-orexin system regulates cocaine self-administration via actions on the mesolimbic dopamine system. Eur J. Neurosci. 31, 336–348. 10.1111/j.1460-9568.2009.07065.x 20039943 PMC2881680

[B114] España R. A. Plahn S. Berridge C. W. (2002). Circadian-dependent and circadian-independent behavioral actions of hypocretin/orexin. Brain Res. 943, 224–236. 10.1016/s0006-8993(02)02653-7 12101045

[B115] Estabrooke I. V. Mccarthy M. T. Ko E. Chou T. C. Chemelli R. M. Yanagisawa M.. (2001). Fos expression in orexin neurons varies with behavioral state. J. Neurosci. 21, 1656–1662. 11222656 10.1523/JNEUROSCI.21-05-01656.2001 PMC6762959

[B116] Fadel J. Deutch A. Y. (2002). Anatomical substrates of orexin-dopamine interactions: lateral hypothalamic projections to the ventral tegmental area. Neuroscience 111, 379–387. 10.1016/s0306-4522(02)00017-9 11983323

[B117] Fang F. G. Moreau J. L. Fields H. L. (1987). Dose-dependent antinociceptive action of neurotensin microinjected into the rostroventromedial medulla of the rat. Brain Res. 420, 171–174. 10.1016/0006-8993(87)90255-1 3676752

[B118] Farkas R. H. Chien P. Y. Nakajima S. Nakajima Y. (1997). Neurotensin and dopamine D2 activation oppositely regulate the same K+ conductance in rat midbrain dopaminergic neurons. Neurosci. Lett. 231, 21–24. 10.1016/s0304-3940(97)00530-2 9280158

[B119] Farooqi I. S. Jebb S. A. Langmack G. Lawrence E. Cheetham C. H. Prentice A. M.. (1999). Effects of recombinant leptin therapy in a child with congenital leptin deficiency. N. Engl. J. Med. 341, 879–884. 10.1056/nejm199909163411204 10486419

[B120] Feifel D. Goldenberg J. Melendez G. Shilling P. D. (2010). The acute and subchronic effects of a brain-penetrating, neurotensin-1 receptor agonist on feeding, body weight and temperature. Neuropharmacology 58, 195–198. 10.1016/j.neuropharm.2009.07.001 19596358 PMC2784133

[B121] Fekete C. Wittmann G. Liposits Z. Lechan R. M. (2004). Origin of cocaine- and amphetamine-regulated transcript (CART)-immunoreactive innervation of the hypothalamic paraventricular nucleus. J. Comp. Neurol. 469, 340–350. 10.1002/cne.10999 14730586

[B122] Feurle G. E. (1998). Xenin–a review. Peptides 19, 609–615. 10.1016/s0196-9781(97)00378-1 9533652

[B123] Flegal K. M. Carroll M. D. Kit B. K. Ogden C. L. (2012). Prevalence of obesity and trends in the distribution of body mass index among US adults, 1999–2010. JAMA 307, 491–497. 10.1001/jama.2012.39 22253363

[B124] Foo K. S. Brismar H. Broberger C. (2008). Distribution and neuropeptide coexistence of nucleobindin-2 mRNA/nesfatin-like immunoreactivity in the rat CNS. Neuroscience 156, 563–579. 10.1016/j.neuroscience.2008.07.054 18761059

[B125] Fukunaka Y. Shinkai T. Hwang R. Hori H. Utsunomiya K. Sakata S.. (2007). The orexin 1 receptor (HCRTR1) gene as a susceptibility gene contributing to polydipsia-hyponatremia in schizophrenia. Neuromolecular Med. 9, 292–297. 10.1007/s12017-007-8001-2 17999203

[B126] Funato H. Tsai A. L. Willie J. T. Kisanuki Y. Williams S. C. Sakurai T.. (2009). Enhanced orexin receptor-2 signaling prevents diet-induced obesity and improves leptin sensitivity. Cell Metab. 9, 64–76. 10.1016/j.cmet.2008.10.010 19117547 PMC2630400

[B127] Furutani N. Hondo M. Kageyama H. Tsujino N. Mieda M. Yanagisawa M.. (2013). Neurotensin co-expressed in orexin-producing neurons in the lateral hypothalamus plays an important role in regulation of sleep/wakefulness States. PLoS One 8:e62391. 10.1371/journal.pone.0062391 23620827 PMC3631195

[B128] Gaykema R. P. Goehler L. E. (2009). Lipopolysaccharide challenge-induced suppression of Fos in hypothalamic orexin neurons: their potential role in sickness behavior. Brain Behav. Immun. 23, 926–930. 10.1016/j.bbi.2009.03.005 19328847 PMC2792632

[B129] Georgescu D. Sears R. M. Hommel J. D. Barrot M. Bolaños C. A. Marsh D. J.. (2005). The hypothalamic neuropeptide melanin-concentrating hormone acts in the nucleus accumbens to modulate feeding behavior and forced-swim performance. J. Neurosci. 25, 2933–2940. 10.1523/jneurosci.1714-04.2005 15772353 PMC6725126

[B130] Gerashchenko D. Kohls M. D. Greco M. Waleh N. S. Salin-Pascual R. Kilduff T. S.. (2001). Hypocretin-2-saporin lesions of the lateral hypothalamus produce narcoleptic-like sleep behavior in the rat. J. Neurosci. 21, 7273–7283. 11549737 10.1523/JNEUROSCI.21-18-07273.2001 PMC6762996

[B131] Glick M. Segal-Lieberman G. Cohen R. Kronfeld-Schor N. (2009). Chronic MCH infusion causes a decrease in energy expenditure and body temperature and an increase in serum IGF-1 levels in mice. Endocrine 36, 479–485. 10.1007/s12020-009-9252-5 19859841

[B132] Glimcher P. W. Giovino A. A. Hoebel B. G. (1987). Neurotensin self-injection in the ventral tegmental area. Brain Res. 403, 147–150. 10.1016/0006-8993(87)90134-x 3828807

[B133] Glimcher P. W. Margolin D. H. Giovino A. A. Hoebel B. G. (1984). Neurotensin: a new ’reward peptide’. Brain Res. 291, 119–124. 10.1016/0006-8993(84)90657-7 6320951

[B134] Goforth P. B. Leinninger G. M. Patterson C. M. Satin L. S. Myers M. G. Jr. (2014). Leptin acts via lateral hypothalamic area neurotensin neurons to inhibit orexin neurons by multiple GABA-independent mechanisms. J. Neurosci. 34, 11405–11415. 10.1523/jneurosci.5167-13.2014 25143620 PMC4138347

[B135] Goto M. Canteras N. S. Burns G. Swanson L. W. (2005). Projections from the subfornical region of the lateral hypothalamic area. J. Comp. Neurol. 493, 412–438. 10.1002/cne.20764 16261534 PMC2844126

[B136] Grabauskas G. Moises H. C. (2003). Gastrointestinal-projecting neurones in the dorsal motor nucleus of the vagus exhibit direct and viscerotopically organized sensitivity to orexin. J. Physiol. 549, 37–56. 10.1113/jphysiol.2002.029546 12679367 PMC2342920

[B137] Griffiths E. C. Slater P. Widdowson P. S. (1986). The hypothermic action of carbachol in the rat brain periaqueductal grey area may involve neurotensin. Br. J. Pharmacol. 88, 653–658. 10.1111/j.1476-5381.1986.tb10247.x 3742153 PMC1916974

[B138] Grossberg A. J. Zhu X. Leinninger G. M. Levasseur P. R. Braun T. P. Myers M. G.. (2011). Inflammation-induced lethargy is mediated by suppression of orexin neuron activity. J. Neurosci. 31, 11376–11386. 10.1523/jneurosci.2311-11.2011 21813697 PMC3155688

[B139] Grossman S. P. Dacey D. Halaris A. E. Collier T. Routtenberg A. (1978). Aphagia and adipsia after preferential destruction of nerve cell bodies in hypothalamus. Science 202, 537–539. 10.1126/science.705344 705344

[B140] Gully D. Canton M. Boigegrain R. Jeanjean F. Molimard J. C. Poncelet M.. (1993). Biochemical and pharmacological profile of a potent and selective nonpeptide antagonist of the neurotensin receptor. Proc. Natl. Acad. Sci. U S A 90, 65–69. 10.1073/pnas.90.1.65 8380498 PMC45600

[B141] Hagan J. J. Leslie R. A. Patel S. Evans M. L. Wattam T. A. Holmes S.. (1999). Orexin A activates locus coeruleus cell firing and increases arousal in the rat. Proc. Natl. Acad. Sci. U S A 96, 10911–10916. 10.1073/pnas.96.19.10911 10485925 PMC17982

[B142] Hahn J. D. Swanson L. W. (2010). Distinct patterns of neuronal inputs and outputs of the juxtaparaventricular and suprafornical regions of the lateral hypothalamic area in the male rat. Brain Res. Rev. 64, 14–103. 10.1016/j.brainresrev.2010.02.002 20170674 PMC2886810

[B143] Håkansson M. de Lecea L. Sutcliffe J. G. Yanagisawa M. Meister B. (1999). Leptin receptor- and STAT3-immunoreactivities in hypocretin/orexin neurones of the lateral hypothalamus. J. Neuroendocrinol. 11, 653–663. 10.1046/j.1365-2826.1999.00378.x 10447804

[B144] Halaas J. L. Gajiwala K. S. Maffei M. Cohen S. L. Chait B. T. Rabinowitz D.. (1995). Weight-reducing effects of the plasma protein encoded by the obese gene. Science 269, 543–546. 10.1126/science.7624777 7624777

[B145] Hara J. Beuckmann C. T. Nambu T. Willie J. T. Chemelli R. M. Sinton C. M.. (2001). Genetic ablation of orexin neurons in mice results in narcolepsy, hypophagia and obesity. Neuron 30, 345–354. 10.1016/s0896-6273(01)00293-8 11394998

[B146] Harris G. C. Aston-Jones G. (2006). Arousal and reward: a dichotomy in orexin function. Trends Neurosci. 29, 571–577. 10.1016/j.tins.2006.08.002 16904760

[B147] Harris G. C. Wimmer M. Aston-Jones G. (2005). A role for lateral hypothalamic orexin neurons in reward seeking. Nature 437, 556–559. 10.1038/nature04071 16100511

[B148] Hart S. Franklin R. C. Russell J. Abraham S. (2013). A review of feeding methods used in the treatment of anorexia nervosa. J. Eat. Disord. 1:36. 10.1186/2050-2974-1-36 24999415 PMC4081821

[B149] Harthoorn L. F. Sañé A. Nethe M. Van Heerikhuize J. J. (2005). Multi-transcriptional profiling of melanin-concentrating hormone and orexin-containing neurons. Cell. Mol. Neurobiol. 25, 1209–1223. 10.1007/s10571-005-8184-8 16388333 PMC11529226

[B150] Hasegawa E. Yanagisawa M. Sakurai T. Mieda M. (2014). Orexin neurons suppress narcolepsy via 2 distinct efferent pathways. J. Clin. Invest. 124, 604–616. 10.1172/jci71017 24382351 PMC3904620

[B151] Hassani O. K. Henny P. Lee M. G. Jones B. E. (2010). GABAergic neurons intermingled with orexin and MCH neurons in the lateral hypothalamus discharge maximally during sleep. Eur J. Neurosci. 32, 448–457. 10.1111/j.1460-9568.2010.07295.x 20597977 PMC2921479

[B152] Hassani O. K. Lee M. G. Jones B. E. (2009). Melanin-concentrating hormone neurons discharge in a reciprocal manner to orexin neurons across the sleep-wake cycle. Proc. Natl. Acad. Sci. U S A 106, 2418–2422. 10.1073/pnas.0811400106 19188611 PMC2650171

[B153] Hawes B. E. Kil E. Green B. O’neill K. Fried S. Graziano M. P. (2000). The melanin-concentrating hormone receptor couples to multiple G proteins to activate diverse intracellular signaling pathways. Endocrinology 141, 4524–4532. 10.1210/en.141.12.4524 11108264

[B154] Hawken E. R. Crookall J. M. Reddick D. Millson R. C. Milev R. Delva N. (2009). Mortality over a 20-year period in patients with primary polydipsia associated with schizophrenia: a retrospective study. Schizophr. Res. 107, 128–133. 10.1016/j.schres.2008.09.029 18984069

[B155] Hawkins M. F. (1986a). Aphagia in the rat following microinjection of neurotensin into the ventral tegmental area. Life Sci. 38, 2383–2388. 10.1016/0024-3205(86)90606-5 3459947

[B156] Hawkins M. F. (1986b). Central nervous system neurotensin and feeding. Physiol. Behav. 36, 1–8. 10.1016/0031-9384(86)90064-8 3952166

[B157] Hawkins M. F. Barkemeyer C. A. Tulley R. T. (1986). Synergistic effects of dopamine agonists and centrally administered neurotensin on feeding. Pharmacol. Biochem. Behav. 24, 1195–1201. 10.1016/0091-3057(86)90170-x 3725825

[B158] Haynes A. C. Jackson B. Chapman H. Tadayyon M. Johns A. Porter R. A.. (2000). A selective orexin-1 receptor antagonist reduces food consumption in male and female rats. Regul. Pept. 96, 45–51. 10.1016/s0167-0115(00)00199-3 11102651

[B159] Haynes A. C. Jackson B. Overend P. Buckingham R. E. Wilson S. Tadayyon M.. (1999). Effects of single and chronic intracerebroventricular administration of the orexins on feeding in the rat. Peptides 20, 1099–1105. 10.1016/s0196-9781(99)00105-9 10499428

[B161] Hermans E. Maloteaux J. M. Octave J. N. (1992). Phospholipase C activation by neurotensin and neuromedin N in Chinese hamster ovary cells expressing the rat neurotensin receptor. Brain Res. Mol. Brain Res. 15, 332–338. 10.1016/0169-328x(92)90126-v 1331689

[B162] Hernandez L. Hoebel B. G. (1988). Feeding and hypothalamic stimulation increase dopamine turnover in the accumbens. Physiol. Behav. 44, 599–606. 10.1016/0031-9384(88)90324-1 3237847

[B163] Herve D. Tassin J. P. Studler J. M. Dana C. Kitabgi P. Vincent J. P.. (1986). Dopaminergic control of 125I-labeled neurotensin binding site density in corticolimbic structures of the rat brain. Proc. Natl. Acad. Sci. U S A 83, 6203–6207. 10.1073/pnas.83.16.6203 3016745 PMC386468

[B164] Hetherington A. W. Ranson S. W. (1939). Experimental hyothamico-hypophyseal obesity in the rat. Exp. Biol. Med. 41, 465–466 10.3181/00379727-41-10711p

[B160] Hetherington A. W. Ranson S. W. (1940). Hypothalamic lesions and adiposity in the rat. Anatomical Record 78, 149–172 10.1002/ar.1090780203

[B165] Heydendael W. Sharma K. Iyer V. Luz S. Piel D. Beck S.. (2011). Orexins/hypocretins act in the posterior paraventricular thalamic nucleus during repeated stress to regulate facilitation to novel stress. Endocrinology 152, 4738–4752. 10.1210/en.2011-1652 21971160 PMC3230061

[B166] Higgins G. A. Hoffman G. E. Wray S. Schwaber J. S. (1984). Distribution of neurotensin-immunoreactivity within baroreceptive portions of the nucleus of the tractus solitarius and the dorsal vagal nucleus of the rat. J. Comp. Neurol. 226, 155–164. 10.1002/cne.902260202 6376547

[B167] Hoang Q. V. Bajic D. Yanagisawa M. Nakajima S. Nakajima Y. (2003). Effects of orexin (hypocretin) on GIRK channels. J. Neurophysiol. 90, 693–702. 10.1152/jn.00001.2003 12702704

[B168] Hoebel B. G. (1965). Hypothalamic lesions by electrocauterization: disinhibition of feeding and self-stimulation. Science 149, 452–453. 10.1126/science.149.3682.452 17809421

[B169] Hoebel B. G. Teitelbaum P. (1962). Hypothalamic control of feeding and self-stimulation. Science 135, 375–377. 10.1126/science.135.3501.375 13907995

[B170] Holmqvist T. Johansson L. Ostman M. Ammoun S. Akerman K. E. Kukkonen J. P. (2005). OX1 orexin receptors couple to adenylyl cyclase regulation via multiple mechanisms. J. Biol. Chem. 280, 6570–6579. 10.1074/jbc.m407397200 15611118

[B171] Honda Y. Doi Y. Ninomiya R. Ninomiya C. (1986). Increased frequency of non-insulin-dependent diabetes mellitus among narcoleptic patients. Sleep 9, 254–259. 3518018 10.1093/sleep/9.1.254

[B172] Honda M. Eriksson K. S. Zhang S. Tanaka S. Lin L. Salehi A.. (2009). IGFBP3 colocalizes with and regulates hypocretin (orexin). PLoS One 4:e4254. 10.1371/journal.pone.0004254 19158946 PMC2617764

[B173] Horvath T. L. Diano S. van den Pol A. N. (1999a). Synaptic interaction between hypocretin (orexin) and neuropeptide Y cells in the rodent and primate hypothalamus: a novel circuit implicated in metabolic and endocrine regulations. J. Neurosci. 19, 1072–1087. 9920670 10.1523/JNEUROSCI.19-03-01072.1999 PMC6782143

[B174] Horvath T. L. Gao X. B. (2005). Input organization and plasticity of hypocretin neurons: possible clues to obesity’s association with insomnia. Cell Metab. 1, 279–286. 10.1016/j.cmet.2005.03.003 16054072

[B175] Horvath T. L. Peyron C. Diano S. Ivanov A. Aston-Jones G. Kilduff T. S.. (1999b). Hypocretin (orexin) activation and synaptic innervation of the locus coeruleus noradrenergic system. J. Comp. Neurol. 415, 145–159. 10.1002/(sici)1096-9861(19991213)415:2<145::aid-cne1>3.3.co;2-u 10545156

[B176] Hou I. C. Suzuki C. Kanegawa N. Oda A. Yamada A. Yoshikawa M.. (2011). beta-Lactotensin derived from bovine beta-lactoglobulin exhibits anxiolytic-like activity as an agonist for neurotensin NTS(2) receptor via activation of dopamine D(1) receptor in mice. J. Neurochem. 119, 785–790. 10.1111/j.1471-4159.2011.07472.x 21895659

[B177] Huang Z. L. Qu W. M. Li W. D. Mochizuki T. Eguchi N. Watanabe T.. (2001). Arousal effect of orexin A depends on activation of the histaminergic system. Proc. Natl. Acad. Sci. U S A 98, 9965–9970. 10.1073/pnas.181330998 11493714 PMC55561

[B178] Hwang J. R. Baek M. W. Sim J. Choi H. S. Han J. M. Kim Y. L.. (2010). Intermolecular cross-talk between NTR1 and NTR2 neurotensin receptor promotes intracellular sequestration and functional inhibition of NTR1 receptors. Biochem. Biophys. Res. Commun. 391, 1007–1013. 10.1016/j.bbrc.2009.12.007 19968961

[B179] Ida T. Nakahara K. Katayama T. Murakami N. Nakazato M. (1999). Effect of lateral cerebroventricular injection of the appetite-stimulating neuropeptide, orexin and neuropeptide Y, on the various behavioral activities of rats. Brain Res. 821, 526–529. 10.1016/s0006-8993(99)01131-2 10064841

[B180] Iftene F. Bowie C. Milev R. Hawken E. Talikowska-Szymczak E. Potopsingh D.. (2013). Identification of primary polydipsia in a severe and persistent mental illness outpatient population: a prospective observational study. Psychiatry Res. 210, 679–683. 10.1016/j.psychres.2013.04.011 23810384

[B181] Inutsuka A. Inui A. Tabuchi S. Tsunematsu T. Lazarus M. Yamanaka A. (2014). Concurrent and robust regulation of feeding behaviors and metabolism by orexin neurons. Neuropharmacology 85C, 451–460. 10.1016/j.neuropharm.2014.06.015 24951857

[B182] Iversen L. L. Iversen S. D. Bloom F. Douglas C. Brown M. Vale W. (1978). Calcium-dependent release of somatostatin and neurotensin from rat brain in vitro. Nature 273, 161–163. 10.1038/273161a0 643079

[B183] Jego S. Glasgow S. D. Herrera C. G. Ekstrand M. Reed S. J. Boyce R.. (2013). Optogenetic identification of a rapid eye movement sleep modulatory circuit in the hypothalamus. Nat. Neurosci. 16, 1637–1643. 10.1038/nn.3522 24056699 PMC4974078

[B184] Jensen M. D. Ryan D. H. Apovian C. M. Ard J. D. Comuzzie A. G. Donato K. A.. (2014). 2013 AHA/ACC/TOS guideline for the management of overweight and obesity in adults: a report of the American College of Cardiology/American Heart Association Task Force on Practice Guidelines and The Obesity Society. Circulation 129, S102–S138. 10.1161/01.cir.0000437739.71477.ee 24222017 PMC5819889

[B185] Jeon J. Y. Bradley R. L. Kokkotou E. G. Marino F. E. Wang X. Pissios P.. (2006). MCH−/− mice are resistant to aging-associated increases in body weight and insulin resistance. Diabetes 55, 428–434. 10.2337/diabetes.55.02.06.db05-0203 16443777

[B186] Johnson P. L. Samuels B. C. Fitz S. D. Lightman S. L. Lowry C. A. Shekhar A. (2012). Activation of the orexin 1 receptor is a critical component of CO2-mediated anxiety and hypertension but not bradycardia. Neuropsychopharmacology 37, 1911–1922. 10.1038/npp.2012.38 22453138 PMC3376323

[B187] Johnson P. L. Truitt W. Fitz S. D. Minick P. E. Dietrich A. Sanghani S.. (2010). A key role for orexin in panic anxiety. Nat. Med. 16, 111–115. 10.1038/nm.2075 20037593 PMC2832844

[B188] Jolicoeur F. B. Rivest R. St-Pierre S. Gagné M. A. Dumais M. (1985). The effects of neurotensin and [D-Tyr11]-NT on the hyperactivity induced by intra-accumbens administration of a potent dopamine receptor agonist. Neuropeptides 6, 143–156. 10.1016/0143-4179(85)90105-2 2987745

[B189] Kahn D. Hou-Yu A. Zimmerman E. A. (1982). Localization of neurotensin in the hypothalamus. Ann. N Y Acad. Sci. 400, 117–131. 10.1111/j.1749-6632.1982.tb31564.x 6132575

[B190] Kalivas P. W. Burgess S. K. Nemeroff C. B. Prange A. J. Jr. (1983). Behavioral and neurochemical effects of neurotensin microinjection into the ventral tegmental area of the rat. Neuroscience 8, 495–505. 10.1016/0306-4522(83)90195-1 6406930

[B191] Kalivas P. W. Duffy P. (1990). Effect of acute and daily neurotensin and enkephalin treatments on extracellular dopamine in the nucleus accumbens. J. Neurosci. 10, 2940–2949. 10.1002/syn.890050104 1697899 PMC6570231

[B192] Kalivas P. W. Jennes L. Miller J. S. (1985a). A catecholaminergic projection from the ventral tegmental area to the diagonal band of Broca: modulation by neurotensin. Brain Res. 326, 229–238. 10.1016/0006-8993(85)90032-0 2857589

[B193] Kalivas P. W. Nemeroff C. B. Prange A. J. Jr. (1981). Increase in spontaneous motor activity following infusion of neurotensin into the ventral tegmental area. Brain Res. 229, 525–529. 10.1016/0006-8993(81)91016-7 7306825

[B194] Kalivas P. W. Nemeroff C. B. Prange A. J. Jr. (1982). Neuroanatomical sites of action of neurotensin. Ann. N Y Acad. Sci. 400, 307–318. 10.1111/j.1749-6632.1982.tb31577.x 6963113

[B195] Kalivas P. W. Nemeroff C. B. Miller J. S. Prange A. J. Jr. (1985b). Microinjection of neurotensin into the ventral tegmental area produces hypothermia: evaluation of dopaminergic mediation. Brain Res. 326, 219–227. 10.1016/0006-8993(85)90031-9 2982460

[B196] Kalivas P. W. Taylor S. (1985). Behavioral and neurochemical effect of daily injection with neurotensin into the ventral tegmental area. Brain Res. 358, 70–76. 10.1016/0006-8993(85)90949-7 4075132

[B197] Kantor S. Mochizuki T. Lops S. N. Ko B. Clain E. Clark E.. (2013). Orexin gene therapy restores the timing and maintenance of wakefulness in narcoleptic mice. Sleep 36, 1129–1138. 10.5665/sleep.2870 23904672 PMC3700709

[B198] Karnani M. M. Apergis-Schoute J. Adamantidis A. Jensen L. T. De Lecea L. Fugger L.. (2011). Activation of central orexin/hypocretin neurons by dietary amino acids. Neuron 72, 616–629. 10.1016/j.neuron.2011.08.027 22099463

[B199] Karnani M. M. Szabó G. Erdélyi F. Burdakov D. (2013). Lateral hypothalamic GAD65 neurons are spontaneously firing and distinct from orexin- and melanin-concentrating hormone neurons. J. Physiol. 591, 933–953. 10.1113/jphysiol.2012.243493 23184514 PMC3591707

[B200] Kawai Y. Takagi H. Tohyama M. (1988). Co-localization of neurotensin- and cholecystokinin-like immunoreactivities in catecholamine neurons in the rat dorsomedial medulla. Neuroscience 24, 227–236. 10.1016/0306-4522(88)90326-0 2897090

[B201] Kawauchi H. Kawazoe I. Tsubokawa M. Kishida M. Baker B. I. (1983). Characterization of melanin-concentrating hormone in chum salmon pituitaries. Nature 305, 321–323. 10.1038/305321a0 6621686

[B202] Kaye W. H. Wierenga C. E. Bailer U. F. Simmons A. N. Bischoff-Grethe A. (2013). Nothing tastes as good as skinny feels: the neurobiology of anorexia nervosa. Trends Neurosci. 36, 110–120. 10.1016/j.tins.2013.01.003 23333342 PMC3880159

[B203] Kempadoo K. A. Tourino C. Cho S. L. Magnani F. Leinninger G. M. Stuber G. D.. (2013). Hypothalamic neurotensin projections promote reward by enhancing glutamate transmission in the VTA. J. Neurosci. 33, 7618–7626. 10.1523/jneurosci.2588-12.2013 23637156 PMC3865559

[B204] Kenny P. J. (2011). Reward mechanisms in obesity: new insights and future directions. Neuron 69, 664–679. 10.1016/j.neuron.2011.02.016 21338878 PMC3057652

[B206] Kessler J. P. Moyse E. Kitabgi P. Vincent J. P. Beaudet A. (1987). Distribution of neurotensin binding sites in the caudal brainstem of the rat: a light microscopic radioautographic study. Neuroscience 23, 189–198. 10.1016/0306-4522(87)90282-x 3683860

[B205] Kessler B. A. Stanley E. M. Frederick-Duus D. Fadel J. (2011). Age-related loss of orexin/hypocretin neurons. Neuroscience 178, 82–88. 10.1016/j.neuroscience.2011.01.031 21262323 PMC3048917

[B207] Kim E. R. Leckstrom A. Mizuno T. M. (2008). Impaired anorectic effect of leptin in neurotensin receptor 1-deficient mice. Behav. Brain Res. 194, 66–71. 10.1016/j.bbr.2008.06.024 18639588

[B208] Kitabgi P. Rostène W. Dussaillant M. Schotte A. Laduron P. M. Vincent J. P. (1987). Two populations of neurotensin binding sites in murine brain: discrimination by the antihistamine levocabastine reveals markedly different radioautographic distribution. Eur. J. Pharmacol. 140, 285–293. 10.1016/0014-2999(87)90285-8 2888670

[B209] Kiwaki K. Kotz C. M. Wang C. Lanningham-Foster L. Levine J. A. (2004). Orexin A (hypocretin 1) injected into hypothalamic paraventricular nucleus and spontaneous physical activity in rats. Am. J. Physiol. Endocrinol. Metab. 286, E551–559. 10.1152/ajpendo.00126.2003 14656716

[B210] Kleczkowska P. Lipkowski A. W. (2013). Neurotensin and neurotensin receptors: characteristic, structure-activity relationship and pain modulation–a review. Eur. J. Pharmacol. 716, 54–60. 10.1016/j.ejphar.2013.03.004 23500196

[B211] Konadhode R. R. Pelluru D. Blanco-Centurion C. Zayachkivsky A. Liu M. Uhde T.. (2013). Optogenetic stimulation of MCH neurons increases sleep. J. Neurosci. 33, 10257–10263. 10.1523/jneurosci.1225-13.2013 23785141 PMC3685832

[B212] Kong D. Vong L. Parton L. E. Ye C. Tong Q. Hu X.. (2010). Glucose stimulation of hypothalamic MCH neurons involves K(ATP) channels, is modulated by UCP2 and regulates peripheral glucose homeostasis. Cell Metab. 12, 545–552. 10.1016/j.cmet.2010.09.013 21035764 PMC2998191

[B213] Korotkova T. M. Sergeeva O. A. Eriksson K. S. Haas H. L. Brown R. E. (2003). Excitation of ventral tegmental area dopaminergic and nondopaminergic neurons by orexins/hypocretins. J. Neurosci. 23, 7–11. 12514194 10.1523/JNEUROSCI.23-01-00007.2003 PMC6742159

[B214] Kotz C. M. Wang C. Teske J. A. Thorpe A. J. Novak C. M. Kiwaki K.. (2006). Orexin A mediation of time spent moving in rats: neural mechanisms. Neuroscience 142, 29–36. 10.1016/j.neuroscience.2006.05.028 16809007

[B215] Kowalski T. J. Farley C. Cohen-Williams M. E. Varty G. Spar B. D. (2004). Melanin-concentrating hormone-1 receptor antagonism decreases feeding by reducing meal size. Eur. J. Pharmacol. 497, 41–47. 10.1016/j.ejphar.2004.06.027 15321733

[B216] Kowalski T. J. Spar B. D. Weig B. Farley C. Cook J. Ghibaudi L.. (2006). Effects of a selective melanin-concentrating hormone 1 receptor antagonist on food intake and energy homeostasis in diet-induced obese mice. Eur. J. Pharmacol. 535, 182–191. 10.1016/j.ejphar.2006.01.062 16540104

[B217] Kunii K. Yamanaka A. Nambu T. Matsuzaki I. Goto K. Sakurai T. (1999). Orexins/hypocretins regulate drinking behaviour. Brain Res. 842, 256–261. 10.1016/s0006-8993(99)01884-3 10526122

[B218] Lagos P. Torterolo P. Jantos H. Chase M. H. Monti J. M. (2009). Effects on sleep of melanin-concentrating hormone (MCH) microinjections into the dorsal raphe nucleus. Brain Res. 1265, 103–110. 10.1016/j.brainres.2009.02.010 19230831

[B219] Laque A. Zhang Y. Gettys S. Nguyen T. A. Bui K. Morrison C. D.. (2013). Leptin receptor neurons in the mouse hypothalamus are co-localized with the neuropeptide galanin and mediate anorexigenic leptin action. Am. J. Physiol. Endocrinol. Metab. 304, E999–E1011. 10.1152/ajpendo.00643.2012 23482448 PMC3651648

[B220] Lawrence C. B. Snape A. C. Baudoin F. M. Luckman S. M. (2002). Acute central ghrelin and GH secretagogues induce feeding and activate brain appetite centers. Endocrinology 143, 155–162. 10.1210/en.143.1.155 11751604

[B221] Lee M. G. Hassani O. K. Jones B. E. (2005). Discharge of identified orexin/hypocretin neurons across the sleep-waking cycle. J. Neurosci. 25, 6716–6720. 10.1523/jneurosci.1887-05.2005 16014733 PMC6725432

[B222] Lee M. R. Hinton D. J. Song J. Y. Lee K. W. Choo C. Johng H.. (2010). Neurotensin receptor type 1 regulates ethanol intoxication and consumption in mice. Pharmacol. Biochem. Behav. 95, 235–241. 10.1016/j.pbb.2010.01.012 20122953 PMC2830308

[B223] Lee M. R. Hinton D. J. Unal S. S. Richelson E. Choi D. S. (2011). Increased ethanol consumption and preference in mice lacking neurotensin receptor type 2. Alcohol. Clin. Exp. Res. 35, 99–107. 10.1111/j.1530-0277.2010.01326.x 21039631 PMC3058519

[B224] Lee S. J. Kirigiti M. Lindsley S. R. Loche A. Madden C. J. Morrison S. F.. (2013). Efferent projections of neuropeptide Y-expressing neurons of the dorsomedial hypothalamus in chronic hyperphagic models. J. Comp. Neurol. 521, 1891–1914. 10.1002/cne.23265 23172177 PMC3618613

[B225] Lee Y. C. Uttenthal L. O. Smith H. A. Bloom S. R. (1986). In vitro degradation of neurotensin in human plasma. Peptides 7, 383–387. 10.1016/0196-9781(86)90002-1 3774585

[B226] Legault M. Congar P. Michel F. J. Trudeau L. E. (2002). Presynaptic action of neurotensin on cultured ventral tegmental area dopaminergic neurones. Neuroscience 111, 177–187. 10.1016/s0306-4522(01)00614-5 11955721

[B227] Leinninger G. M. Jo Y. H. Leshan R. L. Louis G. W. Yang H. Barrera J. G.. (2009). Leptin acts via leptin receptor-expressing lateral hypothalamic neurons to modulate the mesolimbic dopamine system and suppress feeding. Cell Metab. 10, 89–98. 10.1016/j.cmet.2009.06.011 19656487 PMC2723060

[B228] Leinninger G. M. Opland D. M. Jo Y. H. Faouzi M. Christensen L. Cappellucci L. A.. (2011). Leptin action via neurotensin neurons controls orexin, the mesolimbic dopamine system and energy balance. Cell Metab. 14, 313–323. 10.1016/j.cmet.2011.06.016 21907138 PMC3183584

[B229] Lembo P. M. Grazzini E. Cao J. Hubatsch D. A. Pelletier M. Hoffert C.. (1999). The receptor for the orexigenic peptide melanin-concentrating hormone is a G-protein-coupled receptor. Nat. Cell Biol. 1, 267–271. 10559938 10.1038/12978

[B230] Levitt D. R. Teitelbaum P. (1975). Somnolence, akinesia and sensory activation of motivated behavior in the lateral hypothalamic syndrome. Proc. Natl. Acad. Sci. U S A 72, 2819–2823. 10.1073/pnas.72.7.2819 1101268 PMC432863

[B231] Li Y. Gao X. B. Sakurai T. van den Pol A. N. (2002). Hypocretin/Orexin excites hypocretin neurons via a local glutamate neuron-A potential mechanism for orchestrating the hypothalamic arousal system. Neuron 36, 1169–1181. 10.1016/s0896-6273(02)01132-7 12495630

[B232] Li Y. van den Pol A. N. (2006). Differential target-dependent actions of coexpressed inhibitory dynorphin and excitatory hypocretin/orexin neuropeptides. J. Neurosci. 26, 13037–13047. 10.1523/jneurosci.3380-06.2006 17167093 PMC6674960

[B233] Liu M. Thankachan S. Kaur S. Begum S. Blanco-Centurion C. Sakurai T.. (2008). Orexin (hypocretin) gene transfer diminishes narcoleptic sleep behavior in mice. Eur J. Neurosci. 28, 1382–1393. 10.1111/j.1460-9568.2008.06446.x 18973565 PMC2615183

[B234] Look A. R. G. Wing R. R. Bolin P. Brancati F. L. Bray G. A. Clark J. M.. (2013). Cardiovascular effects of intensive lifestyle intervention in type 2 diabetes. N. Engl. J. Med. 369, 145–154. 10.1056/NEJMoa1212914 23796131 PMC3791615

[B235] Louis G. W. Leinninger G. M. Rhodes C. J. Myers M. G. Jr. (2010). Direct innervation and modulation of orexin neurons by lateral hypothalamic LepRb neurons. J. Neurosci. 30, 11278–11287. 10.1523/jneurosci.1340-10.2010 20739548 PMC2930259

[B236] Lu B. Su Y. Das S. Wang H. Wang Y. Liu J.. (2009). Peptide neurotransmitters activate a cation channel complex of NALCN and UNC-80. Nature 457, 741–744. 10.1038/nature07579 19092807 PMC2810458

[B237] Ludwig D. S. Tritos N. A. Mastaitis J. W. Kulkarni R. Kokkotou E. Elmquist J.. (2001). Melanin-concentrating hormone overexpression in transgenic mice leads to obesity and insulin resistance. J. Clin. Invest. 107, 379–386. 10.1172/jci10660 11160162 PMC199192

[B238] Lutter M. Krishnan V. Russo S. J. Jung S. Mcclung C. A. Nestler E. J. (2008). Orexin signaling mediates the antidepressant-like effect of calorie restriction. J. Neurosci. 28, 3071–3075. 10.1523/jneurosci.5584-07.2008 18354010 PMC2713756

[B239] Mahler S. V. Smith R. J. Aston-Jones G. (2013). Interactions between VTA orexin and glutamate in cue-induced reinstatement of cocaine seeking in rats. Psychopharmacology (Berl) 226, 687–698. 10.1007/s00213-012-2681-5 22411428 PMC3649073

[B240] Marsh D. J. Weingarth D. T. Novi D. E. Chen H. Y. Trumbauer M. E. Chen A. S.. (2002). Melanin-concentrating hormone 1 receptor-deficient mice are lean, hyperactive and hyperphagic and have altered metabolism. Proc. Natl. Acad. Sci. U S A 99, 3240–3245. 10.1073/pnas.052706899 11867747 PMC122503

[B241] Marshall J. F. Teitelbaum P. (1973). A comparison of the eating in response to hypothermic and glucoprivic challenges after nigral 6-hydroxydopamine and lateral hypothalamic electrolytic lesions in rats. Brain Res. 55, 229–233. 10.1016/0006-8993(73)90507-6 4514582

[B242] Martin G. E. Bacino C. B. Papp N. L. (1980). Hypothermia elicited by the intracerebral microinjection of neurotensin. Peptides 1, 333–339. 10.1016/0196-9781(80)90011-x 7301636

[B243] Martin G. Fabre V. Siggins G. R. De Lecea L. (2002). Interaction of the hypocretins with neurotransmitters in the nucleus accumbens. Regul. Pept. 104, 111–117. 10.1016/s0167-0115(01)00354-8 11830285

[B244] Matsuki T. Nomiyama M. Takahira H. Hirashima N. Kunita S. Takahashi S.. (2009). Selective loss of GABA(B) receptors in orexin-producing neurons results in disrupted sleep/wakefulness architecture. Proc. Natl. Acad. Sci. U S A 106, 4459–4464. 10.1073/pnas.0811126106 19246384 PMC2657380

[B245] Matsuo E. Mochizuki A. Nakayama K. Nakamura S. Yamamoto T. Shioda S.. (2010). Decreased intake of sucrose solutions in orexin knockout mice. J. Mol. Neurosci. 43, 217–224. 10.1007/s12031-010-9475-1 21086064

[B246] Mazella J. Botto J. M. Guillemare E. Coppola T. Sarret P. Vincent J. P. (1996). Structure, functional expression and cerebral localization of the levocabastine-sensitive neurotensin/neuromedin N receptor from mouse brain. J. Neurosci. 16, 5613–5620. 8795617 10.1523/JNEUROSCI.16-18-05613.1996 PMC6578974

[B247] Mazella J. Kitabgi P. Vincent J. P. (1985). Molecular properties of neurotensin receptors in rat brain. Identification of subunits by covalent labeling. J. Biol. Chem. 260, 508–514. 10.1016/0196-9781(85)90440-1 2981215

[B248] Mazella J. Zsürger N. Navarro V. Chabry J. Kaghad M. Caput D.. (1998). The 100-kDa neurotensin receptor is gp95/sortilin, a non-G-protein-coupled receptor. J. Biol. Chem. 273, 26273–26276. 10.1074/jbc.273.41.26273 9756851

[B249] McDermott J. R. Virmani M. A. Turner J. D. Kidd A. M. (1986). Peptidases involved in the catabolism of neurotensin: inhibitor studies using superfused rat hypothalamic slices. Peptides 7, 225–230. 10.1016/0196-9781(86)90217-2 3526299

[B250] McGregor R. Wu M. F. Barber G. Ramanathan L. Siegel J. M. (2011). Highly specific role of hypocretin (orexin) neurons: differential activation as a function of diurnal phase, operant reinforcement versus operant avoidance and light level. J. Neurosci. 31, 15455–15467. 10.1523/JNEUROSCI.4017-11.2011 22031892 PMC3230273

[B251] Meerabux J. Iwayama Y. Sakurai T. Ohba H. Toyota T. Yamada K.. (2005). Association of an orexin 1 receptor 408Val variant with polydipsia-hyponatremia in schizophrenic subjects. Biol. Psychiatry 58, 401–407. 10.1016/j.biopsych.2005.04.015 15978554

[B252] Meguid M. M. Glade M. J. Middleton F. A. (2008). Weight regain after Roux-en-Y: a significant 20% complication related to PYY. Nutrition 24, 832–842. 10.1016/j.nut.2008.06.027 18725080

[B253] Mieda M. Hasegawa E. Kisanuki Y. Y. Sinton C. M. Yanagisawa M. Sakurai T. (2011). Differential roles of orexin receptor-1 and -2 in the regulation of non-REM and REM sleep. J. Neurosci. 31, 6518–6526. 10.1523/JNEUROSCI.6506-10.2011 21525292 PMC3732784

[B254] Mieda M. Williams S. C. Sinton C. M. Richardson J. A. Sakurai T. Yanagisawa M. (2004). Orexin neurons function in an efferent pathway of a food-entrainable circadian oscillator in eliciting food-anticipatory activity and wakefulness. J. Neurosci. 24, 10493–10501. 10.1523/jneurosci.3171-04.2004 15548664 PMC6730290

[B255] Milella M. S. Passarelli F. De Carolis L. Schepisi C. Nativio P. Scaccianoce S.. (2010). Opposite roles of dopamine and orexin in quinpirole-induced excessive drinking: a rat model of psychotic polydipsia. Psychopharmacology (Berl) 211, 355–366. 10.1007/s00213-010-1909-5 20552172

[B256] Mileykovskiy B. Y. Kiyashchenko L. I. Siegel J. M. (2005). Behavioral correlates of activity in identified hypocretin/orexin neurons. Neuron 46, 787–798. 10.1016/j.neuron.2005.04.035 15924864 PMC8281334

[B257] Mochizuki T. Arrigoni E. Marcus J. N. Clark E. L. Yamamoto M. Honer M.. (2011). Orexin receptor 2 expression in the posterior hypothalamus rescues sleepiness in narcoleptic mice. Proc. Natl. Acad. Sci. U S A 108, 4471–4476. 10.1073/pnas.1012456108 21368172 PMC3060231

[B258] Mochizuki T. Crocker A. Mccormack S. Yanagisawa M. Sakurai T. Scammell T. E. (2004). Behavioral state instability in orexin knock-out mice. J. Neurosci. 24, 6291–6300. 10.1523/jneurosci.0586-04.2004 15254084 PMC6729542

[B259] Moga M. M. Saper C. B. Gray T. S. (1990). Neuropeptide organization of the hypothalamic projection to the parabrachial nucleus in the rat. J. Comp. Neurol. 295, 662–682. 10.1002/cne.902950409 1972710

[B260] Mogenson G. J. Morgan C. W. (1967). Effects of induced drinking on self-stimulation of the lateral hypothalamus. Exp. Brain Res. 3, 111–116. 6031542 10.1007/BF00233256

[B261] Mogenson G. J. Stevenson J. A. (1967). Drinking induced by electrical stimulation of the lateral hypothalamus. Exp. Neurol. 17, 119–127. 10.1016/0014-4886(67)90139-2 6018354

[B262] Montague C. T. Farooqi I. S. Whitehead J. P. Soos M. A. Rau H. Wareham N. J.. (1997). Congenital leptin deficiency is associated with severe early-onset obesity in humans. Nature 387, 903–908. 9202122 10.1038/43185

[B263] Morgane P. J. (1961). Distinct “feeding” and “hunger motivating” systems in the lateral hypothalamus of the rat. Science 133, 887–888. 10.1126/science.133.3456.887 13772611

[B264] Morrison S. D. Barrnett R. J. Mayer J. (1958). Localization of lesions in the lateral hypothalamus of rats with induced adipsia and aphagia. Am. J. Physiol. 193, 230–234. 13521013 10.1152/ajplegacy.1958.193.1.230

[B265] Mouri T. Takahashi K. Kawauchi H. Sone M. Totsune K. Murakami O.. (1993). Melanin-concentrating hormone in the human brain. Peptides 14, 643–646. 10.1016/0196-9781(93)90158-d 8332560

[B266] Moyse E. Rostene W. Vial M. Leonard K. Mazella J. Kitabgi P. (1987). Distribution of neurotensin binding sites in rat brain: a light microscopic radioautographic study using monoiodo [125I]Tyr3-neurotensin. Neuroscience 22, 525–536 10.1016/0306-4522(87)90350-2 3313097

[B267] Muschamp J. W. Hollander J. A. Thompson J. L. Voren G. Hassinger L. C. Onvani S.. (2014). Hypocretin (orexin) facilitates reward by attenuating the antireward effects of its cotransmitter dynorphin in ventral tegmental area. Proc. Natl. Acad. Sci. U S A 111, E1648–E1655. 10.1073/pnas.1315542111 24706819 PMC4000785

[B268] Myers M. G. Jr. Münzberg H. Leinninger G. M. Leshan R. L. (2009). The geometry of leptin action in the brain: more complicated than a simple ARC. Cell Metab. 9, 117–123. 10.1016/j.cmet.2008.12.001 19187770 PMC2648854

[B269] Najimi M. Sarrieau A. Kopp N. Chigr F. (2014). An autoradiographic study of neurotensin receptors in the human hypothalamus. Acta Histochem. 116, 382–389. 10.1016/j.acthis.2013.09.008 24144485

[B270] Nakazato M. Murakami N. Date Y. Kojima M. Matsuo H. Kangawa K.. (2001). A role for ghrelin in the central regulation of feeding. Nature 409, 194–198. 10.1038/35051587 11196643

[B271] Nalivaiko E. Michaud J. C. Soubrié P. Le Fur G. (1998). Electrophysiological evidence for putative subtypes of neurotensin receptors in guinea-pig mesencephalic dopaminergic neurons. Neuroscience 86, 799–811. 10.1016/s0306-4522(98)00084-0 9692718

[B272] Narita M. Nagumo Y. Hashimoto S. Narita M. Khotib J. Miyatake M.. (2006). Direct involvement of orexinergic systems in the activation of the mesolimbic dopamine pathway and related behaviors induced by morphine. J. Neurosci. 26, 398–405. 10.1523/jneurosci.2761-05.2006 16407535 PMC6674410

[B273] Nemeroff C. B. Bissette G. Prange A. J. Jr. Loosen P. T. Barlow T. S. Lipton M. A. (1977). Neurotensin: central nervous system effects of a hypothalamic peptide. Brain Res. 128, 485–496. 10.1016/0006-8993(77)90173-1 406965

[B274] Nemeroff C. B. Hernandez D. E. Luttinger D. Kalivas P. W. Prange A. J. Jr. (1982). Interactions of neurotensin with brain dopamine systems. Ann. N Y Acad. Sci. 400, 330–344. 10.1111/j.1749-6632.1982.tb31579.x 6132577

[B275] Nishino S. Ripley B. Overeem S. Lammers G. J. Mignot E. (2000). Hypocretin (orexin) deficiency in human narcolepsy. Lancet 355, 39–40. 10.1016/s0140-6736(99)05582-8 10615891

[B276] Nishino S. Ripley B. Overeem S. Nevsimalova S. Lammers G. J. Vankova J.. (2001). Low cerebrospinal fluid hypocretin (Orexin) and altered energy homeostasis in human narcolepsy. Ann. Neurol. 50, 381–388. 10.1002/ana.1130 11558795

[B277] Nogueiras R. Tschöp M. H. Zigman J. M. (2008). Central nervous system regulation of energy metabolism: ghrelin versus leptin. Ann. N Y Acad. Sci. 1126, 14–19. 10.1196/annals.1433.054 18448790 PMC2814160

[B278] Nouel D. Faure M. P. St Pierre J. A. Alonso R. Quirion R. Beaudet A. (1997). Differential binding profile and internalization process of neurotensin via neuronal and glial receptors. J. Neurosci. 17, 1795–1803. 9030638 10.1523/JNEUROSCI.17-05-01795.1997 PMC6573365

[B279] Nouel D. Sarret P. Vincent J. P. Mazella J. Beaudet A. (1999). Pharmacological, molecular and functional characterization of glial neurotensin receptors. Neuroscience 94, 1189–1197. 10.1196/annals.1433.054 10625058

[B280] Obici S. Zhang B. B. Karkanias G. Rossetti L. (2002). Hypothalamic insulin signaling is required for inhibition of glucose production. Nat. Med. 8, 1376–1382. 10.1038/nm1202-798 12426561

[B281] Oldfield B. J. Allen A. M. Davern P. Giles M. E. Owens N. C. (2007). Lateral hypothalamic ’command neurons’ with axonal projections to regions involved in both feeding and thermogenesis. Eur J. Neurosci. 25, 2404–2412. 10.1111/j.1460-9568.2007.05429.x 17445237

[B282] Oldfield B. J. Giles M. E. Watson A. Anderson C. Colvill L. M. Mckinley M. J. (2002). The neurochemical characterisation of hypothalamic pathways projecting polysynaptically to brown adipose tissue in the rat. Neuroscience 110, 515–526. 10.1016/s0306-4522(01)00555-3 11906790

[B283] Olszewski P. K. Li D. Grace M. K. Billington C. J. Kotz C. M. Levine A. S. (2003). Neural basis of orexigenic effects of ghrelin acting within lateral hypothalamus. Peptides 24, 597–602. 10.1016/s0196-9781(03)00105-0 12860204

[B284] Ono K. Kai A. Honda E. Inenaga K. (2008). Hypocretin-1/orexin-A activates subfornical organ neurons of rats. Neuroreport 19, 69–73. 10.1097/wnr.0b013e3282f32d64 18281895

[B285] Opland D. Sutton S. A. Woodworth H. Brown J. Bugescu R. Garcia A.. (2013). Loss of neurotensin receptor-1 disrupts the control of the mesolimbic dopamine system by leptin and promotes hedonic feeding and obesity. Mol. Metab. 2, 423–434. 10.1016/j.molmet.2013.07.008 24327958 PMC3857883

[B286] Osbahr A. J. 3rd Nemeroff C. B. Luttinger D. Mason G. A. Prange A. J. Jr. (1981). Neurotensin-induced antinociception in mice: antagonism by thyrotropin-releasing hormone. J. Pharmacol. Exp. Ther. 217, 645–651. 6112261

[B287] Oshima N. Kasukawa H. Fujii R. Wilkes B. C. Hruby V. J. Hadley M. E. (1986). Action of melanin-concentrating hormone (MCH) on teleost chromatophores. Gen. Comp. Endocrinol. 64, 381–388. 10.1016/0016-6480(86)90072-9 3026881

[B288] Pankevich D. E. Teegarden S. L. Hedin A. D. Jensen C. L. Bale T. L. (2010). Caloric restriction experience reprograms stress and orexigenic pathways and promotes binge eating. J. Neurosci. 30, 16399–16407. 10.1523/jneurosci.1955-10.2010 21123586 PMC3034235

[B289] Park S. M. Gaykema R. P. Goehler L. E. (2008). How does immune challenge inhibit ingestion of palatable food? Evidence that systemic lipopolysaccharide treatment modulates key nodal points of feeding neurocircuitry. Brain Behav. Immun. 22, 1160–1172. 10.1016/j.bbi.2008.05.001 18562160 PMC2784149

[B290] Parks G. S. Olivas N. D. Ikrar T. Sanathara N. M. Wang L. Wang Z.. (2014). Histamine inhibits the melanin-concentrating hormone system: implications for sleep and arousal. J. Physiol. 592, 2183–2196. 10.1113/jphysiol.2013.268771 24639485 PMC4227902

[B441] Paxinos G. Franklin B. (2001). The Mouse Brain in Stereotaxic Coordinates. 2nd Edn. Academic Press.

[B291] Pelleymounter M. A. Cullen M. J. Baker M. B. Hecht R. Winters D. Boone T.. (1995). Effects of the obese gene product on body weight regulation in ob/ob mice. Science 269, 540–543. 10.1126/science.7624776 7624776

[B292] Pereira-Da-Silva M. Torsoni M. A. Nourani H. V. Augusto V. D. Souza C. T. Gasparetti A. L.. (2003). Hypothalamic melanin-concentrating hormone is induced by cold exposure and participates in the control of energy expenditure in rats. Endocrinology 144, 4831–4840. 10.1210/en.2003-0243 12960043

[B293] Perello M. Sakata I. Birnbaum S. Chuang J. C. Osborne-Lawrence S. Rovinsky S. A.. (2010). Ghrelin increases the rewarding value of high-fat diet in an orexin-dependent manner. Biol. Psychiatry 67, 880–886. 10.1016/j.biopsych.2009.10.030 20034618 PMC2854245

[B294] Petrovich G. D. Hobin M. P. Reppucci C. J. (2012). Selective Fos induction in hypothalamic orexin/hypocretin, but not melanin-concentrating hormone neurons, by a learned food-cue that stimulates feeding in sated rats. Neuroscience 224, 70–80. 10.1016/j.neuroscience.2012.08.036 22922124 PMC3689536

[B295] Pettibone D. J. Hess J. F. Hey P. J. Jacobson M. A. Leviten M. Lis E. V.. (2002). The effects of deleting the mouse neurotensin receptor NTR1 on central and peripheral responses to neurotensin. J. Pharmacol. Exp. Ther. 300, 305–313. 10.1124/jpet.300.1.305 11752130

[B296] Peyron C. Faraco J. Rogers W. Ripley B. Overeem S. Charnay Y.. (2000). A mutation in a case of early onset narcolepsy and a generalized absence of hypocretin peptides in human narcoleptic brains. Nat. Med. 6, 991–997. 10.1038/79690 10973318

[B297] Peyron C. Tighe D. K. van den Pol A. N. De Lecea L. Heller H. C. Sutcliffe J. G.. (1998). Neurons containing hypocretin (orexin) project to multiple neuronal systems. J. Neurosci. 18, 9996–10015. 9822755 10.1523/JNEUROSCI.18-23-09996.1998 PMC6793310

[B298] Pissios P. Frank L. Kennedy A. R. Porter D. R. Marino F. E. Liu F. F.. (2008). Dysregulation of the mesolimbic dopamine system and reward in MCH−/− mice. Biol. Psychiatry 64, 184–191. 10.1016/j.biopsych.2007.12.011 18281019

[B299] Pissios P. Trombly D. J. Tzameli I. Maratos-Flier E. (2003). Melanin-concentrating hormone receptor 1 activates extracellular signal-regulated kinase and synergizes with G(s)-coupled pathways. Endocrinology 144, 3514–3523. 10.1210/en.2002-0004 12865333

[B300] Poli F. Plazzi G. Di Dalmazi G. Ribichini D. Vicennati V. Pizza F.. (2009). Body mass index-independent metabolic alterations in narcolepsy with cataplexy. Sleep 32, 1491–1497. 19928388 10.1093/sleep/32.11.1491 PMC2768955

[B301] Popp E. Schneider A. Vogel P. Teschendorf P. Bottiger B. W. (2007). Time course of the hypothermic response to continuously administered neurotensin. Neuropeptides 41, 349–354. 10.1016/j.npep.2007.06.002 17655926

[B302] Pozza M. F. Kung E. Bischoff S. Olpe H. R. (1988). The neurotensin analog xenopsin excites nigral dopamine neurons. Eur. J. Pharmacol. 145, 341–343. 10.1016/0014-2999(88)90439-6 3350051

[B303] Qu D. Ludwig D. S. Gammeltoft S. Piper M. Pelleymounter M. A. Cullen M. J.. (1996). A role for melanin-concentrating hormone in the central regulation of feeding behaviour. Nature 380, 243–247. 10.1038/380243a0 8637571

[B304] Quirk W. S. Wright J. W. Harding J. W. (1988). Tachyphylaxis of dipsogenic activity to intracerebroventricular administration of angiotensins. Brain Res. 452, 73–78. 10.1016/0006-8993(88)90010-8 3401750

[B305] Rai S. Kumar S. Alam M. A. Szymusiak R. Mcginty D. Alam M. N. (2010). A1 receptor mediated adenosinergic regulation of perifornical-lateral hypothalamic area neurons in freely behaving rats. Neuroscience 167, 40–48. 10.1016/j.neuroscience.2010.01.044 20109537 PMC2842084

[B306] Ramanjaneya M. Conner A. C. Chen J. Kumar P. Brown J. E. Johren O.. (2009). Orexin-stimulated MAP kinase cascades are activated through multiple G-protein signalling pathways in human H295R adrenocortical cells: diverse roles for orexins A and B. J. Endocrinol. 202, 249–261. 10.1677/joe-08-0536 19460850

[B307] Ranson S. W. (1937). Some functions of the hypothalamus: Harvey Lecture, December 17, 1936. Bull. N Y Acad. Med. 13, 241–271. 19312019 PMC1966114

[B308] Rao Y. Liu Z. W. Borok E. Rabenstein R. L. Shanabrough M. Lu M.. (2007). Prolonged wakefulness induces experience-dependent synaptic plasticity in mouse hypocretin/orexin neurons. J. Clin. Invest. 117, 4022–4033. 10.1172/jci32829 18060037 PMC2104495

[B309] Rao Y. Lu M. Ge F. Marsh D. J. Qian S. Wang A. H.. (2008). Regulation of synaptic efficacy in hypocretin/orexin-containing neurons by melanin concentrating hormone in the lateral hypothalamus. J. Neurosci. 28, 9101–9110. 10.1523/jneurosci.1766-08.2008 18784290 PMC2562258

[B310] Remaury A. Vita N. Gendreau S. Jung M. Arnone M. Poncelet M.. (2002). Targeted inactivation of the neurotensin type 1 receptor reveals its role in body temperature control and feeding behavior but not in analgesia. Brain Res. 953, 63–72. 10.1016/s0006-8993(02)03271-7 12384239

[B311] Reti I. M. Reddy R. Worley P. F. Baraban J. M. (2002). Selective expression of Narp, a secreted neuronal pentraxin, in orexin neurons. J. Neurochem. 82, 1561–1565. 10.1046/j.1471-4159.2002.01141.x 12354306

[B312] Richardson K. A. Aston-Jones G. (2012). Lateral hypothalamic orexin/hypocretin neurons that project to ventral tegmental area are differentially activated with morphine preference. J. Neurosci. 32, 3809–3817. 10.1523/jneurosci.3917-11.2012 22423101 PMC3321304

[B313] Richy S. Burlet A. Max J. Burlet C. Beck B. (2000). Effect of chronic intraperitoneal injections of leptin on hypothalamic neurotensin content and food intake. Brain Res. 862, 276–279. 10.1016/s0006-8993(00)02125-9 10799699

[B314] Roberts G. W. Woodhams P. L. Polak J. M. Crow T. J. (1984). Distribution of neuropeptides in the limbic system of the rat: the hippocampus. Neuroscience 11, 35–77. 10.1016/0306-4522(84)90214-8 6200800

[B315] Rodgers R. J. Halford J. C. Nunes De Souza R. L. Canto De Souza A. L. Piper D. C. Arch J. R.. (2001). SB-334867, a selective orexin-1 receptor antagonist, enhances behavioural satiety and blocks the hyperphagic effect of orexin-A in rats. Eur J. Neurosci. 13, 1444–1452. 10.1046/j.0953-816x.2001.01518.x 11298806

[B316] Rodriguez M. Beauverger P. Naime I. Rique H. Ouvry C. Souchaud S.. (2001). Cloning and molecular characterization of the novel human melanin-concentrating hormone receptor MCH2. Mol. Pharmacol. 60, 632–639. 11562423

[B317] Rolls A. Colas D. Adamantidis A. Carter M. Lanre-Amos T. Heller H. C.. (2011). Optogenetic disruption of sleep continuity impairs memory consolidation. Proc. Natl. Acad. Sci. U S A 108, 13305–13310. 10.1073/pnas.1015633108 21788501 PMC3156195

[B318] Rosin D. L. Weston M. C. Sevigny C. P. Stornetta R. L. Guyenet P. G. (2003). Hypothalamic orexin (hypocretin) neurons express vesicular glutamate transporters VGLUT1 or VGLUT2. J. Comp. Neurol. 465, 593–603. 10.1002/cne.10860 12975818

[B319] Rossi M. Choi S. J. O’shea D. Miyoshi T. Ghatei M. A. Bloom S. R. (1997). Melanin-concentrating hormone acutely stimulates feeding, but chronic administration has no effect on body weight. Endocrinology 138, 351–355. 10.1210/en.138.1.351 8977423

[B320] Rovere C. Barbero P. Kitabgi P. (1996a). Evidence that PC2 is the endogenous pro-neurotensin convertase in rMTC 6–23 cells and that PC1- and PC2-transfected PC12 cells differentially process pro-neurotensin. J. Biol. Chem. 271, 11368–11375. 10.1074/jbc.271.19.11368 8626691

[B321] Rovere C. Viale A. Nahon J. Kitabgi P. (1996b). Impaired processing of brain proneurotensin and promelanin-concentrating hormone in obese fat/fat mice. Endocrinology 137, 2954–2958. 10.1210/en.137.7.2954 8770919

[B322] Sahu A. (1998). Evidence suggesting that galanin (GAL), melanin-concentrating hormone (MCH), neurotensin (NT), proopiomelanocortin (POMC) and neuropeptide Y (NPY) are targets of leptin signaling in the hypothalamus. Endocrinology 139, 795–798. 10.1210/endo.139.2.5909 9449656

[B323] Sahu A. Carraway R. E. Wang Y. P. (2001). Evidence that neurotensin mediates the central effect of leptin on food intake in rat. Brain Res. 888, 343–347. 10.1016/s0006-8993(00)03107-3 11150496

[B324] Saito Y. Nothacker H. P. Wang Z. Lin S. H. Leslie F. Civelli O. (1999). Molecular characterization of the melanin-concentrating-hormone receptor. Nature 400, 265–269. 10421368 10.1038/22321

[B325] Sakamaki R. Uemoto M. Inui A. Asakawa A. Ueno N. Ishibashi C.. (2005). Melanin-concentrating hormone enhances sucrose intake. Int. J. Mol. Med. 15, 1033–1039. 10.3892/ijmm.15.6.1033 15870910

[B326] Sakurai T. Amemiya A. Ishii M. Matsuzaki I. Chemelli R. M. Tanaka H.. (1998). Orexins and orexin receptors: a family of hypothalamic neuropeptides and G protein-coupled receptors that regulate feeding behavior. Cell 92, 573–585. 10.1016/s0092-8674(00)80949-6 9491897

[B327] Sandoval S. L. Kulkosky P. J. (1992). Effects of peripheral neurotensin on behavior of the rat. Pharmacol. Biochem. Behav. 41, 385–390. 10.1016/0091-3057(92)90115-v 1574529

[B328] Sapin E. Bérod A. Léger L. Herman P. A. Luppi P. H. Peyron C. (2010). A very large number of GABAergic neurons are activated in the tuberal hypothalamus during paradoxical (REM) sleep hypersomnia. PLoS One 5:e11766. 10.1371/journal.pone.0011766 20668680 PMC2909908

[B329] Schone C. Cao Z. F. Apergis-Schoute J. Adamantidis A. Sakurai T. Burdakov D. (2012). Optogenetic probing of fast glutamatergic transmission from hypocretin/orexin to histamine neurons in situ. J. Neurosci. 32, 12437–12443. 10.1523/jneurosci.0706-12.2012 22956835 PMC6621251

[B330] Schöne C. Venner A. Knowles D. Karnani M. M. Burdakov D. (2011). Dichotomous cellular properties of mouse orexin/hypocretin neurons. J. Physiol. 589, 2767–2779. 10.1113/jphysiol.2011.208637 21486780 PMC3112554

[B331] Schuld A. Hebebrand J. Geller F. Pollmacher T. (2000). Increased body-mass index in patients with narcolepsy. Lancet 355, 1274–1275. 10.1016/s0140-6736(05)74704-8 10770327

[B332] Schwartz M. W. Bergman R. N. Kahn S. E. Taborsky G. J. Jr. Fisher L. D. Sipols A. J.. (1991). Evidence for entry of plasma insulin into cerebrospinal fluid through an intermediate compartment in dogs. Quantitative aspects and implications for transport. J. Clin. Invest. 88, 1272–1281. 10.1172/jci115431 1918377 PMC295596

[B333] Scott M. M. Marcus J. N. Pettersen A. Birnbaum S. G. Mochizuki T. Scammell T. E.. (2011). Hcrtr1 and 2 signaling differentially regulates depression-like behaviors. Behav. Brain Res. 222, 289–294. 10.1016/j.bbr.2011.02.044 21377495 PMC3474296

[B334] Sears R. M. Liu R. J. Narayanan N. S. Sharf R. Yeckel M. F. Laubach M.. (2010). Regulation of nucleus accumbens activity by the hypothalamic neuropeptide melanin-concentrating hormone. J. Neurosci. 30, 8263–8273. 10.1523/jneurosci.5858-09.2010 20554878 PMC2907886

[B335] Segal-Lieberman G. Bradley R. L. Kokkotou E. Carlson M. Trombly D. J. Wang X.. (2003). Melanin-concentrating hormone is a critical mediator of the leptin-deficient phenotype. Proc. Natl. Acad. Sci. U S A 100, 10085–10090. 10.1073/pnas.1633636100 12897241 PMC187774

[B336] Sellayah D. Bharaj P. Sikder D. (2011). Orexin is required for brown adipose tissue development, differentiation and function. Cell Metab. 14, 478–490. 10.1016/j.cmet.2011.08.010 21982708

[B337] Sergeeva O. A. Andreeva N. Garret M. Scherer A. Haas H. L. (2005). Pharmacological properties of GABAA receptors in rat hypothalamic neurons expressing the epsilon-subunit. J. Neurosci. 25, 88–95. 10.1523/jneurosci.3209-04.2005 15634770 PMC6725214

[B338] Seta K. A. Jansen H. T. Kreitel K. D. Lehman M. Behbehani M. M. (2001). Cold water swim stress increases the expression of neurotensin mRNA in the lateral hypothalamus and medial preoptic regions of the rat brain. Brain Res. Mol. Brain Res. 86, 145–152. 10.1016/s0169-328x(00)00279-5 11165381

[B339] Seutin V. Massotte L. Dresse A. (1989). Electrophysiological effects of neurotensin on dopaminergic neurones of the ventral tegmental area of the rat in vitro. Neuropharmacology 28, 949–954. 10.1016/0028-3908(89)90194-9 2572997

[B340] Sharf R. Sarhan M. Brayton C. E. Guarnieri D. J. Taylor J. R. Dileone R. J. (2010). Orexin signaling via the orexin 1 receptor mediates operant responding for food reinforcement. Biol. Psychiatry 67, 753–760. 10.1016/j.biopsych.2009.12.035 20189166 PMC2849869

[B341] Sheng Z. Santiago A. M. Thomas M. P. Routh V. H. (2014). Metabolic regulation of lateral hypothalamic glucose-inhibited orexin neurons may influence midbrain reward neurocircuitry. Mol. Cell. Neurosci. 62, 30–41. 10.1016/j.mcn.2014.08.001 25107627 PMC6524643

[B342] Sheppard M. C. Bailey C. J. Flatt P. R. Swanston-Flatt S. K. Shennan K. I. (1985). Immunoreactive neurotensin in spontaneous syndromes of obesity and diabetes in mice. Acta Endocrinol. (Copenh) 108, 532–536. 10.1530/acta.0.1080532 3887830

[B343] Sherwood A. Wosiski-Kuhn M. Nguyen T. Holland P. C. Lakaye B. Adamantidis A.. (2012). The role of melanin-concentrating hormone in conditioned reward learning. Eur J. Neurosci. 36, 3126–3133. 10.1111/j.1460-9568.2012.08207.x 22775118 PMC5575786

[B344] Shi W. X. Bunney B. S. (1991). Neurotensin modulates autoreceptor mediated dopamine effects on midbrain dopamine cell activity. Brain Res. 543, 315–321. 10.1016/0006-8993(91)90043-u 1676331

[B345] Shimada M. Tritos N. A. Lowell B. B. Flier J. S. Maratos-Flier E. (1998). Mice lacking melanin-concentrating hormone are hypophagic and lean. Nature 396, 670–674. 9872314 10.1038/25341

[B346] Shipley M. T. Mclean J. H. Behbehani M. M. (1987). Heterogeneous distribution of neurotensin-like immunoreactive neurons and fibers in the midbrain periaqueductal gray of the rat. J. Neurosci. 7, 2025–2034. 3302124 10.1523/JNEUROSCI.07-07-02025.1987 PMC6568941

[B347] Shirasaka T. Nakazato M. Matsukura S. Takasaki M. Kannan H. (1999). Sympathetic and cardiovascular actions of orexins in conscious rats. Am. J. Physiol. 277, R1780–R1785. 10600926 10.1152/ajpregu.1999.277.6.R1780

[B348] Shiuchi T. Haque M. S. Okamoto S. Inoue T. Kageyama H. Lee S.. (2009). Hypothalamic orexin stimulates feeding-associated glucose utilization in skeletal muscle via sympathetic nervous system. Cell Metab. 10, 466–480. 10.1016/j.cmet.2009.09.013 19945404

[B349] Singh J. Desiraju T. Raju T. R. (1997). Effects of microinjections of cholecystokinin and neurotensin into lateral hypothalamus and ventral mesencephalon on intracranial self-stimulation. Pharmacol. Biochem. Behav. 58, 893–898. 10.1016/s0091-3057(97)00040-3 9408192

[B350] Skoog K. M. Cain S. T. Nemeroff C. B. (1986). Centrally administered neurotensin suppresses locomotor hyperactivity induced by d-amphetamine but not by scopolamine or caffeine. Neuropharmacology 25, 777–782. 10.1016/0028-3908(86)90095-x 3748325

[B351] Smink F. R. van Hoeken D. Hoek H. W. (2012). Epidemiology of eating disorders: incidence, prevalence and mortality rates. Curr. Psychiatry Rep. 14, 406–414. 10.1007/s11920-012-0282-y 22644309 PMC3409365

[B354] Smith R. J. Aston-Jones G. (2012). Orexin / hypocretin 1 receptor antagonist reduces heroin self-administration and cue-induced heroin seeking. Eur J. Neurosci. 35, 798–804. 10.1111/j.1460-9568.2012.08013.x 22356621 PMC3295925

[B352] Smith D. G. Davis R. J. Rorick-Kehn L. Morin M. Witkin J. M. Mckinzie D. L.. (2006). Melanin-concentrating hormone-1 receptor modulates neuroendocrine, behavioral and corticolimbic neurochemical stress responses in mice. Neuropsychopharmacology 31, 1135–1145. 10.1038/sj.npp.1300913 16205780

[B353] Smith D. G. Tzavara E. T. Shaw J. Luecke S. Wade M. Davis R.. (2005). Mesolimbic dopamine super-sensitivity in melanin-concentrating hormone-1 receptor-deficient mice. J. Neurosci. 25, 914–922. 10.1523/jneurosci.4079-04.2005 15673672 PMC6725636

[B355] Sotty F. Soulière F. Brun P. Chouvet G. Steinberg R. Soubrié P.. (1998). Differential effects of neurotensin on dopamine release in the caudal and rostral nucleus accumbens: a combined in vivo electrochemical and electrophysiological study. Neuroscience 85, 1173–1182. 10.1016/s0306-4522(97)00691-x 9681955

[B356] Srinivasan S. Simms J. A. Nielsen C. K. Lieske S. P. Bito-Onon J. J. Yi H.. (2012). The dual orexin/hypocretin receptor antagonist, almorexant, in the ventral tegmental area attenuates ethanol self-administration. PLoS One 7:e44726. 10.1371/journal.pone.0044726 23028593 PMC3448615

[B357] Stanley B. G. Hoebel B. G. Leibowitz S. F. (1983). Neurotensin: effects of hypothalamic and intravenous injections on eating and drinking in rats. Peptides 4, 493–500. 10.1016/0196-9781(83)90054-2 6685868

[B358] Stanley S. Pinto S. Segal J. Pérez C. A. Viale A. Defalco J.. (2010). Identification of neuronal subpopulations that project from hypothalamus to both liver and adipose tissue polysynaptically. Proc. Natl. Acad. Sci. U S A 107, 7024–7029. 10.1073/pnas.100279010 20351287 PMC2872469

[B359] Steinberg R. Brun P. Fournier M. Souilhac J. Rodier D. Mons G.. (1994). SR 48692, a non-peptide neurotensin receptor antagonist differentially affects neurotensin-induced behaviour and changes in dopaminergic transmission. Neuroscience 59, 921–929. 10.1016/0306-4522(94)90295-x 8058127

[B360] Stellar E. (1954). The physiology of motivation. Psychol. Rev. 61, 5–22. 10.1037/h0060347 13134413

[B361] Sternson S. M. (2013). Hypothalamic survival circuits: blueprints for purposive behaviors. Neuron 77, 810–824. 10.1016/j.neuron.2013.02.018 23473313 PMC4306350

[B362] Strawn J. R. Pyne-Geithman G. J. Ekhator N. N. Horn P. S. Uhde T. W. Shutter L. A.. (2010). Low cerebrospinal fluid and plasma orexin-A (hypocretin-1) concentrations in combat-related posttraumatic stress disorder. Psychoneuroendocrinology 35, 1001–1007. 10.1016/j.psyneuen.2010.01.001 20116928

[B363] Swanson L. W. Sanchez-Watts G. Watts A. G. (2005). Comparison of melanin-concentrating hormone and hypocretin/orexin mRNA expression patterns in a new parceling scheme of the lateral hypothalamic zone. Neurosci. Lett. 387, 80–84. 10.1016/j.neulet.2005.06.066 16084021

[B364] Swinburn B. A. Sacks G. Hall K. D. Mcpherson K. Finegood D. T. Moodie M. L.. (2011). The global obesity pandemic: shaped by global drivers and local environments. Lancet 378, 804–814. 10.1016/s0140-6736(11)60813-1 21872749

[B366] Szczypka M. S. Rainey M. A. Kim D. S. Alaynick W. A. Marck B. T. Matsumoto A. M.. (1999). Feeding behavior in dopamine-deficient mice. Proc. Natl. Acad. Sci. U S A 96, 12138–12143. 10.1073/pnas.96.21.12138 10518589 PMC18425

[B365] Szczypka M. S. Rainey M. A. Palmiter R. D. (2000). Dopamine is required for hyperphagia in Lep(ob/ob) mice. Nat. Genet. 25, 102–104. 10.1038/75484 10802666

[B367] Szigethy E. Beaudet A. (1989). Correspondence between high affinity 125I-neurotensin binding sites and dopaminergic neurons in the rat substantia nigra and ventral tegmental area: a combined radioautographic and immunohistochemical light microscopic study. J. Comp. Neurol. 279, 128–137. 10.1002/cne.902790111 2563267

[B368] Tabuchi S. Tsunematsu T. Black S. W. Tominaga M. Maruyama M. Takagi K.. (2014). Conditional ablation of orexin/hypocretin neurons: a new mouse model for the study of narcolepsy and orexin system function. J. Neurosci. 34, 6495–6509. 10.1523/jneurosci.0073-14.2014 24806676 PMC4012309

[B369] Taha S. A. Fields H. L. (2006). Inhibitions of nucleus accumbens neurons encode a gating signal for reward-directed behavior. J. Neurosci. 26, 217–222. 10.1523/jneurosci.3227-05.2006 16399690 PMC6674301

[B370] Tanaka K. Masu M. Nakanishi S. (1990). Structure and functional expression of the cloned rat neurotensin receptor. Neuron 4, 847–854. 10.1016/0896-6273(90)90137-5 1694443

[B371] Tang J. Chen J. Ramanjaneya M. Punn A. Conner A. C. Randeva H. S. (2008). The signalling profile of recombinant human orexin-2 receptor. Cell. Signal. 20, 1651–1661. 10.1016/j.cellsig.2008.05.010 18599270

[B372] Teitelbaum P. (1979). This week’s citation classic: teitalbaum P and Epstein AN. The lateral hypothalamic syndrome recovery of feeding and drinking after lateral hypothalamic lesions. Curr. Contents 11:14. 10.1037/h0039285 13920110

[B375] Teitelbaum P. Cheng M. F. Rozin P. (1969). Stages of recovery and development of lateral hypothalamic control of food and water intake. Ann. N Y Acad. Sci. 157, 849–860. 10.1111/j.1749-6632.1969.tb12923.x 5255642

[B373] Teitelbaum P. Epstein A. N. (1962). The lateral hypothalamic syndrome: recovery of feeding and drinking after lateral hypothalamic lesions. Psychol. Rev. 69, 74–90. 10.1037/h0039285 13920110

[B374] Teitelbaum P. Stellar E. (1954). Recovery from the failure to eat produced by hypothalamic lesions. Science 120, 894–895. 10.1126/science.120.3126.894 13216188

[B376] Thannickal T. C. Lai Y. Y. Siegel J. M. (2007). Hypocretin (orexin) cell loss in Parkinson’s disease. Brain 130, 1586–1595. 10.1093/brain/awm097 17491094 PMC8762453

[B377] Thannickal T. C. Moore R. Y. Nienhuis R. Ramanathan L. Gulyani S. Aldrich M.. (2000). Reduced number of hypocretin neurons in human narcolepsy. Neuron 27, 469–474. 10.1016/s0896-6273(00)00058-1 11055430 PMC8760623

[B378] Thorpe A. J. Kotz C. M. (2005). Orexin A in the nucleus accumbens stimulates feeding and locomotor activity. Brain Res. 1050, 156–162. 10.1016/j.brainres.2005.05.045 15979595

[B379] Torrealba F. Yanagisawa M. Saper C. B. (2003). Colocalization of orexin a and glutamate immunoreactivity in axon terminals in the tuberomammillary nucleus in rats. Neuroscience 119, 1033–1044. 10.1016/s0306-4522(03)00238-0 12831862

[B380] Toshinai K. Date Y. Murakami N. Shimada M. Mondal M. S. Shimbara T.. (2003). Ghrelin-induced food intake is mediated via the orexin pathway. Endocrinology 144, 1506–1512. 10.1210/en.2002-220788 12639935

[B381] Toshinai K. Yamaguchi H. Sun Y. Smith R. G. Yamanaka A. Sakurai T.. (2006). Des-acyl ghrelin induces food intake by a mechanism independent of the growth hormone secretagogue receptor. Endocrinology 147, 2306–2314. 10.1210/en.2005-1357 16484324

[B382] Touzani K. Tramu G. Nahon J. L. Velley L. (1993). Hypothalamic melanin-concentrating hormone and alpha-neoendorphin-immunoreactive neurons project to the medial part of the rat parabrachial area. Neuroscience 53, 865–876. 10.1016/0306-4522(93)90631-o 8487959

[B383] Trivedi P. Yu H. Macneil D. J. Van Der Ploeg L. H. Guan X. M. (1998). Distribution of orexin receptor mRNA in the rat brain. FEBS Lett. 438, 71–75. 10.1016/s0014-5793(98)01266-6 9821961

[B384] Tschop M. Smiley D. L. Heiman M. L. (2000). Ghrelin induces adiposity in rodents. Nature 407, 908–913. 10.1038/35038090 11057670

[B385] Tsunematsu T. Kilduff T. S. Boyden E. S. Takahashi S. Tominaga M. Yamanaka A. (2011). Acute optogenetic silencing of orexin/hypocretin neurons induces slow-wave sleep in mice. J. Neurosci. 31, 10529–10539. 10.1523/jneurosci.0784-11.2011 21775598 PMC3864636

[B386] Tsunematsu T. Ueno T. Tabuchi S. Inutsuka A. Tanaka K. F. Hasuwa H.. (2014). Optogenetic manipulation of activity and temporally controlled cell-specific ablation reveal a role for MCH neurons in sleep/wake regulation. J. Neurosci. 34, 6896–6909. 10.1523/JNEUROSCI.5344-13.2014 24828644 PMC4019803

[B387] Tupone D. Madden C. J. Cano G. Morrison S. F. (2011). An orexinergic projection from perifornical hypothalamus to raphe pallidus increases rat brown adipose tissue thermogenesis. J. Neurosci. 31, 15944–15955. 10.1523/jneurosci.3909-11.2011 22049437 PMC3224674

[B388] Tyhon A. Lakaye B. Grisar T. Tirelli E. (2008). Deletion of melanin-concentrating hormone receptor-1 gene accentuates D-amphetamine-induced psychomotor activation but neither the subsequent development of sensitization nor the expression of conditioned activity in mice. Pharmacol. Biochem. Behav. 88, 446–455. 10.1016/j.pbb.2007.10.001 17996928

[B389] Uhl G. R. (1982). Distribution of neurotensin and its receptor in the central nervous system. Ann. N Y Acad. Sci. 400, 132–149. 10.1111/j.1749-6632.1982.tb31565.x 6301323

[B390] Uhl G. R. Kuhar M. J. Snyder S. H. (1977). Neurotensin: immunohistochemical localization in rat central nervous system. Proc. Natl. Acad. Sci. U S A 74, 4059–4063. 10.1073/pnas.74.12.5777 333458 PMC431844

[B391] Uhl G. R. Snyder S. H. (1977). Neurotensin receptor binding, regional and subcellular distributions favor transmitter role. Eur. J. Pharmacol. 41, 89–91. 10.1016/0014-2999(77)90378-8 12987

[B392] Ungerstedt U. (1971). Adipsia and aphagia after 6-hydroxydopamine induced degeneration of the nigro-striatal dopamine system. Acta Physiol. Scand. Suppl. 367, 95–122. 10.1111/j.1365-201x.1971.tb11001.x 4332694

[B393] Vadnie C. A. Hinton D. J. Choi S. Choi Y. Ruby C. L. Oliveros A.. (2014). Activation of neurotensin receptor type 1 attenuates locomotor activity. Neuropharmacology 85C, 482–492. 10.1016/j.neuropharm.2014.05.046 24929110 PMC4107019

[B394] van den Pol A. N. (1999). Hypothalamic hypocretin (orexin): robust innervation of the spinal cord. J. Neurosci. 19, 3171–3182. 10191330 10.1523/JNEUROSCI.19-08-03171.1999 PMC6782271

[B395] Vaughn A. W. Baumeister A. A. Hawkins M. F. Anticich T. G. (1990). Intranigral microinjection of neurotensin suppresses feeding in food deprived rats. Neuropharmacology 29, 957–960. 10.1016/0028-3908(90)90147-j 2255387

[B396] Verret L. Goutagny R. Fort P. Cagnon L. Salvert D. Léger L.. (2003). A role of melanin-concentrating hormone producing neurons in the central regulation of paradoxical sleep. BMC Neurosci. 4:19. 10.1186/1471-2202-4-19 12964948 PMC201018

[B397] Vincent B. Jiracek J. Noble F. Loog M. Roques B. Dive V.. (1997). Contribution of endopeptidase 3.4.24.15 to central neurotensin inactivation. Eur. J. Pharmacol. 334, 49–53. 10.1016/s0014-2999(97)01209-0 9346327

[B398] Vittoz N. M. Berridge C. W. (2006). Hypocretin/orexin selectively increases dopamine efflux within the prefrontal cortex: involvement of the ventral tegmental area. Neuropsychopharmacology 31, 384–395. 10.1038/sj.npp.1300807 15988471

[B399] Vrang N. Larsen P. J. Clausen J. T. Kristensen P. (1999). Neurochemical characterization of hypothalamic cocaine- amphetamine-regulated transcript neurons. J. Neurosci. 19:RC5. 10234051 10.1523/JNEUROSCI.19-10-j0006.1999 PMC6782729

[B400] Wachi M. Okuda M. Togashi S. Miyashita O. Wakahoi T. (1987). Effects of methamphetamine administration on brain neurotensin-like immunoreactivity in rats. Neurosci. Lett. 78, 222–226. 10.1016/0304-3940(87)90637-9 3627560

[B401] Walling S. G. Nutt D. J. Lalies M. D. Harley C. W. (2004). Orexin-A infusion in the locus ceruleus triggers norepinephrine (NE) release and NE-induced long-term potentiation in the dentate gyrus. J. Neurosci. 24, 7421–7426. 10.1523/jneurosci.1587-04.2004 15329388 PMC6729640

[B402] Wang J. Osaka T. Inoue S. (2001). Energy expenditure by intracerebroventricular administration of orexin to anesthetized rats. Neurosci. Lett. 315, 49–52. 10.1016/s0304-3940(01)02322-9 11711212

[B403] Wang H. L. Wu T. (1996). G alpha q/11 mediates neurotensin excitation of substantia nigra dopaminergic neurons. Brain Res. Mol. Brain Res. 36, 29–36. 10.1016/0169-328x(95)00235-k 9011762

[B404] Watts A. G. (1992). Osmotic stimulation differentially affects cellular levels of corticotropin-releasing hormone and neurotensin/neuromedin N mRNAs in the lateral hypothalamic area and central nucleus of the amygdala. Brain Res. 581, 208–216. 10.1016/0006-8993(92)90710-q 1393529

[B405] Watts A. G. Kelly A. B. Sanchez-Watts G. (1995). Neuropeptides and thirst: the temporal response of corticotropin-releasing hormone and neurotensin/neuromedin N gene expression in rat limbic forebrain neurons to drinking hypertonic saline. Behav. Neurosci. 109, 1146–1157. 10.1037//0735-7044.109.6.1146 8748964

[B406] Watts A. G. Sanchez-Watts G. (2007). Rapid and preferential activation of Fos protein in hypocretin/orexin neurons following the reversal of dehydration-anorexia. J. Comp. Neurol. 502, 768–782. 10.1002/cne.21316 17436292

[B407] Watts A. G. Sanchez-Watts G. Kelly A. B. (1999). Distinct patterns of neuropeptide gene expression in the lateral hypothalamic area and arcuate nucleus are associated with dehydration-induced anorexia. J. Neurosci. 19, 6111–6121. 10407047 10.1523/JNEUROSCI.19-14-06111.1999 PMC6783090

[B408] Werkman T. R. Kruse C. G. Nievelstein H. Long S. K. Wadman W. J. (2000). Neurotensin attenuates the quinpirole-induced inhibition of the firing rate of dopamine neurons in the rat substantia nigra pars compacta and the ventral tegmental area. Neuroscience 95, 417–423. 10.1016/s0306-4522(99)00449-2 10658621

[B409] Wienecke M. Werth E. Poryazova R. Baumann-Vogel H. Bassetti C. L. Weller M.. (2012). Progressive dopamine and hypocretin deficiencies in Parkinson’s disease: is there an impact on sleep and wakefulness? J. Sleep Res. 21, 710–717. 10.1111/j.1365-2869.2012.01027.x 22747735

[B410] Wilding J. P. Gilbey S. G. Bailey C. J. Batt R. A. Williams G. Ghatei M. A.. (1993). Increased neuropeptide-Y messenger ribonucleic acid (mRNA) and decreased neurotensin mRNA in the hypothalamus of the obese (ob/ob) mouse. Endocrinology 132, 1939–1944. 10.1210/en.132.5.1939 7682936

[B411] Williams R. H. Alexopoulos H. Jensen L. T. Fugger L. Burdakov D. (2008). Adaptive sugar sensors in hypothalamic feeding circuits. Proc. Natl. Acad. Sci. U S A 105, 11975–11980. 10.1073/pnas.0802687105 18695235 PMC2575303

[B412] Williams G. Cardoso H. Lee Y. C. Ghatei M. A. Flatt P. R. Bailey C. J.. (1991). Reduced hypothalamic neurotensin concentrations in the genetically obese diabetic (ob/ob) mouse: possible relationship to obesity. Metabolism 40, 1112–1116. 10.1016/0026-0495(91)90139-n 1943736

[B413] Williams R. H. Morton A. J. Burdakov D. (2011). Paradoxical function of orexin/hypocretin circuits in a mouse model of Huntington’s disease. Neurobiol. Dis. 42, 438–445. 10.1016/j.nbd.2011.02.006 21324360 PMC5767114

[B414] Willie J. T. Chemelli R. M. Sinton C. M. Tokita S. Williams S. C. Kisanuki Y. Y.. (2003). Distinct narcolepsy syndromes in Orexin receptor-2 and Orexin null mice: molecular genetic dissection of Non-REM and REM sleep regulatory processes. Neuron 38, 715–730. 10.1016/s0896-6273(03)00330-1 12797957

[B415] Willie J. T. Lim M. M. Bennett R. E. Azarion A. A. Schwetye K. E. Brody D. L. (2012). Controlled cortical impact traumatic brain injury acutely disrupts wakefulness and extracellular orexin dynamics as determined by intracerebral microdialysis in mice. J. Neurotrauma 29, 1908–1921. 10.1089/neu.2012.2404 22607167 PMC3390985

[B416] Willie J. T. Sinton C. M. Maratos-Flier E. Yanagisawa M. (2008). Abnormal response of melanin-concentrating hormone deficient mice to fasting: hyperactivity and rapid eye movement sleep suppression. Neuroscience 156, 819–829. 10.1016/j.neuroscience.2008.08.048 18809470 PMC2586720

[B417] Winsky-Sommerer R. Yamanaka A. Diano S. Borok E. Roberts A. J. Sakurai T.. (2004). Interaction between the corticotropin-releasing factor system and hypocretins (orexins): a novel circuit mediating stress response. J. Neurosci. 24, 11439–11448. 10.1523/jneurosci.3459-04.2004 15601950 PMC6730356

[B418] Woods S. C. Lotter E. C. Mckay L. D. Porte D. Jr. (1979). Chronic intracerebroventricular infusion of insulin reduces food intake and body weight of baboons. Nature 282, 503–505. 10.1038/282503a0 116135

[B419] Woulfe J. Beaudet A. (1992). Neurotensin terminals form synapses primarily with neurons lacking detectable tyrosine hydroxylase immunoreactivity in the rat substantia nigra and ventral tegmental area. J. Comp. Neurol. 321, 163–176. 10.1002/cne.903210114 1351897

[B420] Wu X. Gao J. Yan J. Owyang C. Li Y. (2004). Hypothalamus-brain stem circuitry responsible for vagal efferent signaling to the pancreas evoked by hypoglycemia in rat. J. Neurophysiol. 91, 1734–1747. 10.1152/jn.00791.2003 14645380

[B421] Wu M. F. Nienhuis R. Maidment N. Lam H. A. Siegel J. M. (2011). Role of the hypocretin (orexin) receptor 2 (Hcrt-r2) in the regulation of hypocretin level and cataplexy. J. Neurosci. 31, 6305–6310. 10.1523/jneurosci.0365-11.2011 21525270 PMC3097029

[B422] Wu Q. Whiddon B. B. Palmiter R. D. (2012). Ablation of neurons expressing agouti-related protein, but not melanin concentrating hormone, in leptin-deficient mice restores metabolic functions and fertility. Proc. Natl. Acad. Sci. U S A 109, 3155–3160. 10.1073/pnas.1120501109 22232663 PMC3286929

[B423] Xie X. Crowder T. L. Yamanaka A. Morairty S. R. Lewinter R. D. Sakurai T.. (2006). GABA(B) receptor-mediated modulation of hypocretin/orexin neurones in mouse hypothalamus. J. Physiol. 574, 399–414. 10.1113/jphysiol.2006.108266 16627567 PMC1817779

[B424] Xie X. Wisor J. P. Hara J. Crowder T. L. Lewinter R. Khroyan T. V.. (2008). Hypocretin/orexin and nociceptin/orphanin FQ coordinately regulate analgesia in a mouse model of stress-induced analgesia. J. Clin. Invest. 118, 2471–2481. 10.1172/jci35115 18551194 PMC2423866

[B425] Yamada M. Yamada M. Lombet A. Forgez P. Rostene W. (1998). Distinct functional characteristics of levocabastine sensitive rat neurotensin NT2 receptor expressed in Chinese hamster ovary cells. Life Sci. 62, 375–380. 10.1016/s0024-3205(98)00192-1 9627096

[B426] Yamanaka A. Beuckmann C. T. Willie J. T. Hara J. Tsujino N. Mieda M.. (2003). Hypothalamic orexin neurons regulate arousal according to energy balance in mice. Neuron 38, 701–713. 10.1016/s0896-6273(03)00331-3 12797956

[B427] Yamanaka A. Sakurai T. Katsumoto T. Yanagisawa M. Goto K. (1999). Chronic intracerebroventricular administration of orexin-A to rats increases food intake in daytime, but has no effect on body weight. Brain Res. 849, 248–252. 10.1016/s0006-8993(99)01905-8 10592311

[B428] Yamanaka A. Tabuchi S. Tsunematsu T. Fukazawa Y. Tominaga M. (2010). Orexin directly excites orexin neurons through orexin 2 receptor. J. Neurosci. 30, 12642–12652. 10.1523/JNEUROSCI.2120-10.2010 20861370 PMC6633594

[B429] Yamauchi R. Wada E. Kamichi S. Yamada D. Maeno H. Delawary M.. (2007). Neurotensin type 2 receptor is involved in fear memory in mice. J. Neurochem. 102, 1669–1676. 10.1111/j.1471-4159.2007.04805.x 17697051

[B430] Yoon Y. S. Lee H. S. (2013). Projections from melanin-concentrating hormone (MCH) neurons to the dorsal raphe or the nuclear core of the locus coeruleus in the rat. Brain Res. 1490, 72–82. 10.1016/j.brainres.2012.08.022 22967922

[B431] Zahm D. S. (1987). Neurotensin-immunoreactive neurons in the ventral striatum of the adult rat: ventromedial caudate-putamen, nucleus accumbens and olfactory tubercle. Neurosci. Lett. 81, 41–47. 10.1016/0304-3940(87)90337-5 3696473

[B432] Zamir N. Skofitsch G. Bannon M. J. Jacobowitz D. M. (1986). Melanin-concentrating hormone: unique peptide neuronal system in the rat brain and pituitary gland. Proc. Natl. Acad. Sci. U S A 83, 1528–1531. 10.1073/pnas.83.5.1528 3513180 PMC323110

[B433] Zhang J. Li B. Yu L. He Y. C. Li H. Z. Zhu J. N.. (2011). A role for orexin in central vestibular motor control. Neuron 69, 793–804. 10.1016/j.neuron.2011.01.026 21338887

[B434] Zhang W. Sunanaga J. Takahashi Y. Mori T. Sakurai T. Kanmura Y.. (2010). Orexin neurons are indispensable for stress-induced thermogenesis in mice. J. Physiol. 588, 4117–4129. 10.1113/jphysiol.2010.195099 20807795 PMC3002445

[B435] Zhang S. Zeitzer J. M. Sakurai T. Nishino S. Mignot E. (2007). Sleep/wake fragmentation disrupts metabolism in a mouse model of narcolepsy. J. Physiol. 581, 649–663. 10.1113/jphysiol.2007.129510 17379635 PMC2075199

[B436] Zheng H. Patterson L. M. Berthoud H. R. (2005a). Orexin-A projections to the caudal medulla and orexin-induced c-Fos expression, food intake and autonomic function. J. Comp. Neurol. 485, 127–142. 10.1002/cne.20515 15776447

[B437] Zheng H. Patterson L. M. Morrison C. Banfield B. W. Randall J. A. Browning K. N.. (2005b). Melanin concentrating hormone innervation of caudal brainstem areas involved in gastrointestinal functions and energy balance. Neuroscience 135, 611–625. 10.1016/j.neuroscience.2005.06.055 16111819

[B438] Zhu Y. Miwa Y. Yamanaka A. Yada T. Shibahara M. Abe Y.. (2003). Orexin receptor type-1 couples exclusively to pertussis toxin-insensitive G-proteins, while orexin receptor type-2 couples to both pertussis toxin-sensitive and -insensitive G-proteins. J. Pharmacol. Sci. 92, 259–266. 10.1254/jphs.92.259 12890892

[B439] Zhu Y. Yamanaka A. Kunii K. Tsujino N. Goto K. Sakurai T. (2002). Orexin-mediated feeding behavior involves both leptin-sensitive and -insensitive pathways. Physiol. Behav. 77, 251–257. 10.1016/s0031-9384(02)00843-0 12419401

[B440] Zigman J. M. Nakano Y. Coppari R. Balthasar N. Marcus J. N. Lee C. E.. (2005). Mice lacking ghrelin receptors resist the development of diet-induced obesity. J. Clin. Invest. 115, 3564–3572. 10.1172/jci26002 16322794 PMC1297251

